# The SET1 Complex Selects Actively Transcribed Target Genes via Multivalent Interaction with CpG Island Chromatin

**DOI:** 10.1016/j.celrep.2017.08.030

**Published:** 2017-09-05

**Authors:** David A. Brown, Vincenzo Di Cerbo, Angelika Feldmann, Jaewoo Ahn, Shinsuke Ito, Neil P. Blackledge, Manabu Nakayama, Michael McClellan, Emilia Dimitrova, Anne H. Turberfield, Hannah K. Long, Hamish W. King, Skirmantas Kriaucionis, Lothar Schermelleh, Tatiana G. Kutateladze, Haruhiko Koseki, Robert J. Klose

**Affiliations:** 1 Department of Biochemistry, University of Oxford, Oxford, OX1 3QU, UK; 2 Laboratory for Developmental Genetics, RIKEN Center for Integrative Medical Sciences (IMS), 1-7-2 Suehiro-cho, Tsurumi-ku, Yokohama, Kanagawa 230-0045, Japan; 3 Department of Pharmacology, University of Colorado School of Medicine, Aurora, CO 80045, USA; 4 Ludwig Cancer Research, Nuffield Department of Medicine, University of Oxford, Oxford OX3 7DQ, UK

**Keywords:** chromatin, histone methylation, DNA methylation, CpG island, transcription, SET1, H3K4me3, multivalent, histone, epigenetics

## Abstract

Chromatin modifications and the promoter-associated epigenome are important for the regulation of gene expression. However, the mechanisms by which chromatin-modifying complexes are targeted to the appropriate gene promoters in vertebrates and how they influence gene expression have remained poorly defined. Here, using a combination of live-cell imaging and functional genomics, we discover that the vertebrate SET1 complex is targeted to actively transcribed gene promoters through CFP1, which engages in a form of multivalent chromatin reading that involves recognition of non-methylated DNA and histone H3 lysine 4 trimethylation (H3K4me3). CFP1 defines SET1 complex occupancy on chromatin, and its multivalent interactions are required for the SET1 complex to place H3K4me3. In the absence of CFP1, gene expression is perturbed, suggesting that normal targeting and function of the SET1 complex are central to creating an appropriately functioning vertebrate promoter-associated epigenome.

## Highlights

• The CFP1/SET1 complex engages in dynamic and stable chromatin-binding events
• CFP1 uses multivalent chromatin interactions to select active CpG island promoters
• SET1A occupancy at CpG island promoters is predominately defined by CFP1
• CFP1 targets SET1 to shape promoter-associated H3K4me3 and gene expression

## Graphical Abstract

**Figure:**
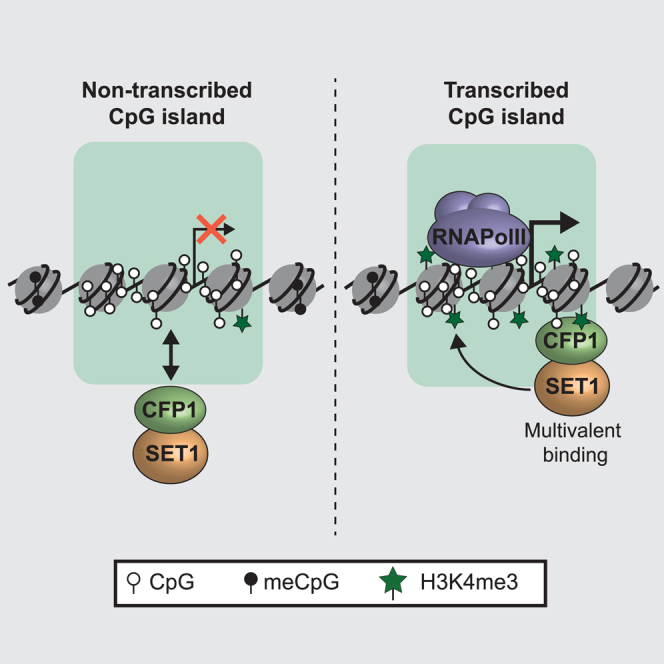

## Teaser

Brown et al. show that the SET1 complex is driven to active CpG island promoters via the CFP1 protein, which engages in multivalent chromatin binding to recognize both non-methylated DNA and H3K4me3. This is necessary for normal H3K4me3 at active gene promoters and appropriate regulation of gene expression.

## Introduction

Gene expression is controlled by transcription factors that bind to DNA sequences in gene regulatory elements and control how RNA polymerase engages with transcription start sites (Levine et al., 2014). However, in eukaryotes, nucleosomes can limit accessibility to DNA sequences and create a barrier to the gene regulatory apparatus (Lorch et al., 1987). To counteract this, post-translational modifications on histones at gene regulatory elements can alter chromatin structure or recruit reader proteins that regulate access to DNA and help to shape gene expression (Piunti and Shilatifard, 2016, Venkatesh and Workman, 2015). Many of the most prevalent histone modifications have been extensively mapped within vertebrate genomes (ENCODE Project Consortium, 2012). However, how the chromatin-modifying complexes that place these modifications recognize their appropriate target sites and affect gene expression remains poorly understood.

Methylation of histone H3 on lysine 4 (H3K4me) is evolutionarily conserved from yeast to human and widely associated with gene regulatory elements, where it is thought to support gene activity (reviewed by Kusch, 2012 and Shilatifard, 2012). H3K4me can occur in distinct states, with monomethylation (me1) predominating at distal regulatory elements and dimethylation (me2) and trimethylation (me3) predominating at active gene promoters in vertebrates (Bernstein et al., 2005, Heintzman et al., 2007, Heintzman et al., 2009, Schneider et al., 2004). H3K4me can be placed by six large multi-protein complexes (van Nuland et al., 2013) that are distinguishable based on the identity of their catalytic subunits, which correspond to MLL1, MLL2, MLL3, MLL4, SET1A, or SET1B (reviewed by Shilatifard, 2012). MLL3/4 complexes deposit H3K4me1 at distal regulatory elements (Hu et al., 2013a, Kaikkonen et al., 2013, Lee et al., 2013), whereas MLL1/2 and SET1A/B are thought to place H3K4me at gene promoters (Andreu-Vieyra et al., 2010, Bledau et al., 2014, Denissov et al., 2014, Milne et al., 2002, Wang et al., 2009). In most cell types, the SET1 complexes are the predominant H3K4 methyltransferases (Ardehali et al., 2011, Bledau et al., 2014, Hallson et al., 2012) and H3K4me is thought to act as a nucleation site for binding of reader proteins that elicit effects on chromatin structure and gene regulation (Lauberth et al., 2013, Li et al., 2006, Peña et al., 2006, Shi et al., 2006, Vermeulen et al., 2007, Wysocka et al., 2006). Deletion of SET1A in mice results in early embryonic lethality due to a failure of embryos to gastrulate, illustrating a fundamental role for the SET1A complex in mammalian development (Bledau et al., 2014).

Attempts to dissect the function of vertebrate H3K4 methyltransferases at gene promoters have been limited by our relatively naive understanding of how these enzymes recognize their target sites in the genome. In budding yeast, the recruitment and activity of the sole H3K4 methyltransferase complex is proposed to rely on an association with RNA polymerase II (RNA PolII) through its phosphorylated C-terminal heptapeptide repeat (CTD) (Ng et al., 2003). This process may be conserved in vertebrates because WDR82, a component of the SET1 complex, has also been proposed to integrate SET1 activity with gene transcription via interaction with the CTD of RNA PolII (Austenaa et al., 2015, Lee and Skalnik, 2008, Wu et al., 2008). However, the relevance of co-transcriptional recruitment to the binding and activity of the vertebrate SET1 complex remains unclear because H3K4 methyltransferase targeting in higher eukaryotes appears to be much more complex. For example, both MLL1/2 and CFP1, a component of the SET1 complex, contain a CXXC DNA-binding domain that can recognize non-methylated CpG dinucleotides found in promoter-associated regulatory elements called CpG islands (CGIs) (Birke et al., 2002, Clouaire et al., 2012, Denissov et al., 2014, Long et al., 2013a, Thomson et al., 2010, Voo et al., 2000). However, generic CGI recognition does not explain why the MLL1/2 and SET1 complexes appear to regulate H3K4me at distinct subsets of target genes in a gene expression-dependent manner (Denissov et al., 2014, Hu et al., 2013b). Furthermore, site-specific DNA binding transcription factors or long non-coding RNAs have also been implicated in recruiting the MLL1/2 and SET1 complexes (Voigt et al., 2013). Therefore, the molecular mechanisms that shape how H3K4 methyltransferases select their target sites remain poorly defined and represent a central conceptual gap in our understanding of the promoter-associated epigenome.

Given the fundamental role that the SET1 complex plays in depositing H3K4me and sustaining normal development, here we have focused on understanding how this complex is targeted to chromatin. By combining live-cell imaging and functional genomics, we discover that the CFP1 component of the SET1 complex preferentially binds to CGIs of actively transcribed genes through multivalent interaction with chromatin, which requires recognition of non-methylated DNA and H3K4me3. We demonstrate that CFP1 is the predominant targeting module for the SET1A complex, whereas co-transcriptional recruitment appears to play only a minor role in SET1A occupancy. Importantly, CFP1 guides H3K4me3 deposition by the SET1A complex and is required for the appropriate expression of a subset of its target genes.

## Results

### Interaction with the SET1 Complex Is the Central Determinant of CFP1 Dynamics In Vivo

**Figure 1:**
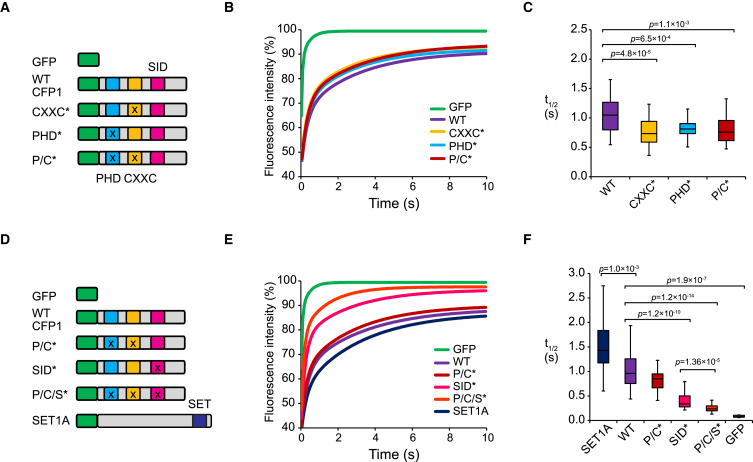
The Cellular Dynamics of CFP1 Are Governed by Its Chromatin-Binding Domains and Association with the SET1A Complex (A) A schematic illustrating the GFP-tagged versions of CFP1 stably expressed in mouse C127 cells and used in the FRAP studies in (B). These include GFP alone (GFP), wild-type CFP1 (WT), CFP1 with a mutated CXXC domain (CXXC^∗^), CFP1 with a mutated PHD domain (PHD^∗^), and CFP1 with combined CXXC and PHD mutations (P/C^∗^). (B) Biexponential fits describing the recovery of fluorescence intensity over time for each of the proteins described in (A). Fits were calculated using post-bleach fluorescence intensity recovery data collected at 8 frames per second from >42 cells across biological triplicates. (C) A boxplot indicating the half time of recovery (t_1/2_) in seconds for the FRAP curves in (B). Boxes show interquartile range (IQR) and whiskers extend by 1.5 × IQR. The p values indicating statistically significant differences are indicated above the boxplot. The p value denotes statistical significance using a Student’s t test. (D) A schematic illustrating the GFP-tagged versions of CFP1 and SET1A used in the FRAP studies in (E). This includes GFP alone (GFP), wild-type CFP1 (WT), CFP1 with combined PHD and CXXC mutations (P/C^∗^), CFP1 with a mutated SET1 interaction domain (SID^∗^), CFP1 with combined PHD/CXXC/SID mutations (P/C/S^∗^), and wild-type SET1A. (E) Biexponential fits describing the recovery of fluorescence intensity over time for the proteins described in (D). CFP1 and SET1 fits were calculated using post-bleach fluorescence intensity recovery data collected at 13 frames per second from >28 cells across biological triplicates, whereas the GFP fit was limited to 8 cells, in which the nuclear border was clearly defined. (F) A boxplot indicating the half time of recovery (t_1/2_) in seconds for the FRAP curves in (E). Boxes show IQR and whiskers extend by 1.5 × IQR. The p values indicating statistically significant differences are indicated above the boxplot. The p value denotes statistical significance using a Student’s t test.

It has been proposed that CFP1 plays a key role in regulating SET1 complex function. This is based on work that described CFP1 occupancy at CGI elements (Denissov et al., 2014, Thomson et al., 2010) and defects in H3K4me3 resulting from its deletion in embryonic stem cells (ESCs) (Carlone et al., 2005, Clouaire et al., 2012, Clouaire et al., 2014, Tate et al., 2009). However, how CFP1 dynamics and chromatin binding are achieved in vivo, and whether these are central determinants in guiding the SET1 complex to genomic target sites, remains largely unknown. To begin addressing these questions, we stably expressed GFP-CFP1 in a mouse epithelial cell line (Figures S1A–S1D) that is suited to live-cell imaging, and examined the mobility of nuclear CFP1 by fluorescence recovery after photobleaching (FRAP). This revealed that CFP1 is highly mobile, with t_1/2_ recovery times in the nucleoplasm of ∼1 s (Figures 1A–1C and S1F–S1I). To understand if CFP1 dynamics are determined by its capacity to interact with chromatin, we engineered single amino acid mutations into CFP1 that disrupt either the function of its non-methylated DNA-binding CXXC domain or its PHD domain, which is proposed to bind to H3K4me (Figures 1A and S2) (Eberl et al., 2013, Mahadevan and Skalnik, 2016). Cell lines were established, in which individual GFP-CFP1 mutants were stably expressed at comparable levels to wild-type GFP-CFP1, and, importantly, we verified that these mutations did not affect association of CFP1 with the SET1A complex (Figures S1A–S1E). Interestingly, mutation of the CXXC or PHD domain caused a small but significant increase in the mobility of CFP1 compared to wild-type protein, but this was not further increased by mutation of both domains (Figures 1B and 1C).

When we examined the dynamics of GFP-SET1A, we observed that it was slightly less mobile than GFP-CFP1 (Figures 1D, S1A, and S1B), consistent with a possible role for the SET1A interaction in limiting CFP1 mobility independent of the CXXC and PHD domains (Figure 1E). Therefore, we generated a GFP-CFP1 SET1 interaction domain (SID) mutant cell line, in which CFP1 association with SET1A was disrupted by a two amino acid substitution mutation in an alpha helix of the previously mapped interaction domain (Figures 1D, S1A–S1E, and S2E) (Tate et al., 2009). Strikingly, when compared to wild-type CFP1, the CFP1 SID mutant was dramatically more mobile (Figure 1E). Importantly, combining the CXXC and PHD mutations with the SID mutation further increased CFP1 mobility to the point where it approached the diffusion of free GFP (Figures 1D–1F). Together, this demonstrates that inclusion in the SET1 complex predominates in defining the nuclear dynamics of CFP1, with the CXXC and PHD domains making additional, more modest contributions.

### Targeting of CFP1 to CGIs Relies on the CXXC and PHD Domains but Not Interaction with SET1

**Figure 2:**
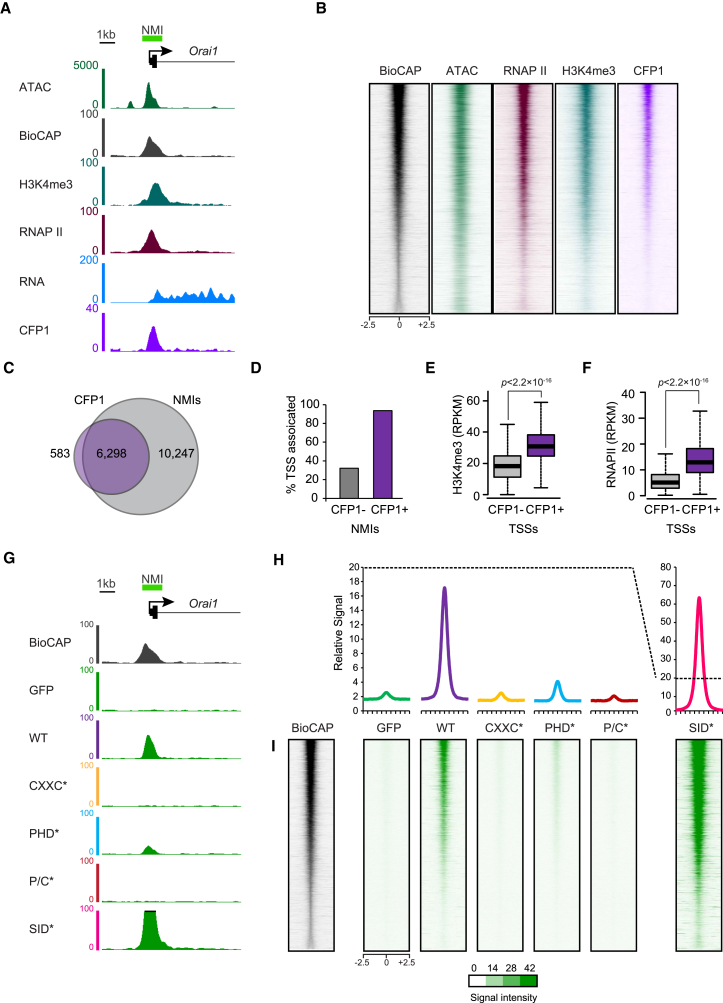
Binding of CFP1 to Chromatin Relies on the CXXC and PHD Domains but Not Interaction with SET1 (A) A genomic snapshot of the NMI-associated *Orai1* gene promoter showing the signal from ATAC, Bio-CAP, H3K4me3, RNA Pol II, RNA, and CFP1 sequencing experiments. (B) Heatmaps of the sequencing signals in (A) ranked by Bio-CAP signal over all NMIs. (C) A Venn diagram illustrating the overlap between CFP1 peaks and NMIs. (D) Bar graph illustrating the percentage of CFP1-bound and -unbound NMIs that overlap with transcription start sites (TSSs). (E) Boxplots illustrating the enrichment of H3K4me3 at TSS-associated NMIs that are bound (CFP1+) or unbound (CFP1−) by CFP1. Boxes show IQR and whiskers extend by 1.5 × IQR. The p value denotes statistical significance calculated by a Wilcoxon signed rank test. (F) As in (E), illustrating enrichment of RNA PolII. (G) A genomic snapshot of the NMI-associated *Orai1* gene promoter showing the GFP ChIP-seq signal for GFP, GFP-CFP1, and mutated forms of GFP-CFP1. (H) Metaplot and heatmap analysis of GFP ChIP-seq signal for GFP, GFP-CFP1, and mutated forms of GFP-CFP1 over all NMIs. (I) Heatmap analysis of GFP ChIP-seq signal over all NMIs for the same cell lines as in (H).

We next wanted to understand how individual domains of CFP1 contribute to the more stable binding of CFP1 at target sites on the genome. To achieve this, we used chromatin immunoprecipitation sequencing (ChIP-seq) to map CFP1 binding genome-wide (Figures 2A and 2B). In parallel, Bio-CAP-seq was used to map non-methylated islands (NMIs), which generally correspond to CGIs (Blackledge et al., 2012, Illingworth et al., 2010, Long et al., 2013b). This revealed that 91.5% of CFP1 peaks occurred at NMIs, but only 38.1% of NMIs were occupied by CFP1 (Figure 2C), indicating that NMI occupancy of CFP1 is not uniform, as described previously (Denissov et al., 2014). Further analysis revealed that CFP1 was enriched at NMIs that had features usually associated with active transcription start sites (TSSs), including H3K4me3 and RNA PolII (Figures 2A and 2B). Indeed, CFP1-bound NMIs were more frequently associated with annotated TSSs and had elevated H3K4me3 and RNA PolII compared to NMI TSSs not bound by CFP1 (Figures 2D–2F).

To explore the determinants of CFP1 binding, we carried out ChIP-seq using a GFP-specific antibody in lines stably expressing GFP alone, wild-type GFP-CFP1, or GFP-CFP1 with mutations in the CXXC, PHD, CXXC/PHD, or SID (Figures 2G–2I). Importantly, GFP-CFP1 enrichment correlated well with endogenous CFP1 (Figure S3). Strikingly, mutating the CXXC domain completely abrogated CFP1 association with NMIs, whereas mutation of the PHD domain also resulted in a dramatic but not complete loss of NMI occupancy. Surprisingly, mutation of the SID resulted in the opposite effect, causing CFP1 to bind more efficiently to NMIs (Figures 2G–2I). Together, our observations suggest that DNA and chromatin binding by the CXXC and PHD domains define stable CFP1 accumulation at NMI target sites, whereas SET1 association, which predominates in shaping CFP1 nuclear dynamics, limits accumulation at these regions (see Discussion).

### Multivalent Binding to Non-methylated DNA and H3K4me3 Determines the Occupancy of CFP1 on Chromatin In Vivo

**Figure 3:**
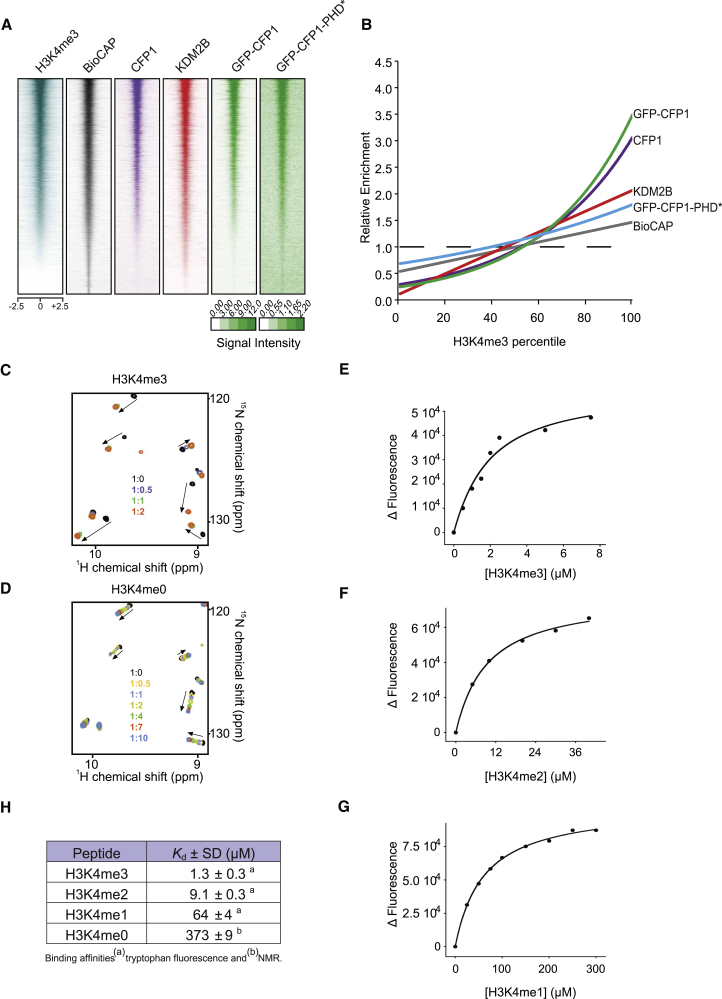
Multivalent Binding to Non-methylated DNA and H3K4me3 Determines the Occupancy of CFP1 on Chromatin In Vivo (A) Heatmap analysis of Bio-CAP and ChIP-seq signal over all NMIs for the indicated endogenous or GFP fusion proteins. The intensity scale for GFP-CFP1 and GFP-CFP1-PHD^∗^ mutant are indicated below. (B) The relative enrichment of the features heatmapped at NMIs in (A) was plotted across H3K4me3 enrichment percentiles. (C) ^1^H,^15^N heteronuclear single quantum coherence titration experiments using the CFP1 PHD domain and an H3K4me3 peptide. (D) As in (C) for an unmodified H3K4me0 peptide. (E–G) Measurements using intrinsic fluorescence spectroscopy for the PHD domain binding to (E) H3K4me3, (F) H3K4me2, and (G) H3K4me1. (H) A table illustrating the quantitative measurements of binding affinities for the CFP1 PHD domain bound to H3K4 substrates, as determined by intrinsic fluorescence spectroscopy and NMR spectroscopy.

The CFP1 CXXC domain is known to bind non-methylated DNA (Voo et al., 2000, Xu et al., 2011), and the PHD domain has been reported to bind H3K4me (Eberl et al., 2013). Therefore, we set out to examine whether CFP1 utilizes a combination of its CXXC and PHD domains to select NMIs that have both non-methylated DNA and H3K4me. If this was the case, we hypothesized that CFP1 binding to NMIs would be related to H3K4me3 levels and differ from stereotypical NMI-binding proteins like KDM2B that only read non-methylated DNA (Blackledge et al., 2010, Blackledge et al., 2014, Farcas et al., 2012). To test this possibility, we binned all NMIs by their H3K4me3 percentile and plotted the relative enrichment of non-methylated DNA (Bio-CAP), CFP1, GFP-CFP1, and KDM2B signal over these regions (Figures 3A and 3B). As expected, KDM2B occupancy scaled nearly linearly with non-methylated DNA despite increasing H3K4me3 (Figure 3B). In contrast, CFP1 and GFP-CFP1 diverged from this linearity with increased occupancy at NMIs with elevated H3K4me3 (Figure 3B). Importantly, however, residual ChIP-seq signal in the GFP-CFP1 PHD mutant exhibited a near-linear relationship with non-methylated DNA (Figures 3A and 3B).

Our observations indicate that CFP1 preferentially binds to H3K4me3-enriched NMIs via its PHD domain (Figures 3A and 3B). However, given that PHD domains can display varying affinities for individual methylation states (Musselman et al., 2012), we were keen to better characterize the binding preference of the CFP1 PHD domain. To achieve this, we generated the recombinant CFP1-PHD domain and examined its binding to unmodified (me0), me1, me2, and me3 H3K4 peptides in vitro (Figures 3C–3H). First, we examined the binding of the CFP1 PHD finger to a H3K4me3 peptide in ^1^H,^15^N heteronuclear single quantum coherence titration experiments. Addition of the H3K4me3 peptide to the ^15^N-labeled CFP1 PHD domain induced substantial chemical shift perturbations, suggesting a tight interaction of the CFP1 PHD domain with H3K4me3. In contrast, interaction with H3K4me0 was considerably weaker, as evident from small chemical shift perturbations and fast exchange (Figure 3D). Quantitative measurements of binding affinities using intrinsic fluorescence spectroscopy (Figures 3E–3G) further supported the NMR results and revealed that the CFP1 PHD domain binds preferentially to the H3K4me3 (Kd 1.3 μM), with H3K4me2/me1/me0 having considerably lower affinities (9.1 μM, 64 μM, and 373 μM, respectively) (Figure 3H). Interestingly, the in vitro affinity of the PHD domain for H3K4me3 is of similar magnitude to that of the isolated CXXC domain for non-methylated DNA (Kd ∼2.5–4.4 μM) (Risner et al., 2013, Xu et al., 2011), further supporting the idea that binding to both H3K4me3 and non-methylated DNA are important affinity features that define normal CFP1 occupancy at NMI target sites. Therefore, our in vivo ChIP-seq analysis demonstrates that the PHD domain is required for the appropriate enrichment of CFP1 at NMIs with elevated H3K4me3 (Figures 3A and 3B) and our in vitro analysis reveals that the PHD domain of CFP1 preferentially binds to H3K4me3.

### CFP1 Is the Central Determinant in SET1A Occupancy on Chromatin

**Figure 4:**
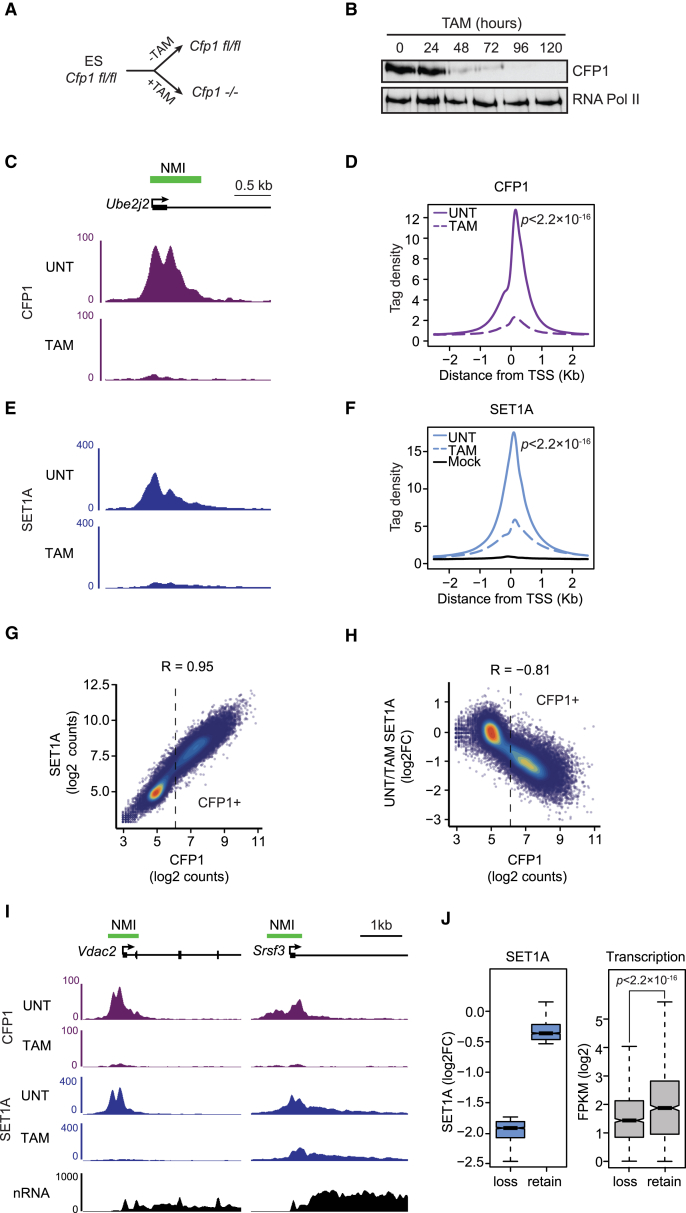
CFP1 Is the Central Determinant in SET1A Occupancy on Chromatin (A) A schematic illustrating the *Cfp1*^*fl/fl*^ mouse ESC model, in which the addition of tamoxifen (TAM) leads to deletion of CFP1. (B) Western blot analysis of CFP1 following a time course of TAM treatment. (C) A genomic snapshot illustrating CFP1 ChIP-seq signal at the *Ube2j2* gene in untreated (upper panel) and tamoxifen-treated cells (lower panel). (D) Metaplot analysis of CFP1 ChIP-seq signal at CFP1-bound NMI-associated TSSs in untreated (solid line) and tamoxifen-treated cells (dashed line). p values denote statistical significant calculated by a Wilcoxon signed rank test comparing read counts across the represented interval in UNT versus TAM. (E) A genomic snapshot illustrating SET1A ChIP-seq signal at the at *Ube2j2* gene in untreated (upper panel) and tamoxifen-treated *Cfp1*^*fl/fl*^ ESCs (lower panel). (F) Metaplot analysis of T7-SET1A ChIP-seq signal at CFP1-bound NMI-associated TSSs in untreated (solid blue line) and tamoxifen-treated *Cfp1*^*fl/fl*^ ESCs (dashed blue line). The solid black line illustrates ChIP-seq signal for the T7 antibody in an untagged cell line (Mock). p values denote statistical significance calculated by a Wilcoxon signed rank test comparing read counts across the represented interval in UNT versus TAM. (G) A scatterplot of the SET1A and CFP1 ChIP-seq signal at TSSs. R value indicates Spearman rank correlation. Genes right of the dashed line correspond to CFP1-bound (CFP1+) sites. (H) A scatterplot of the log2-fold change in SET1A ChIP-seq signal compared to CFP1 ChIP-seq signal at TSSs. R value indicates Spearman rank correlation. Genes right of the dotted line correspond to CFP1-bound (CFP1+) sites. (I) Genomic snapshots illustrating a gene where SET1A ChIP-seq signal is lost following removal of CFP1 (left panel) and a gene that is more highly transcribed and retains some SET1A (right panel) following removal of CFP1. (J) Boxplots illustrating the log2-fold change in SET1A ChIP-seq signal (left panel) and expression level based on 4SU-RNA-seq (right panel) of the top 10% of genes that lose or retain SET1A. Boxes show IQR and whiskers extend by 1.5 × IQR. The p value denotes statistical significance calculated by the Mann-Whitney test.

Previous CFP1 studies have utilized a mouse ESC line isolated from a *Cfp1*^−/−^ embryo. However, these *Cfp1*^−/−^ ESCs display global reductions in genomic DNA methylation and other epigenetic defects (Carlone et al., 2005, Clouaire et al., 2012), presumably due to prolonged culture without CFP1. To overcome this limitation, we derived an ESC line from a CFP1 conditional knockout mouse (*Cfp1*^*fl/fl*^), in which addition of tamoxifen induces C*fp1* deletion (Figures 4A and S4A). Following 96 hr of tamoxifen treatment, CFP1 protein and target site occupancy was undetectable (Figures 4B–4D and S4H), but no global effects on DNA methylation were observed (Figure S4B).

Because CFP1 forms a complex with SET1A (Lee and Skalnik, 2005, van Nuland et al., 2013), we wanted to use the *Cfp1*^*fl/fl*^ ESCs to understand whether CFP1 contributes to SET1A occupancy on chromatin. The genome-wide occupancy of SET1A has remained poorly defined, and we were unable to ChIP SET1A using commercially available antibodies. We therefore used CRISPR/Cas9 technology to engineer epitope tags onto both copies of the *Set1a* gene in the *Cfp1*^*fl/fl*^ ESCs (Figures S4C–S4E). ChIP-seq for epitope-tagged SET1A in the untreated *Cfp1*^*fl/fl*^ ESCs revealed that SET1A and CFP1 occupancy at TSSs was highly correlated (R = 0.95) and SET1A enrichment was greatest at CFP1-bound NMI TSSs (Figures 4E–4G and S4I). Strikingly, following tamoxifen treatment to remove CFP1, we observed a major reduction in SET1A occupancy (Figures 4E, 4F, 4H, and S4G), with little alteration to SET1A protein levels (Figure S4F). Importantly, loss of SET1A binding was highly correlated with the initial level of CFP1 at individual sites (R = −0.81) (Figure 4H). Interestingly, following removal of CFP1, highly transcribed genes exhibited some SET1A retention (Figures 4I, 4J, and S4J). This suggests that at some target sites, a secondary targeting modality contributes to SET1A occupancy. This may involve co-transcriptional recruitment of SET1A via direct interaction with RNA PolII or other features of these genes. Nevertheless, our observations indicate that SET1A is primarily recruited to chromatin by CFP1.

### CFP1 Exploits Multivalent Interactions with CGI Chromatin to Shape H3K4me3

**Figure 5:**
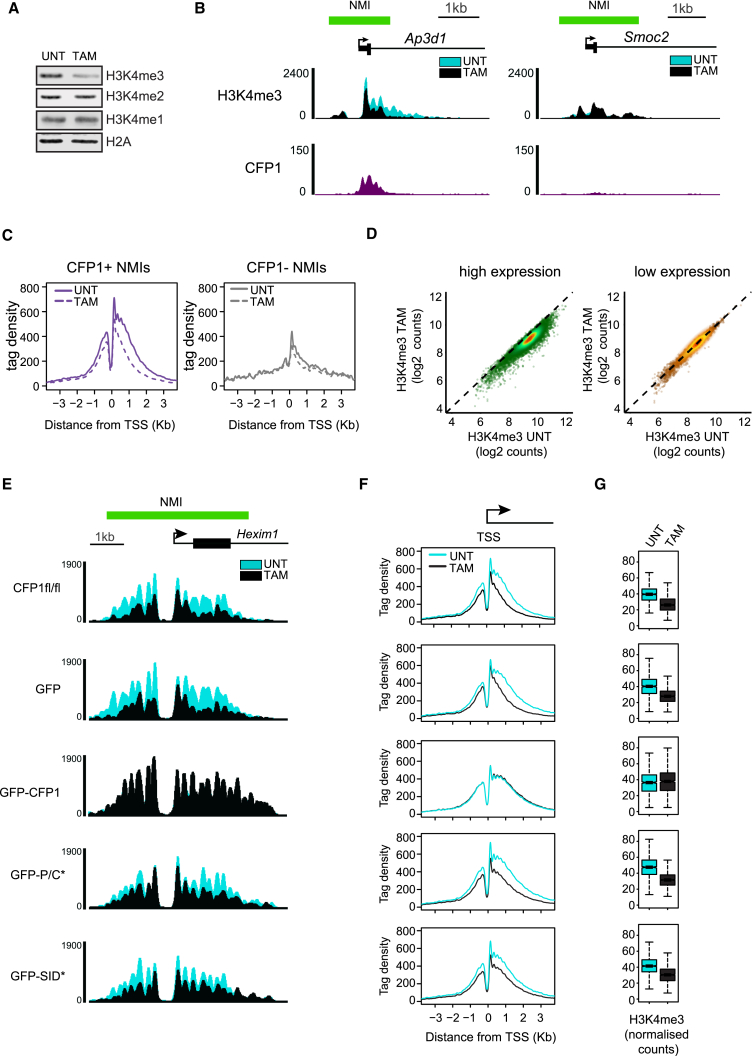
CFP1 Exploits Multivalent Interactions with CpG Island Chromatin to Shape H3K4me3 (A) Western blot analysis of bulk H3K4me in untreated (UNT) and TAM-treated *Cfp1*^*fl/fl*^ mouse ESCs. (B) Genomic snapshot illustrating H3K4me3 ChIP-seq signal at the *Ap3d1* NMI, which is bound by CFP1 (left panel) and the *Smoc2* NMI that is not bound by CFP1 (right panel). The H3K4me3 ChIP-seq signal without tamoxifen treatment is represented in blue (UNT) and the signal with tamoxifen treatment is represented in black (TAM) in the overlay. (C) H3K4me3 signal around CFP1+ (left) and CFP1− (right) NMI-associated TSSs in untreated (solid line) and tamoxifen-treated cells (dashed line). (D) A scatterplot illustrating that highly (left panel) but not lowly (right panel) expressed genes lose H3K4me3 at their TSS following tamoxifen treatment. The scatterplots correspond to non-divergent genes with an H3K4me3 peak overlapping their TSS, and a gene was considered to be lowly expressed if it had less than −2.5 log2 FPKM nuclear RNA seq signal over the gene body. (E) Genomic snapshot illustrating H3K4me3 ChIP-seq signal at the *Hexim1* NMI in UNT and TAM-treated cells. The UNT and TAM-treated samples are colored blue and black in the overlay, respectively. The upper panel corresponds to the parental *Cfp1*^*fl/fl*^ line, with the lower panels corresponding to the parental line rescued with at GFP, GFP-CFP1, GFP-P/C^∗^, or GFP-SID^∗^ transgenes. (F) Metaplot and boxplot analysis of the H3K4me3 signal at TSSs as described in (C) for cell lines described in (E) without and with tamoxifen treatment. (G) Boxplot analysis of H3K4me3 signal at CFP1+ NMI-associated TSSs +/- 1 kb. Boxes show IQR and whiskers extend by 1.5 × IQR.

We next wanted to examine if loss of CFP1-dependent recruitment affected the ability of the SET1A complex to place H3K4me. Western blot analysis of bulk H3K4me revealed that H3K4me3 was reduced in CFP1-deleted cells, whereas H3K4me1 and H3K4me2 were largely unaffected (Figure 5A). To understand more about where H3K4me3 was lost in the genome, we carried out native ChIP-seq for H3K4me3 in untreated and tamoxifen-treated *Cfp1*^*fl/fl*^ ESCs using a calibrated approach (Figures 5B and 5C) (Hu et al., 2015). This revealed that H3K4me3 loss was most pronounced at the TSSs of NMI-associated genes that had broad peaks of H3K4me3 and higher levels of CFP1 binding (Figures S5A–S5C). Furthermore, consistent with CFP1 occupying actively transcribed gene promoters, effects on H3K4me3 following CFP1 loss were prevalent at highly transcribed, as opposed to lowly or non-transcribed, genes (Figure 5D) and also at genes with high RNA PolII occupancy (Figure S5D). This is in agreement with previous observations suggesting that CFP1 contributes to H3K4me3 at actively transcribed genes (Clouaire et al., 2012, Clouaire et al., 2014).

We were next keen to understand if normal H3K4me3 levels relied on the capacity of CFP1 to engage in multivalent chromatin interactions and bind to SET1. We therefore performed a series of H3K4me3 ChIP-seq experiments in tamoxifen-treated *Cfp1*^*fl/fl*^ ESCs that had been rescued with either wild-type or mutant forms of CFP1 (Figures S5E–S5G). Focusing our analysis on NMIs with broad H3K4me3 peaks that are most reliant on CFP1, we observed that GFP expression alone was unable to rescue H3K4me3 defects, whereas GFP-CFP1 fully restored H3K4me3 (Figures 5E–5G). In contrast, mutation of the CXXC/PHD domains or the SID failed to rescue H3K4me3 defects (Figures 5E–5G). This demonstrates that the capacity of CFP1 to engage in multivalent interactions with chromatin and to associate with SET1 is required for normal deposition of H3K4me3.

### Loss of CFP1 Leads to Widespread Effects on Gene Expression

**Figure 6:**
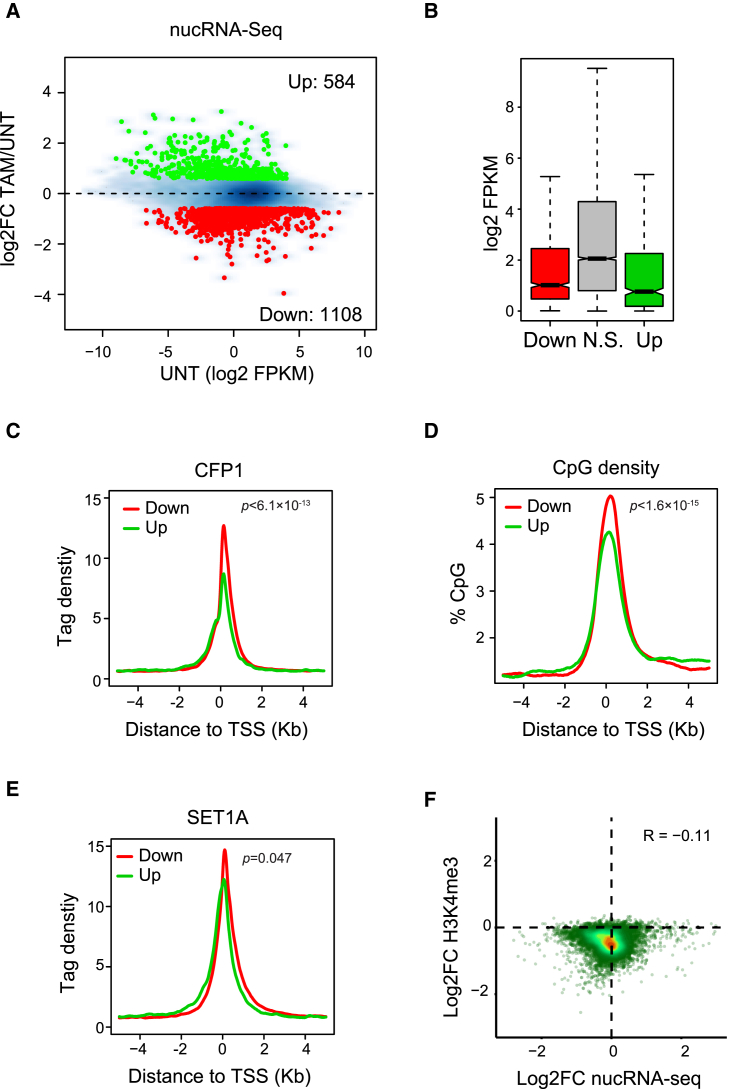
Loss of CFP1 Leads to Widespread Effects on Gene Expression (A) An MA plot showing log2-fold change in the nuclear RNA-seq signal of CFP1-bound NMI-associated genes in UNT and TAM-treated *Cfp1*^*fl/fl*^ cells. Red and green points depict significantly downregulated (1,108) and upregulated genes (584) that change in expression by more than 1.5-fold. (B) Boxplots indicating the expression of genes that are downregulated (red), not significantly (N.S.) changing (gray), or upregulated (green). (C–E) Mean distribution of CFP1 ChIP-seq signal (C), CpG density (D), and SET1A ChIP-seq signal (E) around TSSs of downregulated (red) and upregulated (green) genes. p values denote statistical significance calculated by Mann-Whitney test comparing ChIP-seq read counts across a 200-bp interval flanking the TSS in downregulated versus upregulated genes. (F) Correlation density plot of changes in gene expression (nucRNA-seq) and H3K4me3 at TSSs of CFP1-bound NMI genes. Only genes whose TSSs overlap an H3K4me3 peak and do not have a divergent TSS within 2 kb were considered. R value indicates Spearman rank correlation.

Although H3K4me3 enrichment at gene promoters correlates with gene expression (Kusch, 2012), the contribution of H3K4me3 to transcription remains unclear. To examine the influence of CFP1 on gene expression, we carried out quantitative nuclear RNA sequencing (RNA-seq) in *Cfp1*^*fl/fl*^ ESCs with or without tamoxifen treatment. We focused our analysis on CFP1-bound NMI-associated genes (9,979) and identified significantly misregulated genes (false discovery rate [FDR] ≤ 0.01) that had changes in expression of at least 1.5-fold. Importantly, following CFP1 deletion, CFP1 target genes predominantly exhibited reduced expression (1,108), whereas a smaller subset (584) showed increased expression (Figures 6A, S6B, and S6C). In contrast, genes not bound by CFP1 showed a similar number of increases and decreases in gene expression (Figure S6A). These trends appear to support a role for CFP1 in potentiating gene expression. However, given the appreciable number of CFP1 target genes that are upregulated in response to CFP1 deletion, this may point to uncharacterized roles for CFP1/SET1 in gene repression or to secondary effects on gene expression.

Interestingly, when compared to unaffected genes, CFP1 target genes with reduced expression tended to be lowly to moderately expressed (Figure 6B). Furthermore, when compared to upregulated CFP1 target genes, they had elevated CpG density and CFP1 at their TSSs (Figures 6C and 6D) as well as moderately higher levels of SET1A (Figure 6E). However, we found no obvious correlation between the reductions in gene expression and effects on H3K4me3 following removal of CFP1 (R = −0.11) (Figure 6F), indicating that the level of H3K4me3 loss does not define how the associated gene will respond transcriptionally. Together, these observations indicate that the CFP1/SET1 complex plays an important role in shaping gene expression from transcribed CGI-associated genes, with moderately expressed genes appearing more sensitive to CFP1 loss than more highly expressed genes (see Discussion).

## Discussion

Here, we discover that CFP1, a component of the SET1 complex, uses a type of multivalent interaction with chromatin, which relies on its CXXC and PHD domains to identify target gene promoters (Figures 1 and 2). This allows CFP1 to recognize actively transcribed CGI-associated genes that have non-methylated DNA and elevated H3K4me3 (Figures 2 and 3). Importantly, CFP1-based targeting, as opposed to co-transcriptional recruitment, predominates in defining occupancy of the SET1A complex at target sites (Figure 4). An inability of CFP1 to engage in multivalent interactions with CGI chromatin leads to reductions in H3K4me3 at gene promoters (Figure 5), and loss of CFP1 leads predominantly to reductions in transcription at CFP1-occupied genes (Figure 6). Together, this reveals a central role for CFP1 in recruiting the SET1A complex to shape the promoter-associated epigenome and regulate gene expression.

The mechanisms by which chromatin-modifying factors bind to chromatin and identify their target sites represent a major conceptual gap in our understanding of how the epigenome is formed and regulated. To study this, FRAP can be used to capture dynamic chromatin interactions that are often too rapid to be effectively observed by crosslinking and ChIP (Schmiedeberg et al., 2009), but has limited spatial resolution with respect to the genome. Conversely, ChIP captures more stable binding events with high spatial resolution, even when these represent a small fraction of the total protein molecules in the nucleus. Therefore, combining these approaches, as we have done here, can reveal dynamics and chromatin-binding characteristics across a wide spectrum of temporal and spatial resolutions. Interestingly, our FRAP analysis revealed that CFP1 is a highly dynamic protein and that these dynamics are predominantly driven by inclusion in the SET1 complex. We propose that these dynamics are dictated by low affinity, widespread, and non-specific interactions that the SET1 complex makes with chromatin, which are not effectively captured by ChIP. A mutant form of CFP1 that does not interact with SET1 binds more effectively to CGIs, presumably because it no longer engages in other SET1-dependent interactions with the genome. Conversely, mutation of the CXXC/PHD domains abrogates more stable binding to CGIs, as is evident from loss of ChIP-seq signal at these regions, yet this contributes modestly to FRAP dynamics. This suggests that a small proportion of the total pool of CFP1 molecules in the cell is stably bound to CGIs at any one time. In other words, CFP1 as part of the SET1 complex appears to engage in two general modes of chromatin binding: one that is dynamic, possibly widespread, and SET1 dependent, and a second that is more stable, localized to CGIs, and reliant on multivalent CXXC/PHD domain-dependent interactions with CGI chromatin. It is unclear whether the dynamic pool of CFP1/SET1 observed in FRAP is of functional relevance, but it is possible that this could represent SET1 complex search mechanisms or non-CGI localized processes that the SET1 complex engages in. To further dissect these binding features, new live-cell single molecule approaches (Chen et al., 2014) will be required to track individual CFP1/SET1 molecules and discover how they navigate the nucleus to identify their target sites.

SET1 complexes are thought to play a prominent role in depositing all three H3K4 methylation states (Bledau et al., 2014), yet removal of CFP1 leads primarily to a reduction in H3K4me3 (Figure 5) (Clouaire et al., 2012, Tate et al., 2009). Notably, deletion of the budding yeast ortholog of CFP1, Spp1, also results in a predominant loss of H3K4me3 (Kim et al., 2013, Schlichter and Cairns, 2005). This suggests that regulating the transition to H3K4me3 is an evolutionarily conserved function of CFP1/Spp1, and that deposition of lower H3K4 methylation states by the SET1 complex can occur independently of these factors. It has been proposed that H3K4me3 functions to create transcriptionally permissive chromatin at gene promoters (Voigt et al., 2013). Interestingly, however, we identified only a subset of genes that have reduced expression in the absence of CFP1 (Figure 6), despite global reductions in H3K4me3 at actively transcribed genes (Figure 5). Furthermore, the level of H3K4me3 reduction did not correlate with the effects on transcription, suggesting the level of H3K4me3 and capacity to transcribe are not inextricably linked. Therefore, we favor the possibility that CFP1/SET1 elevates H3K4me3 at actively transcribed genes, with the transcription of some genes being more sensitive to loss of this chromatin modification than others, in agreement with recent observations suggesting that the effects H3K4me3 has on gene expression are context dependent (Cano-Rodriguez et al., 2016). The sensitivity of individual genes to the loss of CFP1/SET1 activity may be related to the nature of their transcriptional inputs. Indeed, we observe that more moderately, as opposed to highly, transcribed genes appear to be misregulated in the absence of CFP1 (Figures 6A and 6B). A possible explanation for this may be that moderately expressed genes are subject to weak activation signals, meaning that chromatin features have more of an influence on transcriptional output. At such genes, loss of CFP1/SET1 activity and the formation of H3K4me3-depleted chromatin may create a greater barrier to transcription. In contrast, at more highly expressed CFP1 target genes, stronger activation signals may render chromatin features less influential.

Genome-wide studies have revealed that individual CGIs generally exist in one of two chromatin states: either having high levels of H3K4me3 and being actively transcribed or being lowly or non-transcribed and having repressive Polycomb group protein (PcG)-associated histone modifications (Blackledge et al., 2015). Given that H3K4 methyltransferase and PcG protein complexes contain CGI-binding domains, we and others have previously proposed that these opposing systems may dynamically sample CGIs in order to respond to the transcriptional state of the associated gene and resolve individual gene regulatory elements into transcriptionally permissive or repressive chromatin states (Blackledge et al., 2015, Deaton and Bird, 2011, Klose et al., 2013, Steffen and Ringrose, 2014, Voigt et al., 2013). Although feedback mechanisms inherent to the PcG protein complexes appear to be sufficient to achieve repressive chromatin states in the absence of gene transcription (Blackledge et al., 2014, Riising et al., 2014), the mechanisms leading to the recruitment and stabilization of H3K4 methyltransferases at actively transcribed genes have remained elusive. Our new observations detailing SET1 targeting in vivo suggest that CFP1 guides SET1A to CGIs that have non-methylated DNA and H3K4me3 to ensure formation of the H3K4me3-predominating state and support normal gene expression.

Although our observations provide a potentially simple explanation for how the CFP1/SET1 complex regulates H3K4me3 at actively transcribed CGI-associated genes, it remains less clear how the H3K4me3-predominating state would be initiated in the first place, given that the CFP1/SET1 complex must recognize pre-existing H3K4me3. One possibility is that the CFP1/SET1 complex binds to CGIs where the MLL1/2 complexes have already initiated low-level deposition of H3K4me3. However, removal of MLL1/2 has little effect on the levels of H3K4me3 at actively transcribed genes in ESCs, suggesting that the MLL1/2 and SET1 complexes do not act in a simple linear pathway or that MLL1/2 is not required to maintain elevated H3K4me3 once it has been initiated (Denissov et al., 2014, Hu et al., 2013b). Alternatively, the process of initiating gene transcription could result in transient co-transcriptional recruitment of the SET1 complex to seed low levels of H3K4me3 at actively transcribed sites and provide a signal that stabilizes multivalent binding by CFP1/SET1. In support of the latter possibility, we observe residual SET1A occupancy at highly transcribed genes in the absence of CFP1 (Figure 4). Furthermore, initiation and formation of the H3K4me3-predominating state appears to be required for the normal expression of some genes (Figure 6). It is therefore tempting to speculate that the SET1 complex could form part of a simple activity-based epigenetic switch, whereby transcription initiation leads to low-level H3K4me3 deposition at CGIs. This could in turn support multivalent binding of the CFP1/SET1 complex, amplification of the H3K4me3-predominating state, and the formation of chromatin that is more permissive to subsequent rounds of transcription. Although speculative in nature, these proposed feedback mechanisms could, in the context of stochastic models of gene transcription (Lenstra et al., 2016), provide a localized form of chromatin-based epigenetic memory to ensure future rounds of gene transcription once the initial decision to transcribe has been made or to achieve new gene expression programs during developmental transitions. Although it is clear that more detailed mechanistic studies are required to examine the relevance of these proposed feedback mechanisms, they could provide a simple explanation for how genes transition into and maintain the H3K4me3-predominating state to sustain transcription.

## Experimental Procedures

### FRAP

FRAP experiments were performed on an UltraView spinning disk microscope (Perkin Elmer) equipped with an EM-CCD camera (Hamamatsu) using a 60x/1.4NA oil objective. 50 pre-bleach and 1,000 post-bleach images were captured at a rate of 8 frames per second (fps) (Figures 1B and C) after bleaching a circular diffraction limited spot of ∼2.5 μm diameter using a 488-nm laser line at 100% transmission. Alternatively, to capture the rapid recovery of the P/C/S^∗^ mutant effectively, we used an acquisition rate of 13 fps (Figures 1E and F). FRAP curves were calculated in MATLAB, normalizing for the initial conditions (brightness of the cell and brightness of the spot) and corrected for acquisition photobleaching over time (Mueller et al., 2012). Half recovery times (t_1/2_) were calculated using a biexponential fit. Briefly, this involved deriving t_1/2_ values from individual cells (Figures S1F and S1G) and then collecting the distribution of t_1/2_ values across biological triplicates for the same transgene (Figures S1H and S1I). To compare the dynamics of individual GFP-CFP1 transgenes, a Student’s t test was then used to calculate the probability (*p*) that there was no difference between the wild-type and mutant versions of CFP1.

### NMR Titrations of Histone Peptides

The ^1^H,^15^N HSQC spectra of 0.1–0.2 mM uniformly ^15^N-labeled CFP1 PHD finger in 20 mM Tris-HCl buffer, pH 6.8, 100 mM NaCl, 2.5 mM DTT, and 7% D_2_O were collected on a Varian INOVA 600 MHz spectrometer. Spectra were recorded at 298K using 1,024 × 128 increments, and a spectral width of 8,820 × 1,974 Hz in the ^1^H and ^15^N dimensions, respectively. The binding was characterized by monitoring chemical shift changes as histone tail peptides (synthesized by the University of Colorado Denver Peptide Core Facility) were added stepwise. The dissociation constants (K_d_s) were determined as described in the Supplemental Information.

### Fluorescence Spectroscopy

Spectra were recorded at 25°C on a Fluoromax-3 spectrofluorometer (HORIBA). The samples containing the CFP1 PHD finger in 20 mM Tris-HCl buffer, pH 6.8, 100 mM NaCl, and 2.5 mM DTT and progressively increasing concentrations of the histone peptide were excited at 280 nm. Emission spectra were recorded over a range of wavelengths between 320 and 380 nm, with a 1-nm step size and a 1-s integration time and averaged over 3 scans. The *K*_d_ values were determined as described in the Supplemental Information.

### ChIP and ChIP-Seq

ChIP was performed as described previously (Farcas et al., 2012), with minor modifications (see Supplemental Experimental Procedures). *S*equencing libraries were prepared with the NEBNext Ultra DNA Library Prep Kit for Illumina and sequenced on either an Illumina HiSeq2500 or NextSeq500.

### Calibrated Native ChIP-Seq

ChIP sequencing for H3K4me3 in ESCs was performed using a previously described calibrated native ChIP-seq approach (Rose et al., 2016), in which untreated or tamoxifen-treated *Cfp1*^*fl/fl*^ cells were spiked with a fixed number of *Drosophila* SG4 cells (see Supplemental Experimental Procedures). Libraries were prepared with NEBNext Ultra DNA Library Prep Kit for Illumina and quantified by qPCR using KAPA Illumina DNA standards as reference. Libraries were sequenced on an Illumina NextSeq500.

### 4sU RNA-Seq

Cells were treated with 500 μM 4-thiouridine (4sU) for 20 min and then RNA was isolated by TRIZOL (Thermo Fisher Scientific) extraction. RNA was incubated with Biotin-HPDP and biotinylated RNA was captured with μMACS streptavidin beads (Miltenyi). Biotinylated RNA was depleted of ribosomal RNAs using the Low Input RiboMinus Eukaryote System v2 kit (Thermo Fisher Scientific). cDNA libraries were prepared using the NEBNext Ultra Directional RNA Library Prep Kit and subjected to sequencing on the Illumina NextSeq500 platform.

### Quantitative Nuclear RNA-Seq

*Cfp1*^*fl/fl*^ ESCs were cultured for 96 hr in the presence or absence of 4-OHT. For each condition, 4 × 10^6^ cells were spiked with 1 × 10^6^ *Drosphila* SG4 cells. Nuclei were extracted, and an aliquot of nuclei corresponding to 4 × 10^5^ cells was collected for genomic DNA extraction, whereas the remaining nuclei were subject to conventional RNA extraction using TRIZOL (Thermo Fisher Scientific). Nuclear RNA was depleted of ribosomal RNAs using the NEBNext rRNA Depletion kit, and cDNA libraries were prepared using the NEBNext Ultra Directional RNA Library Prep Kit. In parallel, genomic DNA (gDNA) was used to prepare “input” DNA libraries using the NEBNext Ultra DNA Library Prep Kit. Nuclear RNA (nucRNA) and gDNA libraries were sequenced on the Illumina NextSeq500 platform.

## Author Contributions

D.A.B. (ORCID ID 0000-0001-7531-8230), V.D.C. (ORCID ID 0000-0002-2920-1274), A.F., J.A., S.I., L.S., T.G.K., H.K., and R.J.K. conceived the project. T.G.K., S.K., T.G.K., H.K., and R.J.K. secured funding. D.A.B., V.C.D., A.F., J.A., S.I., N.P.B., E.D., M.N., M.M., H.K.L. (ORCID ID 0000-0002-5694-0398), A.H.T., and H.W.K. (ORCID ID 0000-0001-5972-8926) generated reagents and performed the experiments. D.A.B., V.D.C., A.F., N.P.B., S.K., L.S., T.G.K., H.K., and R.J.K. wrote and edited the manuscript.

## Accession Numbers

The accession number for the datasets reported in this paper is GEO: GSE93538.

## Supplemental Information

Document S1. Supplemental Experimental Procedures and Figures S1–S6

Document S2. Article plus Supplemental Information
