# Supplementary material for: The SET1 Complex Selects Actively Transcribed Target Genes via Multivalent Interaction with CpG Island Chromatin
Source: Cell Rep. 2017 Sep 5;20(10):2313–27. doi: 10.1016/j.celrep.2017.08.030 (PMC5603731; doi:10.1016/j.celrep.2017.08.030)
Supplement: Document S1. Supplemental Experimental Procedures and Figures S1–S6 [file mmc1.pdf]

Cell Reports, Volume *20*

Supplemental Information

The SET1 Complex Selects Actively Transcribed Target Genes via Multivalent Interaction with CpG Island Chromatin

David A. Brown, Vincenzo Di Cerbo, Angelika Feldmann, Jaewoo Ahn, Shinsuke Ito, Neil P. Blackledge, Manabu Nakayama, Michael McClellan, Emilia Dimitrova, Anne H. Turberfield, Hannah K. Long, Hamish W. King, Skirmantas Kriaucionis, Lothar Schermelleh, Tatiana G. Kutateladze, Haruhiko Koseki, and Robert J. Klose

Supplementary Material

Supplementary Figures:

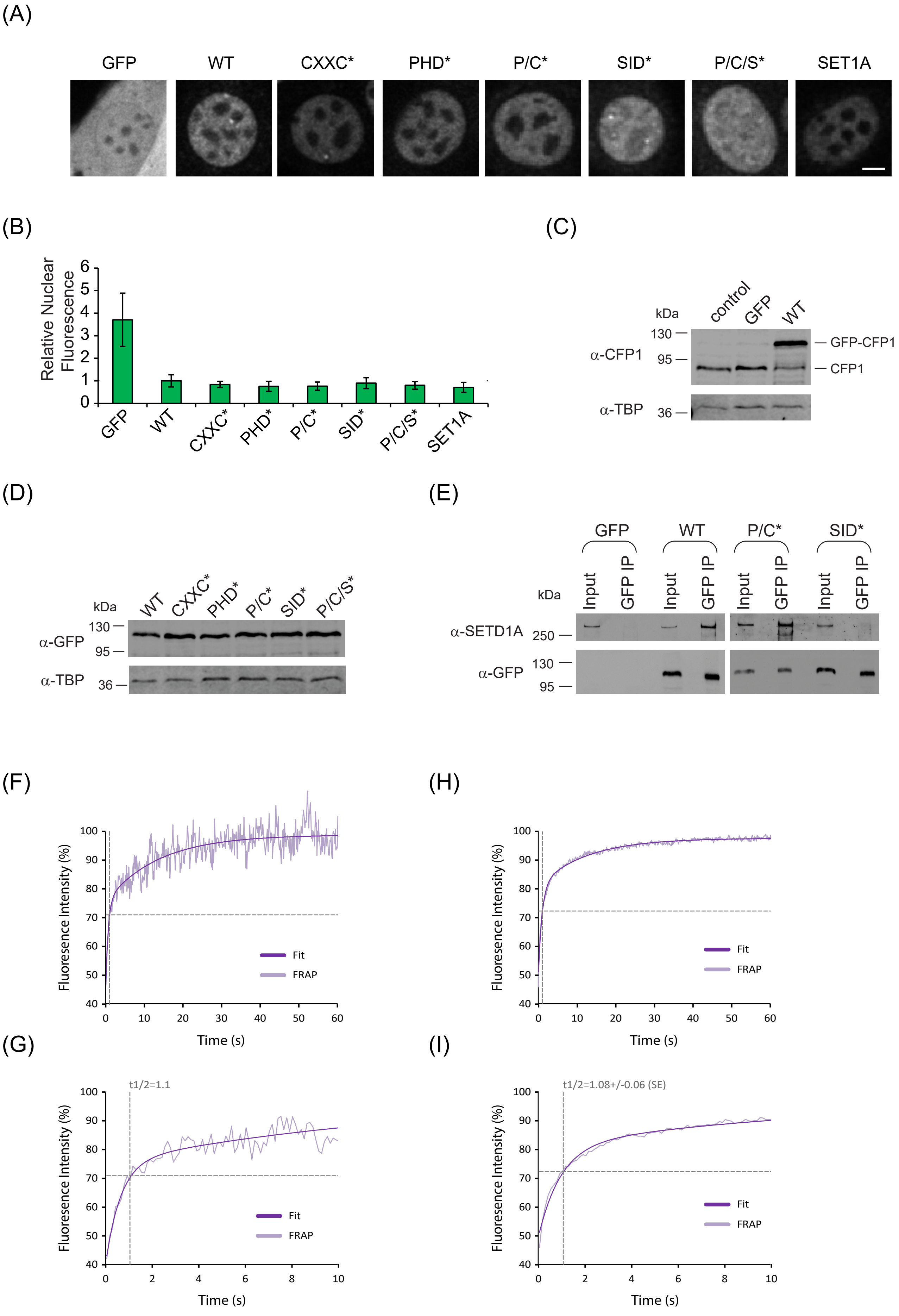
(A)
GFP
WT
CXXC*
PHD*
P/C*
SID*
P/C/S*
SET1A
(B)
Relative Nuclear Fluorescence
(C)
kDa
control
GFP
WT
α-CFP1
α-TBP
GFP-CFP1
CFP1
(D)
α-GFP
(E)
Input
GFP IP
α-SETD1A
(F)
(H)
(G)
(I)
Fluoresence Intensity (%)
Time (s)
Fit
FRAP
t1/2=1.1
t1/2=1.08+/-0.06 (SE)

Figure S1

Figure S1- Validation of transgene protein levels and complex formation in C127 cell lines and explanation of FRAP analysis approach used in this study. Related to Figure 1.

(A) Live cell images of stable GFP and GFP fusion protein expression in mouse C127 cells demonstrating nuclear localisation. The scale bar corresponds to 5 µm.
(B) Quantitation of GFP and GFP fusion protein fluorescence in stably expressing C127 cells indicating equivalent protein expression. Error bars correspond to the SEM from 3 biological replicates.
(C) Western blot analysis using a CFP1-specific antibody, indicating that GFP-CFP1 protein levels in WT cell line are comparable to endogenous CFP1 in both C127 control and GFP-only cells. Western blot analysis with a TATA box binding protein (TBP)-specific antibody was used to demonstrate equal loading of nuclear extracts.
(D) Western blot analysis using a GFP-specific antibody, indicating that GFP-CFP1 protein levels in WT cell line are highly similar to all mutant forms of GFP-CFP1 used in C127 FRAP and ChIP-seq experiments. Western blot analysis with a TATA box binding protein (TBP)-specific antibody was used to demonstrate equal loading of nuclear extracts.
(E) Immunoprecipitation with a GFP-specific antibody, using nuclear extract from GFP-only, GFP-CFP1 WT, GFP-CFP1 P/C* and GFP1-CFP1 SID* cell lines. Immunoprecipitated material was subjected to western blot analysis, with GFP- and SETD1A-specific antibodies. As expected, CFP1 WT co-immunoprecipitates with SETD1A. Importantly, mutating the PHD and CXXC domains of CFP1 does not affect the interaction with SETD1A, whereas mutating the SET1 interaction domain abolishes the SETD1A interaction.
(F) An example FRAP recording, and biexponential fit from an individual C127 cell stably expressing WT GFP-CFP1.
(G) The first 10 s of the recovery shown in (F), the estimate of $t_{1/2}$ is given above.
(H) Mean FRAP recovery and biexponential fit for WT GFP-CFP1 measured in 45 cells across three biological replicates.
(I) The first 10 s of the recovery shown in (H), the estimate of $t_{1/2}$ is given above.

(A)

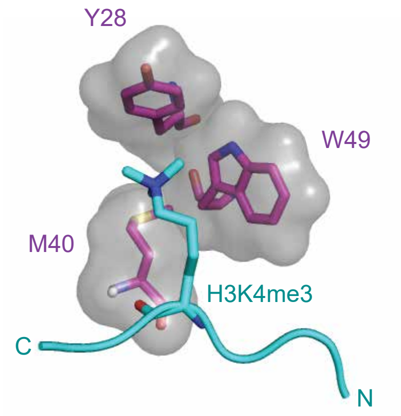

(B)

```
                                                               W49
                  *    *                         *      *       ↓   *  *                                     *  *
CFP1(28)       YCI--CRKPDIN-----------CFMIGCDN---CNE-WFHGDCIRITEKM------AKAIREWYCRECR
MLL1(1568)     FCPL-CDKCYDDDD-------YESKMMQCGK---CDR-WVHSKCENLSDEMYEILSNLPESVAYTCVNCT
BPTF(2743)     YCI--CKTPYD-----------ESKFYIGCDR---C-QNWYHGRCVGILQSEAELI------DEYVCPQCQ
RAG2(418)      TCCPTCDVDINTWVPFYSTELNKPAMIYCSHGDGH---WVHAQCMDLAERTLIHLSAGS--NKYYCNEHV
TAF3(867)      ICPG-CNKPDDG-----------SPMIGCDD---CDD-WYHWPCVGIMT-------APPEEMQWFCPKCA
ING2(216)      YCL--CNQVSYG------------EMIGCDNEQ-CPIEWFHFSCVSLTYKP---------KGKWYCPKCR
```

(C)

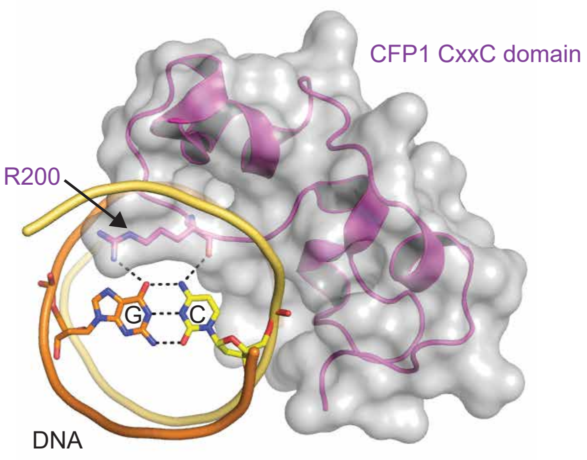

(D)

```
                                                    R200
                     *  *  *     *  *  *              ↓  *    *
CFP1(163)      KRSARMCGECEACRRTEDCGHCDFCRDMKKFGGPNKIRQKCRLRQCQLRARE
KDM2A(565)     RRRRVRCRKCKACVQGE-CGVCHYCRDMKKFGGPGRMKQSCVLRQCLAPRLP
KDM2B(607)     RRRRTRCRKCEACLRTE-CGECHFCKDMKKFGGPGRMKQSCIMRQCIAPVLP
FBXL19(33)     RRRRTRCRRCRACVRTE-CGDCHFCRDMKKFGGPGRMKQSCLLRQCTAPVLP
DNMT1(663)     AFKRRRCGVCEVCQQPE-CGKCKACKDMVKFGGSGRSKQACQERRCPNMAMK
MLL1(1149)     GRRSRRCGQCPGCQVPEDCGVCTNCLDKPKFGGRNIKKQCCKMRKCQNLQWM
MLL2(960)      KMRMARCGHCRGCLRVQDCGSCVNCLDKPKFGGPNTKKQCCVYRKCDKIEAR
```

(E)

```
                                                K389 Y390
                            *     *                ↓ ↓ *   *
Hs CFP1(366)     ERADAKDPASLPQCLGPGCVRPAQPSSKYCSDDCGMKLA
Mm CFP1(366)     ERADAKDPASLPQCLGPGCVRAAQPSSKYCSDDCGMKLA
Xl CFP1(282)     ERMETRDFSSLRQCLGPSCVQPARANSKYCSDDCGMKLA
Dr CFP1(269)     DRTDGRHGGDTQQCLGPNCIEPARPNSKYCSEDCGMKLA
Dm CFP1(260)     EIFLNPELQGIRQCYGPNCCSHARPQSKYCSDKCGFNLA
Sc Spp1(84)      GEGSLPKTLWKRKCRISDCYKPCLQDSKYCSEEHGREFV
```

Figure S2

Figure S2- Rational design of CFP1 mutations. Related to Figure 1.

(A) To identify residues that would abrogate the association of the CFP1 PHD domain with methylated lysine ligands we built a structural model of the human CFP1 PHD aromatic cage based on the crystal structure of the BPTF PHD domain in complex with an H3K4me3 peptide (PDB ID - 2FUU). This model predicts that the peptide (cyan) would project its methyl-lysine residue into an aromatic cage comprised of residues Y28, M40 and W49 of CFP1. We chose to substitute W49 with alanine to create the PHD* mutant as this is predicted to inhibit methyl-lysine binding but not result in unfolding of the PHD domain (Li et al., 2006, Pena et al., 2006, Ramon-Maiques et al., 2007)
(B) Multiple sequence alignment of the PHD domains of human CFP1 (NP_001095124.1) and the known H3K4me3 binding PHD domains of MLL1 (NP_001184033.1), BPTF (NP_872579.2), RAG2 (NP_000527.2), TAF3 (NP_114129.1), and ING2 (NP_075992.2). The numbers in brackets next to each sequence indicate the starting residue number within the intact protein that corresponds to the first residue of the alignment. Asterisks mark zinc coordinating residues that are required for domain structure. The position of W49 in CFP1 is indicated by a blue arrow.
(C) The published crystal structure (PDB 3QMG) of the human CFP1 CXXC domain bound to DNA containing a non-methylated CpG dinucleotide (Xu et al., 2011) illustrates recognition of the C-G base pair by arginine 200 in CFP1 (R200). We chose to substitute R200 with alanine to create the CXXC* mutant as this substitution is predicted to inhibit binding to non-methylated CpG but not to result in an unfolded CXXC domain (Allen et al., 2006, Blackledge et al., 2010, Cierpicki et al., 2010, Zhou et al., 2012).
(D) Multiple sequence alignment of human nonmethyl-CpG-binding CXXC domains from CFP1 (NP_001095124.1), KDM2A (NP_036440.1), KDM2B (NP_115979.3), FBXL19 (NP_001093254.2), DNMT1 (NP_001124295.1), and MLL1 (NP_001184033.1), MLL2 (NP_055542.1). The numbers in brackets next to each sequence indicate the starting residue number within the intact protein that corresponds to the first residue of the alignment. Asterisks mark zinc coordinating residues that are required for domain structure, and the position of CFP1 R200 is indicated with a yellow arrow.
(E) Multiple sequence alignment of CFP1 SET1 interactions domains (SID) from *Homo sapiens* (NP_001095124.1), *Mus musculus* (NP_083144.1), *Xenopus laevis* (NP_001085408.1), *Danio rerio* (NP_956627.1), *Drosophila melanogaster* (NP_572556.1) and the *Saccharomyces cerevisiae* homologue Spp1 (ONH80964.1). The numbers in brackets next to each sequence indicate the starting residue number within the intact protein that corresponds to the first residue of the alignment. Cysteines in this domain (marked by asterisk) are predicted to create a structural zinc finger like fold to support interaction with SET1A. To create a SID mutant that would not affect the structure of CFP1 but should inhibit binding to SET1A we chose to substitute the highly conserved residues K389 and Y390 in CFP1 (Pink arrows) with alanine.

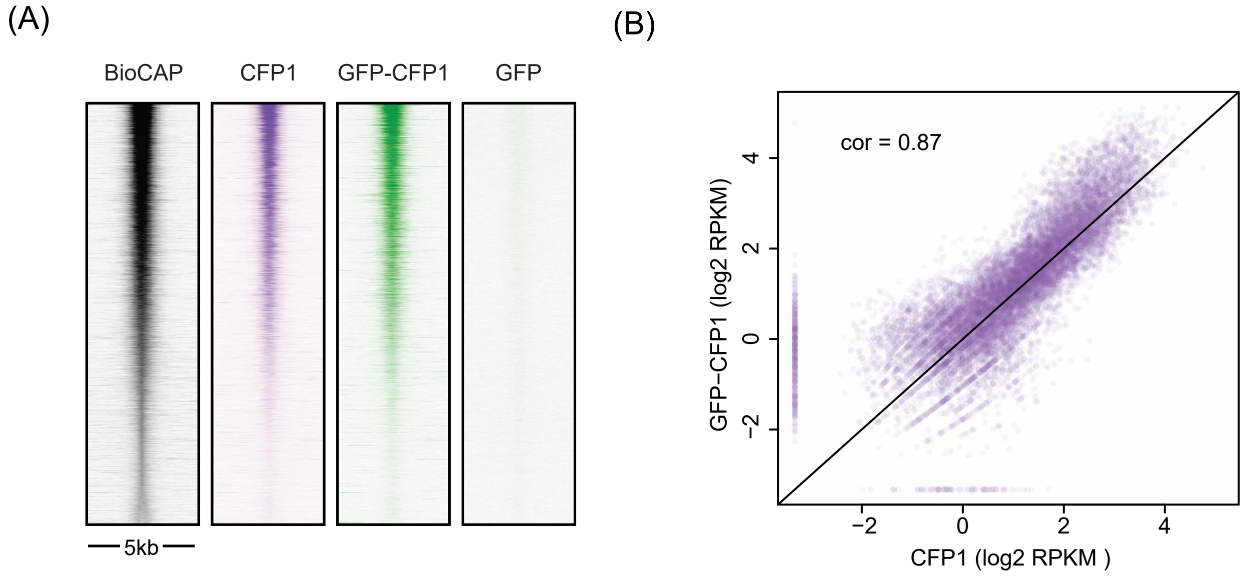

(A)
BioCAP
CFP1
GFP-CFP1
GFP
5kb
(B)
cor = 0.87
GFP−CFP1 (log2 RPKM)
CFP1 (log2 RPKM)
−2
0
2
4

Figure S3

Figure S3- GFP-CFP1 recapitulates endogenous CFP1 occupancy on chromatin. Related to Figure 2.

(A) A heatmap of CFP1, GFP-CFP1, and GFP ChIP-seq signal over all NMIs ranked by Bio-CAP intensity.
(B) A scatterplot of GFP-CFP1 and CFP1 ChIP-seq signal at NMIs. The ChIP-seq signal for GFP-CFP1 and endogenous CFP1 are highly correlated (R=0.87, Spearman Rank correlation) indicating that GFP-CFP1 recapitulates endogenous CFP1 occupancy on chromatin.

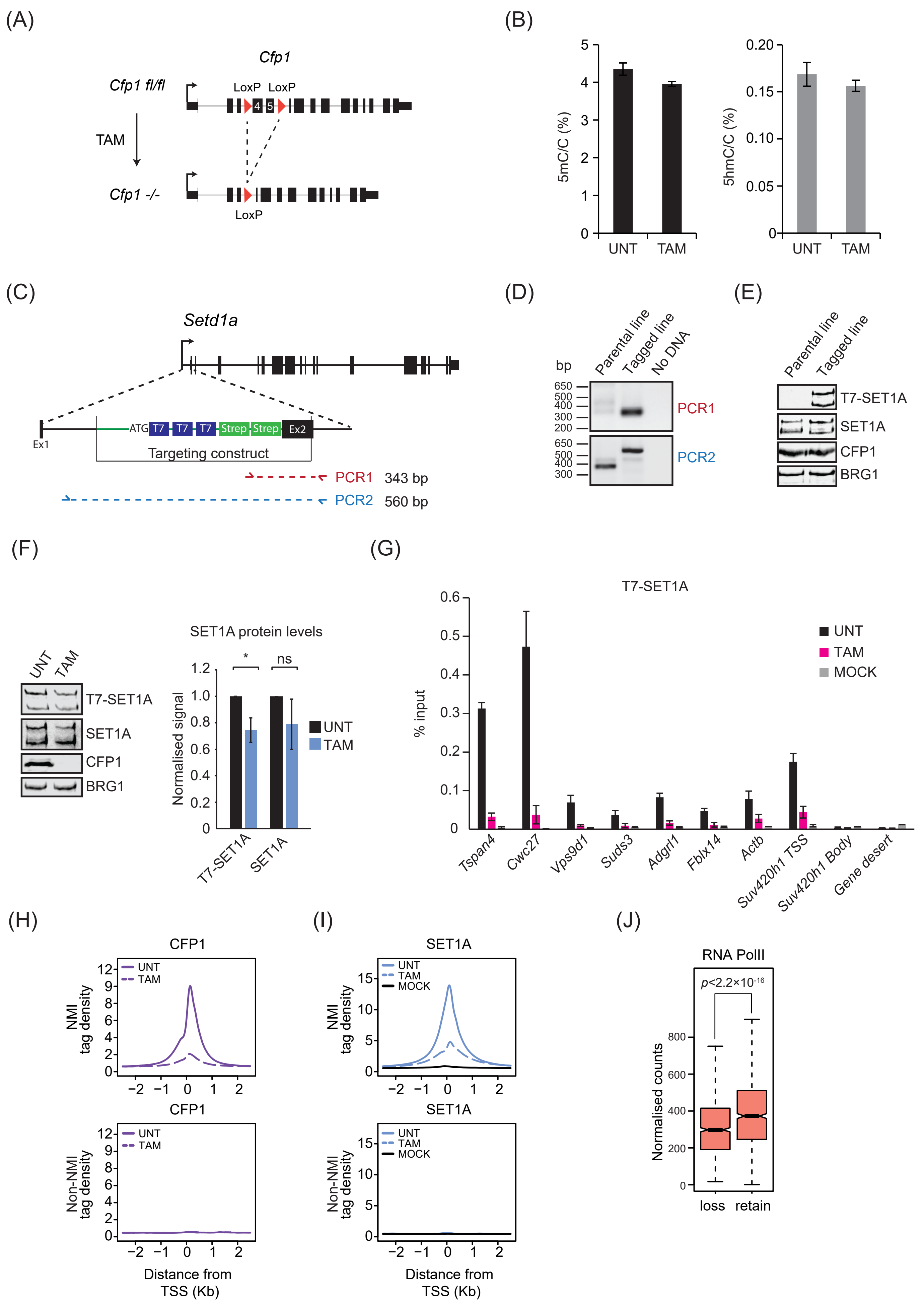

(A)
Cfp1
Cfp1 fl/fl
LoxP LoxP
4 5
TAM
Cfp1 -/-
LoxP
(B)
5mC/C (%)
5hmC/C (%)
UNT TAM
(C)
Setd1a
Ex1
ATG T7 T7 T7 Strep Strep Ex2
Targeting construct
PCR1 343 bp
PCR2 560 bp
(D)
bp
Parental line
Tagged line
No DNA
PCR1
PCR2
(E)
Parental line
Tagged line
T7-SET1A
SET1A
CFP1
BRG1
(F)
UNT TAM
T7-SET1A
SET1A
CFP1
BRG1
SET1A protein levels
Normalised signal
*
ns
UNT
TAM
T7-SET1A
SET1A
(G)
T7-SET1A
% input
UNT
TAM
MOCK
Tspan4
Cwc27
Vps9d1
Suds3
Adgrl1
Fblx14
Actb
Suv420h1 TSS
Suv420h1 Body
Gene desert
(H)
CFP1
NMI tag density
Non-NMI tag density
UNT
TAM
Distance from TSS (Kb)
(I)
SET1A
NMI tag density
Non-NMI tag density
UNT
TAM
MOCK
Distance from TSS (Kb)
(J)
RNA PolII
p<2.2×10-16
Normalised counts
loss
retain

Figure S4

Figure S4- CFP1 is the central determinant in SET1A occupancy on chromatin. Related to Figure 4.

(A) A schematic illustrating the location of the engineered loxP sites in the *Cfp1* gene.
(B) Graphs indicating the percentage of cystosine that is methylated (5mC) and hydroxymethylated (5hmC) in untreated (UNT) and tamoxifen treated (TAM) *Cfp1*$^{fl/fl}$ ESCs, as determined by HPLC. Error bars correspond to the SEM for 3 biological replicates. There are no significant alterations in 5mC or 5hmC following removal of CFP1.
(C) A schematic illustrating how the triple T7 double StrepII tag was knocked into the endogenous *Setd1a* gene in the *Cfp1*$^{fl/fl}$ ESCs. The PCR primers used to screen for homologous recombination are indicated.
(D) A genomic PCR verifying homozygous epitope tagging of the endogenous *Setd1a* gene.
(E) Western blot analysis of SET1A in the parental and epitope tagged cell line with epitope tag specific T7 antibody (upper panel) and SET1A specific antibodies (second panel from the top).
(F) Western blot analysis of SET1A in untreated (UNT) or tamoxifen treated (TAM) *Cfp1*$^{fl/fl}$ ESCs using epitope tag and SET1A-specific antibodies (left panel). The levels of SET1A under these conditions were analysed in at least biological triplicate and quantified (right panel) with error bars corresponding to the SD. This illustrates that loss of CFP1 leads to only a minor reduction in SET1A protein levels (* represents a student's t-test $p \leq 0.05$).
(G) ChIP-qPCR analysis of epitope tagged SET1A protein occupancy at a series of target gene promoters in untreated (UNT) and tamoxifen treated (TAM) *Cfp1*$^{fl/fl}$ ESCs with T7-tagged SET1A, and in the untagged *Cfp1*$^{fl/fl}$ cell line (MOCK). Error bars represent the SD from at least 3 biological replicates. This demonstrates the loss of SET1A binding following removal of CFP1 in agreement with ChIP-seq analysis.
(H) Metaplot analysis of CFP1 ChIP-seq at NMI (upper panel) and non-NMI sites (lower panel) in untreated (UNT, solid line) and tamoxifen treated (TAM, dotted line) *Cfp1*$^{fl/fl}$ ESCs.
(I) Metaplot analysis of SET1A ChIP-seq at NMI (upper panel) and non-NMI sites (lower panel) in untreated (UNT, solid line) and tamoxifen treated (TAM, dotted line) *Cfp1*$^{fl/fl}$ ESCs.
(J) Box plot illustrating RNA PolII ChIP-seq signal (8WG16, (Brookes et al., 2012)) at the top 10% of genes that lose or retain SET1A. This illustrates that genes that retain the most SET1A have higher levels of RNA PolII than those that lose the most SET1A. The *p* value denotes statistical significance calculated by a Wilcoxon signed rank test.

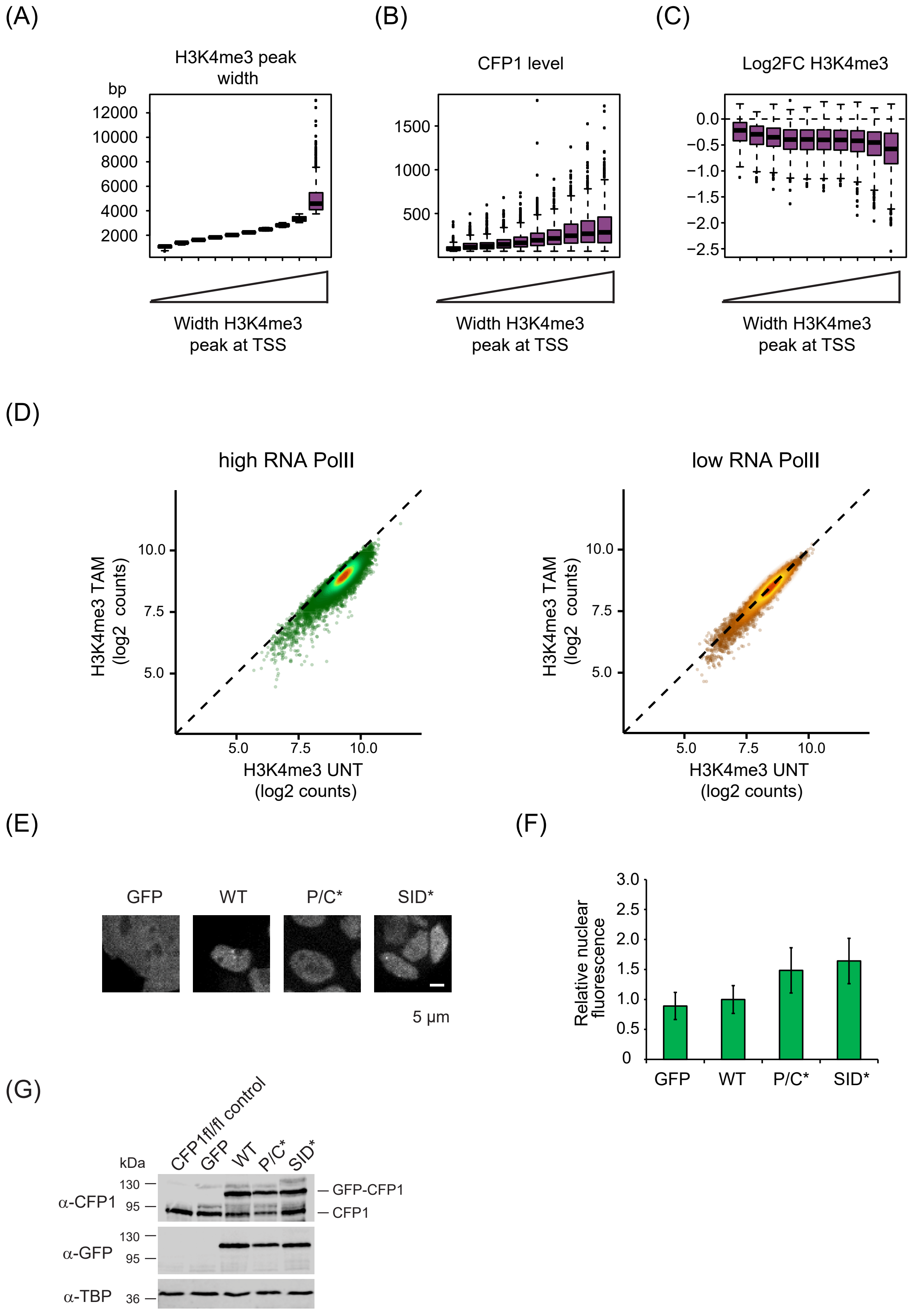
(A)
H3K4me3 peak width
bp
12000
10000
8000
6000
4000
2000
Width H3K4me3 peak at TSS
(B)
CFP1 level
1500
1000
500
Width H3K4me3 peak at TSS
(C)
Log2FC H3K4me3
0.0
−0.5
−1.0
−1.5
−2.0
−2.5
Width H3K4me3 peak at TSS
(D)
high RNA PolII
H3K4me3 TAM (log2 counts)
10.0
7.5
5.0
H3K4me3 UNT (log2 counts)
low RNA PolII
H3K4me3 TAM (log2 counts)
H3K4me3 UNT (log2 counts)
(E)
GFP
WT
P/C*
SID*
5 µm
(F)
Relative nuclear fluorescence
3.0
2.5
2.0
1.5
1.0
0.5
0
GFP
WT
P/C*
SID*
(G)
kDa
CFP1fl/fl control
GFP
WT
P/C*
SID*
α-CFP1
130
95
GFP-CFP1
CFP1
α-GFP
130
95
α-TBP
36

Figure S5

Figure S5- Broad peaks of H3K4me3 are most affected by loss of CFP1. Related to Figure 5.

(A-C) Non-divergent CFP1+ NMI associated genes were binned equally based on the width of their H3K4me3 peak as illustrated in (A). CFP1 levels and log2 fold change in H3K4me3 were then box plotted within the same bins (B and C). This revealed that the most prominent reduction in H3K4me3 following CFP1 loss occurred at genes with higher levels of CFP1 and that had wider peaks of H3K4me3.

(D) A scatterplot illustrating that genes with high RNA PolII occupancy (left panel) but not those with low RNA PolII occupancy (right panel) lose H3K4me3 at their TSS following tamoxifen treatment. The scatterplots correspond to non-divergent genes with an H3K4me3 peak overlapping their TSS and a gene was considered to have high RNA PolII if it had more than 2^6.2 PolII 8WG16 (Brookes et al., 2012) counts at the TSS.

(E) Live cell images of stable GFP and GFP fusion protein expression in mouse ESCs demonstrating nuclear localisation. The scale bar corresponds to 5 µm.

(F) Quantitation of GFP and GFP fusion protein fluorescence in stably expressing mouse ESCs, indicating equivalent protein expression. Error bars correspond to the SEM from 3 biological replicates.

(G) Western blot analysis using CFP1- and GFP-specific antibodies, indicating that GFP-CFP1 protein levels in *Cfp1*$^{fl/fl}$ ESC rescue lines (GFP-CFP1 WT, P/C* and SID*) are very similar endogenous CFP1 levels in both *Cfp1*$^{fl/fl}$ and GFP-only control cells. Western blot analysis with a TATA box binding protein (TBP)-specific antibody was used to demonstrate equal loading of nuclear extracts.

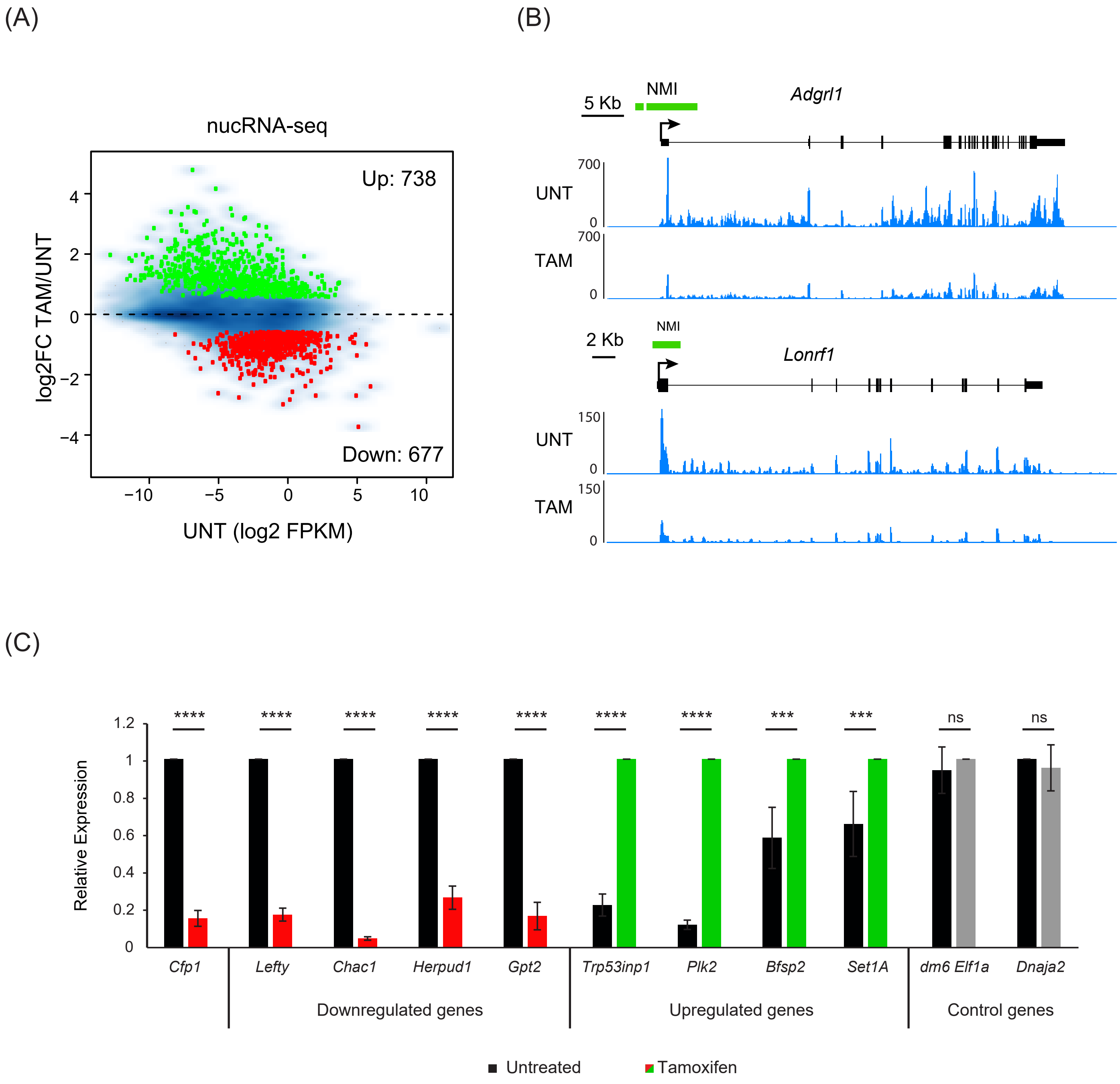
(A)
nucRNA-seq
Up: 738
Down: 677
log2FC TAM/UNT
UNT (log2 FPKM)
(B)
5 Kb
NMI
Adgrl1
UNT
TAM
2 Kb
NMI
Lonrf1
UNT
TAM
(C)
Relative Expression
Cfp1
Lefty
Chac1
Herpud1
Gpt2
Trp53inp1
Plk2
Bfsp2
Set1A
dm6 Elf1a
Dnaja2
Downregulated genes
Upregulated genes
Control genes
Untreated
Tamoxifen

Figure S6

Figure S6- Loss of CFP1 leads to widespread effects on gene expression. Related to Figure 6.

(A) An MA plot showing log2 fold change in nuclear RNA-seq signal of non-CFP1 bound genes in untreated (UNT) and tamoxifen treated (TAM) *Cfp1*$^{fl/fl}$ ESCs. Red and green points depict significantly down- (677) and upregulated genes (738) that change in expression by more than 1.5-fold.
(B) Genomic snapshots showing examples of two CFP1-bound NMI genes that show reductions in gene expression following loss of CFP1.
(C) Quantitative RT-qPCR validating gene expression changes observed by nuclear-RNA-seq at series of misregulated genes (**** represents a student's t-test $p \leq 0.0001$ and *** $p \leq 0.001$). Grey bars correspond to control genes which do not change expression in mouse (*Dnaja*) or the *Drosophila* (*Elf1a*) calibration sample following tamoxifen treatment.

Supplementary Experimental Procedures:

Cell culture

Mouse C127 cells were grown at 37°C and 5% CO_2 in Dulbecco's Modified Eagle Medium (DMEM) supplemented with 10% fetal bovine serum (Biosera) and 1x penicillin-streptomycin solution (Gibco). Embryonic stem cells were grown on gelatin-coated dishes in DMEM supplemented with 15% fetal bovine serum (Biosera), 1x MEM non-essential amino acids (Gibco), 2 mM L-glutamine (Gibco), 1x penicillin-streptomycin solution (Gibco), 0.5 mM b-mercaptoethanol (Gibco), and leukemia inhibitory factor. *Drosophila* S2 (SG4) cells were grown adhesively at 25°C in Schneider Drosophila Medium (Gibco), supplemented with 1x penicillin-streptomycin solution and 10% fetal bovine serum (Biosera) that was previously heat-inactivated at 60°C for 30 min.

Stable transgene expression

cDNAs were inserted into a modified pCAG-IRES-puro expression vector containing an N-terminal GFP tag by ligation independent cloning. To generate C127 cell lines expressing GFP-fusions, expression vectors were transfected using Fugene HD (Promega). Following transfection, cells were selected with 2.5 µg/ml puromycin until individual colonies formed and clonal isolates for individual GFP-fusions were selected that displayed equal transgene expression. To generate cell lines expressing GFP-CFP1 in CFP1 conditional mouse embryonic stem cells, *$Cfp1^{fl/fl}$:Rosa26-ERT2CRE* cells were transfected with the appropriate expression vector using Lipofectamine 2000 (Invitrogen) and selected with 1 µg/ml puromycin until individual colonies formed. Clonal isolates for individual GFP-fusions were selected that displayed equal transgene expression.

CRISPR/Cas9-mediated knock-in

To insert the 3xT7-2xStrepII tag into the *Setd1a* gene we employed CRISPR/Cas9 aided gene targeting as reported in (Ran et al., 2013). A guide RNA (gRNA) was identified (http://crispr.mit.edu/) which overlapped with the region proximal to the ATG of the *Setd1a* gene. A gene targeting construct was generated by PCR amplification that had the 3xT7-2xStrepII tag flanked by roughly 160 bp homology arms to act as repair template for SET1A tagging (Figure S4). *$Cfp1^{fl/fl}$* ESCs were transiently co-transfected with the Cas9 plasmid containing the sgRNA sequence and with the targeting construct (Lipofectamine 3000, Invitrogen). After 24h, 0.5 µg/mL puromycin was added for 48 h to enrich for transfected cells. Puromycin selected cells were and then plated at limiting dilution without puromycin and individual clones were allowed to form. Individual clones were then screened by western blot and homozygote tagging validated by PCR on genomic DNA.

Fluorescence recovery after photobleaching (FRAP)

35 mm µdishes (Ibidi) were seeded with 60,000 C127 cells and imaged after 24 h. Imaging was performed in phenol red-free DMEM (Lonza) containing 10% FCS, 12.5 mM HEPES (Gibco) and 40 µM sodium pyruvate (Gibco), and maintained at 37°C and 5% CO_2 in a humidified incubation chamber (Tokai Hit). FRAP experiments were performed on an UltraView spinning disk microscope (Perkin Elmer) equipped with and EM-CCD camera (Hamamatsu) using a 60x/1.4NA oil objective. 50 pre-

bleach and 1000 post-bleach images were captured at a rate of 8 fps (Figure 1B and C) after bleaching a circular diffraction limited spot of ~2.5 µm diameter using 488nm laser line at 100% transmission. Alternatively, to capture the rapid recover of the P/C/S* mutant effectively we used an acquisition rate of 13 fps in Figure 1E and F. FRAP curves were calculated in MATLAB, normalizing for the initial conditions (brightness of the cell and brightness of the spot) and corrected for acquisition photobleaching over time (Mueller et al., 2012). Half recovery times ($t_{1/2}$) were calculated using a biexponential fit. Briefly, this involved deriving $t_{1/2}$ values from individual cells (Figure S1F and G) and then collecting the distribution of $t_{1/2}$ values across biological triplicates for the same transgene (Figure S1H and I). To compare the dynamics of individual GFP-CFP1 transgenes, a student's t-test was then used to calculate the probability (p) that there was no difference between the wild-type and mutant versions of CFP1.

Antibodies

A CFP1 antibody was generated by immunizing a rabbit (PTU/BS Scottish National Blood Transfusion Service) with a 6xHis tagged protein antigen encoding amino acids 206 to 360 of human CFP1. α-CFP1 was then affinity purified against the same antigen immobilised on an Affigel10 resin as described previously (Farcas et al., 2012). The source and use of other antibodies is indicated in the table below.

Antibody	Type	Source	USE
α-CFP1f	Rabbit polyclonal	This Study	ChIP and Western
α-KDM2B	Rabbit polyclonal	(Farcas et al., 2012)	ChIP and Western
α-GFP	Mouse monoclonal	Invitrogen (3E6)	ChIP
α-Pol II CTD	Mouse monoclonal	Covance (8WG16)	ChIP and Western
α-T7-Tag XP®	Rabbit monoclonal	Cell Signalling (D9E1X)	ChIP and Western
α-SET1A	Rabbit polyclonal	Bethyl (A300-289A)	Western
α-BRG1	Rabbit monoclonal	Abcam (ab110641)	Western
α-H3K4me3	Rabbit polyclonal	(Farcas et al., 2012)	ChIP and Western
α-H3K4me2	Rabbit monoclonal	Abcam (Y47 - ab32365)	Western
α-H3K4me1	Rabbit polyclonal	Abcam (ab8895)	Western
α-H2A	Mouse monclonal	Cell Signalling (L88A6)	Western

Recombinant protein expression

The CFP1 PHD finger construct (residues 23 – 105) was cloned into the pGEX-6P-1 (GE Healthcare) expression vector with ampicillin resistance. Protein was expressed in *E. coli* BL21 (DE3) RIL cells grown in either Luria Broth or $^{15}NH_4Cl$ minimal media, supplemented with 60 µM $ZnCl_2$. After induction with IPTG (0.5 mM) for 16 h at 18°C, cells were harvested and lysed by sonication. GST-fusion proteins were purified on glutathione Sepharose 4B beads (GE Healthcare). The GST tag was cleaved with PreScission protease (Amersham). When necessary, the proteins were further purified by size exclusion chromatography over a HiPrep 16/60 Sephacryl S-100 column (GE Healthcare) and concentrated in Millipore concentrators (Millipore).

Protein extraction and western blot

Cfp1$^{fl/fl}$ ESCs were harvested and washed in PBS. For histone extraction, pellets were resuspended in TEB buffer (0.5% Triton X-100 in PBS, supplemented with 1x Complete EDTA-free inhibitor cocktail, 1 mM 4-(2-Aminoethyl)benzenesulfonyl fluoride hydrochloride [AEBSF]) and rotated for 10 min at 4°C. Nuclei were then collected by centrifugation for 10 min at 6500 g and histones were extracted with 0.2 N HCl during overnight rotation at 4°C. Protein concentration was measured by Bradford assay and equal amounts of histone were separated on an 18.7% SDS-polyacrylamide gel and subjected to western blot analysis.
For nuclear extract, cells were resuspended in 10 volumes of Buffer A (10 mM HEPES pH 7.9, 1.5 mM $MgCl_2$, 10 mM KCl, 0.5 mM-DTT, 1x Complete EDTA-free inhibitor cocktail, 1 mM AEBSF) and incubated 10 min on ice. Recovered pellets were then resuspended in 3 volumes of Buffer A supplemented with 0.1% NP40 and inverted 10 times. Resulting nuclei were recovered by centrifugation and were resuspended in Buffer C (5 mM HEPES pH 7.9, 26% glycerol, 1.5 mM $MgCl_2$, 0.2 mM EDTA, 0.5 mM DTT, 400 mM NaCl, supplemented with 1x Complete EDTA-free inhibitor cocktail, 1 mM AEBSF) and incubated for 1 h on ice, followed by centrifugation and recovery of the soluble fraction. Protein concentration was measured by Bradford assay and equal amounts of protein were used for SDS-PAGE and western blotting. To quantify western blot signals secondary antibodies conjugated with infrared dyes (IRDye 800CW goat anti-rabbit or IRDye 680RD goat anti-mouse, LI-COR) and a LI-COR FC instrument were used.

NMR titrations of histone peptides

The $^{1}H,^{15}N$ HSQC spectra of 0.1 – 0.2 mM uniformly ^{15}N-labeled CFP1 PHD finger in 20 mM Tris-HCl buffer pH 6.8, 100 mM NaCl, 2.5 mM DTT, and 7% D_2O were collected on a Varian INOVA 600 MHz spectrometer. The spectra were recorded at 298K using 1024 × 128 increments, and a spectral width of 8820 × 1974 Hz in the ^{1}H and ^{15}N dimensions, respectively. The binding was characterized by monitoring chemical shift changes as histone tail peptides (synthesized by the University of Colorado Denver Peptide Core Facility) were added stepwise. The dissociation constants (K_ds) were determined using a nonlinear least-squares analysis in KaleidaGraph and the equation:

$$\Delta\delta = \Delta\delta_{\max}\left(\left([L]+[P]+K_d\right)-\sqrt{\left([L]+[P]+K_d\right)^2-4[P][L]}\right)\Big/2[P]$$

where [L] is concentration of the peptide, [P] is concentration of the protein, Δδ is the observed chemical shift change, and $\Delta\delta_{max}$ is the normalized chemical shift change at saturation. Normalized chemical shift changes were calculated using the equation

$$\Delta\delta=\sqrt{(\Delta\delta H)^2+(\Delta\delta N/5)^2}$$

where Δδ is the change in chemical shift in parts per million (ppm).

Fluorescence spectroscopy

Spectra were recorded at 25˚C on a Fluoromax-3 spectrofluorometer (HORIBA). The samples containing the CFP1 PHD finger in 20 mM Tris-HCl buffer pH 6.8, 100 mM NaCl, 2.5 mM DTT and progressively increasing concentrations of the histone peptide were excited at 280 nm. Emission spectra were recorded over a range of wavelengths between 320 and 380 nm with a 1 nm step size

and a 1 s integration time and averaged over 3 scans. The K_d values were determined using a nonlinear least-squares analysis and the equation:

$$\Delta I = \Delta I_{\max}\left(\left([L]+[P]+K_d\right)-\sqrt{\left([L]+[P]+K_d\right)^2-4[P][L]}\right)\Big/2[P]$$

where [L] is the concentration of the histone peptide, [P] is the concentration of CFP1 PHD finger, ΔI is the observed change of signal intensity, and ΔI_{max} is the difference in signal intensity of the free and bound states of the PHD finger. The K_d value was averaged over three separate experiments, with error calculated as the standard deviation between the runs.

HPLC

Genomic DNA was treated with 1U RNase A (Thermo Scientific) per 10 µg, purified by phenol chloroform ethanol precipitation and incubated overnight in hydrolysis solution (45 mM NaCl, 9 mM $MgCl_2$, 9 mM Tris-HCl pH 7.9, ≥ 250 U/ml Benzonase (Sigma), 50 mU/ml Phosphodiesterase I, ≥ 20 U/ml Alkaline phosphatase, 46.8 ng/ml EHNA hydrochloride, 8.64 µM deferoxamine). Protein components were removed by centrifugation through Amicon centrifugal filter unit (3 kDa cut-off, Millipore) before samples were lyophilised and resuspended in buffer A. Nucleosides were resolved with an Agilent UHPLC 1290 instrument fitted with Eclipse Plus C18 RRHD 1.8 µm (2.1 × 150 mm column) and detected and quantified with Agilent 1290 DAD fitted with a Max-Light 60 mm cell. Buffer A was 100 mM ammonium acetate pH 6.5, buffer B was 40% acetonitrile and the flow rate 0.4 ml min^{-1}. The gradient was between 1.8–100% of 40% acetonitrile with the following steps: 1–2 min, 100% A; 2–16 min 98.2% A, 1.8% B; 16–18 min 70% A, 30% B; 18–20 min 50% A, 50% B; 20–21.5 min 25% A, 75% B; 21.5–22.5 min 100% B; 22.5-24.5 min 100% A. Relative abundance of 5mC and 5hmC were established by detection of adenosine at 280nm allowing determination of total cytosine by extinction coefficient calculation using standards.

Bio-CAP sequencing

Bio-CAP sequencing was performed on genomic DNA isolated from C127 cells as described in (Blackledge et al., 2012, Long et al., 2013).

ATAC-seq

Chromatin accessibility was assayed using an adaptation of the assay for transposase accessible-chromatin (ATAC)-seq (Buenrostro et al., 2013). Briefly, 5×10^6 cells were harvested, washed with PBS and nuclei were isolated using 1 mL HS Lysis buffer (50 mM KCl, 10 mM $MgSO_4 \cdot 7H_20$, 5 mM HEPES, 0.05 % NP40, 1 mM PMSF, 3 mM DTT) for 1 min at room temperature. Nuclei were centrifuged at 1000 g for 5 min at 4°C, followed by a total of three washes with ice-cold RSB buffer (10 mM NaCl, 10 mM Tris-HCl pH 7.4, 3 mM $MgCl_2$), to remove as much of contaminating cytoplasmic and mitochondrial material as possible. Nuclei were then counted, and 5×10^4 nuclei were resuspended in Tn5 reaction buffer (10 mM TAPS, 5 mM $MgCl_2$, 10% dimethylformamide) and 2 µl of Tn5 transposase (25 µM) made in house as previously described (Picelli et al., 2014). Nuclei were then incubated for 30 min at 37°C, before isolation and purification of tagmented DNA using QiaQuick MinElute columns (Qiagen). To control for sequence bias of the Tn5 transposase, an ATAC "input" sample was generated, by tagmenting genomic DNA from ESCs with Tn5 for 30 min at 55°C. ATAC-seq libraries were prepared by PCR amplification using custom made Illumina barcodes previously described (Buenrostro et al., 2013)

and the NEBNext® High-Fidelity 2X PCR Master Mix with 8-10 cycles. Libraries were purified with two rounds of AMPure XP bead cleanup (1.5X beads:sample), followed by quantification by qPCR using SensiMix SYBR (Bioline) and KAPA Library Quantification DNA standards (KAPA Biosystems). ATAC-seq libraries were sequenced on Illumina NextSeq500 using 80 bp paired-end reads in biological triplicate.

ChIP and ChIP-sequencing

For C127 cells, H3K4me3 ChIP was performed by crosslinking cells in PBS with 1% formaldehyde for 15 min at 25˚C and was quenched with 150 mM glycine. For CFP1, KDM2B and RNAPII ChIP-seq, cells were fixed with 2 µM EGS for 1 h prior to the formaldehyde crosslinking. Crosslinked cells were incubated in lysis buffer (50 mM HEPES pH 7.9, 140 mM NaCl, 1 mM EDTA, 10% glycerol, 0.5% NP40, 0.25% TritonX-100) for 10 min at 4˚C. The released nuclei were then washed (10 mM Tris-HCl, pH 8.0, 200 mM NaCl, 1 mM EDTA, 0.5 mM EGTA) for 10 min at 4˚C. Chromatin was resuspended (10 mM Tris-HCl pH 8.0, 100 mM NaCl, 1 mM EDTA, 0.5 mM EGTA, 0.1% Na deoxycholate, 0.5% N-lauroylsarcosine) and sonicated for 50 min using a BioRuptor sonicator (Diagenode), shearing genomic DNA into 0.5–1 kb fragments. After sonication, TritonX-100 was added to a final concentration of 1.5%. Following centrifugation at 19000 g for 10 min at 4˚C, the supernatant containing soluble chromatin was isolated from the insoluble fraction. The relevant antibodies were incubated with 750 µg of Protein A Dynabeads (Novex) in PBS/BSA 0.5% rotating at 4˚C for 4 h. The beads were then washed three times in PBS/BSA 0.5%, to remove unbound antibody. Prior to immunoprecipitation, chromatin was diluted (20 mM Tris-HCl pH 8.0, 150 mM NaCl, 1 mM EDTA, 1% TritonX-100) 10-fold (or 50-fold for H3K4me3). Immunoprecipitations were performed using 1 ml of diluted chromatin per 25 µl of antibody coated beads, and rotated overnight at 4˚C. This is equivalent to $5x10^6$ cells (or $1x10^6$ for H3K4me3 ChIP). The IP was then washed with low salt buffer (0.1% SDS, 1% Triton X-100, 2 mM EDTA, 20 mM Tris-HCl pH8.0, 150 mM NaCl), then with high salt buffer (0.1% SDS, 1% Triton X-100, 2 mM EDTA, 20 mM Tris-HCl pH8.0, 500 mM NaCl), followed by LiCl buffer (0.25 M LiCl, 1% NP-40, 1% SDS, 1 mM EDTA, 10 mM Tris-HCl pH 8.0) and twice with TE (20 mM Tris-HCl pH8.0, 1 mM EDTA pH 8.0). Chromatin was eluted in 100 µl of elution buffer (100 mM $NaCO_3$, 0.1% SDS) by vigorous shaking for 30 min at 30°C. Crosslinks were reversed by the addition of 4 µl of 5 M NaCl and 2 µl of 500 µg/ml RNaseA (Roche) at 65˚C followed by incubation at 42˚C for 1.5 h with 1 µl of 20 mg/ml Proteinase K to remove protein. ChIP DNA was purified ChIP DNA Clean & Concentrator kit (Zymo Research).

*Cfp1^fl/fl^* ESCs were cultured for 96 h in presence or absence of 4-Hydroxytamoxifen (4-OHT). ChIP-seq was carried out in at least biological duplicate for each condition. To carry out ChIP an equal number of untreated and treated cells were resuspended in 10 mL of PBS and subjected to crosslink with 1% methanol-free formaldehyde (ThermoFisher) for 10 min at 25°C and quenched with 150 mM glycine for 10 min at RT. For T7-SET1A ChIP, cells were crosslinked in 2 µM disuccinimidyl glutarate (DSG, ThermoFisher) at 25° C for 50 mins and then 1% formaldehyde for 10 min at 25°C. Cells were lysed on ice for 10 min in 1 mL of lysis buffer (50 mM HEPES pH7.9, 300 mM NaCl, 1 mM EDTA, 0.5 mM EGTA, 0.5% NP-40, 0.1% Na deoxycholate, 0.1% SDS, 1x Complete EDTA-free inhibitor cocktail, 1 mM AEBSF) and then sonicated with Bioruptor Pico (Diagenode) for 20 min with 30s ON/OFF cycles (for double crosslinked chromatin, cells were sonicated for 23 min). Lysates were centrifuged for 10 min at 16000 g and the cleared chromatin was recovered and DNA quantified. For each IP, 300 µg of chromatin was diluted to 1 mL of lysis buffer and pre-cleared for 1 h at 4°C with IPA 300 resin beads (Repligen) blocked with yeast tRNA and BSA. Antibody was then added to each IP and incubated overnight, followed by

isolation of antibody chromatin complexes with 20 µL of blocked IPA300 beads per IP. Immunoprecipitates were washed 1x with lysis buffer, 1x with lysis buffer with 500 mM NaCl, 1x DOC buffer (10 mM Tris-HCl pH 8.0, 250 mM LiCl, 1 mM EDTA, 0.5% NP40, 0.5% Na- deoxycholate), and 2x with TE pH 8. Chromatin was then eluted in 200 µL of elution buffer (1% SDS, 0.1 M $NaHCO_3$) for 30 min at 30°C with vigorous shaking. Eluates and inputs were treated with DNase-free RNase (ThermoFisher) and crosslinks were reversed at 65˚C overnight with 200 mM NaCl and ProteinaseK solution (Sigma). DNA was purified with the ChIP DNA Clean & Concentrator kit (Zymo Research).
For massively parallel sequencing, DNAs were post-sonicated with Bioruptor Pico (Diagenode) to a DNA fragment size of 200-300 bp as determined by Bioanalyser analysis. Libraries were prepared with NEBNext Ultra DNA Library Prep Kit for Illumina and quantified by qPCR using KAPA Illumina DNA standards as reference. Libraries were sequenced either on an Illumina HiSeq2500 or NextSeq500.

Calibrated native ChIP-sequencing

ChIP sequencing for H3K4me3 in mouse ES cells was performed by calibrated native ChIP sequencing. Calibrated ChIP-seq was carried out in biological triplicate for each cell line and condition. This was achieved by adding $2.5x10^6$ *Drosophila* S2 (SG4) cells to $10x10^6$ untreated or tamoxifen treated *Cfp1*$^{fl/fl}$ ESCs. Nuclei were released by resuspending the mixed cell mixture in ice cold lysis buffer (10mM Tris-HCl pH 8.0, 10 mM NaCl, 3 mM $MgCl_2$, 0.1% NP40). Nuclei were then washed, and resuspended in 1 ml (10 mM Tris-HCl pH 8.0, 10 mM NaCl, 3 mM $MgCl_2$, 0.1% NP40, 0.25M sucrose, 3mM $CaCl_2$, 1x protease inhibitors (Sigma)), and incubated with 100U of MNase (Fermentas) at 37°C for 5 min followed by the addition of EDTA to halt the digestion. The supernatant was collected following centrifugation at 1500 g for 5 min at 4°C. The remaining pellet was incubated with 300 µl of nucleosome release buffer (10 mM Tris-HCl pH 7.5, 10 mM NaCl, 0.2 mM EDTA, 1x PIC) at 4°C for 1 h, then passed through a 27G needle using a 1 ml syringe, and spun at 1500 g for 5 min at 4°C. The two supernatants were combined and diluted 10-fold in native ChIP incubation buffer (70 mM NaCl, 10 mM Tris-HCl pH 7.5, 2 mM $MgCl_2$, 2 mM EDTA, 0.1% TritonX-100, 1x PIC). The relevant antibody was added to the diluted chromatin and rotated overnight at 4˚C. For each immunoprecipitation 20 µl of IPA300 agarose beads (RepliGen) were blocked with 1 mg/ml BSA and 1 mg/ml yeast tRNA in native ChIP incubation buffer (70 mM NaCl, 10 mM Tris-HCl pH 7.5, 2 mM $MgCl_2$, 2mM EDTA, 0.1% Triton X-100). Antibody/protein complexes were recovered by incubation with 20 µl of blocked beads per ChIP reaction, for 1 hour at 4°C, followed by centrifugation at 1000 g for 1 min. Beads were washed four times with native ChIP wash buffer (20 mM Tris-HCl pH 7.5, 2 mM EDTA, 125 mM NaCl, 0.1% Triton-X100), and once with 1 ml ice cold TE buffer. DNA was purified with the ChIP DNA Clean & Concentrator kit (Zymo Research). Libraries were prepared with NEBNext Ultra DNA Library Prep Kit for Illumina and quantified by qPCR using KAPA Illumina DNA standards as reference. Libraries were sequenced on an Illumina NextSeq500.

4sU RNA Sequencing

Nascent RNA sequencing was performed by pulse labelling with 4-thiouridine (4sU) as previously described (Radle et al., 2013). Confluent 15 cm plates were treated with 500 µM 4sU in 10 ml culture medium for 20 min. The 4sU-containing media was then aspirated and immediately replaced with 5 ml TRIZOL (Thermo Fisher). RNA was isolated by phenol-chloroform extraction using phase lock tubes (Eppendorf) and resuspend in nuclease-free water. Total RNA aliquots were treated with 6U Turbo

DNase (Ambion) and incubated at 37°C for 30 min, followed by inactivation of the DNase according to the manufacturer's instructions. 300 µg of the resulting total RNA were incubated with 2 µg Biotin-HPDP/µg RNA in biotinylation buffer (10 mM Tris-HCl pH 7.4 and 1 mM EDTA) and rotated for 1.5 h at room temperature. Unincorporated biotin-HPDP was removed by chloroform/isoamylalcohol extraction. To capture biotinylated RNA, µMACS streptavidin beads (Miltenyi) were added to the RNA suspension (1 µl of beads/ µg RNA) and rotated for 15 min at room temperature prior to loading onto µMACS minicolumns. Unlabelled RNA was removed by washing three times with 900 µl of wash buffer (100 mM Tris-HCl pH 7.5, 10 mM EDTA, 1 M NaCl, 0.1% Tween20) pre-heated at 65°C, then three more times with wash buffer at room temperature. Biotinylated RNA was then eluted in two rounds with 100 mM DTT and purified using an RNeasy miniElute kit (Qiagen). The RNA concentration was measured and at most 900 µg of biotinylated RNA was depleted of ribosomal RNAs using the Low Input RiboMinus Eukaryote System v2 kit (Thermo Fisher). The recovered rRNA-depleted biotinylated RNA was used to prepare the cDNA libraries with NEBNext Ultra Directional RNA Library Prep Kit for Illumina and subject to sequencing on Illumina NextSeq500 platform.

Quantitative nuclear RNA-seq

Cfp1$^{fl/fl}$ ESCs were cultured for 96 h in presence or absence of 4-OHT and the nuclear RNA-seq was carried out in biological quadruplicate for each condition. Cells were collected counted and 1x10^6 *Drosphila* S2 (SG4) cells were added to 4x10^6 cells from each condition. The cell mixture was recovered by centrifugation and resuspended at room temperature in lysis buffer (50 mM KCl, 10 mM $MgSO_4.7H_20$, 5 mM HEPES, 0.05% NP40) for 30 s and then incubated at room temperature for other 30 s and then chilled on ice. Nuclei were pelleted and gently washed in cold RBS buffer (10 mM NaCl, 10 mM Tris-HCl pH 7.4, 3 mM $MgCl_2$) three times. At the last wash step, an aliquot of nuclei corresponding to 4x10^5 cells was collected for genomic DNA extraction, whilst the remaining nuclei were pelleted and resuspended in TRIZOL reagent (Life Technologies) and subject to conventional RNA extraction. Nuclear RNA was then treated with DNase (Ambion) for 1 h at 37°C to remove any genomic DNA contamination. The RNA concentration was measured and 900 µg of nuclear RNA was depleted of ribosomal RNAs using the NEBNext rRNA Depletion kit. The recovered rRNA-depleted RNA was used to prepare the cDNA libraries with NEBNext Ultra Directional RNA Library Prep Kit for Illumina. 10 ng of sonicated gDNA was used to prepare "input" DNA libraries using the NEBNext Ultra DNA Library Prep Kit for Illumina. nucRNA and gDNA libraries were quantified by qPCR using KAPA Illumina DNA standards as reference and pooled at equimolar ratios (gDNA libraries pool was used at 1/10th of nucRNA libraries pool) and sequenced on the Illumina NextSeq500 platform.

ChIP-seq datasets processing

Reads were aligned to the mouse mm10 genome using bowtie2 (Langmead and Salzberg, 2012) with the '-no-mixed' and '-no-discordant' options, and non-uniquely mapping reads were discarded. Calibrated ChIP-Seq datasets were aligned to a concatenated genome (mm10+dm6), and reads which mapped more than once were discarded. PCR duplicates were removed using SAMtools (Li et al., 2009).

Calibrated ChIP-Seq normalization

To calibrate H3K4me3 ChIP-seq sequencing, the number of mouse reads was randomly down sampled using the number of drosophila reads in each sample as a normalization point. Genomic DNA sequencing of the input mixture of mouse and drosophila was also used to account for any variance in cell mixture ratios. Calibrated native H3K4me3 ChIP-seq tracks were generated using DANPOS2 (Chen et al., 2013).

Peak calling

C127 NMIs and peaks of CFP1 or H3K4me3 enrichment were identified using the MACS algorithm (Zhang et al., 2008), with an effective genome size of $1.87x10^9$, and a false discovery rate "-q" of 0.01. For duplicate and triplicate data, peaks were called for each replicate against a matched control, and were required to overlap in all replicates. H3K4me3 peaks within 250 bp of one another were merged and considered as one peak.

ChIP-Seq quantification

We used RefSeq transcripts (genome.ucsc.edu, downloaded on 21/08/2015) considering only genes mapping to a unique location in the genome. In cases where multiple TSSs located within 750 bp of each other, one transcript was randomly selected to be included in our gene set. Generally, intervals of TSS+/-1kb were used for all quantifications. For the H3K4me3 datasets, reads were quantified within peaks overlapping a given TSS, due to the high variability of peak widths. Furthermore, for these datasets the TSSs with a RefSeq divergent transcript within 2 kb were excluded, to preserve the information on the asymmetry of the H3K4me3 peak. ChIP-Seq replicates were randomly downsampled using SAMtools according to their library size or for calibrated ChIP-Seq based on the spike-in read ratio (see Calibrated ChIP-Seq normalization). For paired-end sequencing, normalized fragment coverage was quantified using the summarizeOverlaps() function from GenomicFeatures (Lawrence et al., 2013) in the mode Union and considering only primary alignments (ScanBamFlag() option isNotPrimaryRead=FALSE). Replicate counts were then pooled and 8 pseudocounts were added prior to log transformation. FPKM were calculated based on an average library size, due to the nature of already normalized counts.

Nuclear RNA-seq processing

Nuclear RNA-seq (nucRNA-Seq) and input genomic DNA reads were initially aligned against concatenated (mm10+dm6) rRNA genomic sequence (GenBank: BK000964.3 and M21017.1) using bowtie2 to filter out rRNA fragments, prior to alignment against the mm10 and dm6 genomes using the STAR RNA-seq aligner (Dobin et al., 2013). To improve mapping reads which failed to map using STAR were aligned against the genome using bowtie2 and reads which mapped more than once were discarded. PCR duplicates were removed using SAMtools (Li et al., 2009) and reads from mouse or drosophila genome were segregated into different bam files.

Nuclear RNA-Seq quantification and differential expression analysis

We used RefSeq transcripts (genome.ucsc.edu, downloaded on 21/08/2015) considering only genes mapping to a unique location in the genome. In cases where multiple TSSs located within 750 bp of each other, one transcript was randomly selected to be included in our gene set. In order to normalize gene counts to the calibration control, dm6-mapped nucRNA bam files were first downsampled using SAMtools based on the mm10/dm6 ratio in the input genomic DNA to control for any cell count variability between individual experiments. Fragment coverage at non-overlapping gene bodies of dm6 RefSeq transcripts (genome.ucsc.edu, downloaded on 14/09/2016) was quantified using the summarizeOverlaps() function in GenomicFeatures (Lawrence et al., 2013) in the mode Union and considering only primary alignments (ScanBamFlag() option is NotPrimaryRead=FALSE). Further normalization and differential expression analysis were performed in R (v 3.3.0) using the DESeq2 package (Love et al., 2014). Briefly, dm6 counts were used to quantify sizeFactors for subsequent differential expression analysis of raw fragment counts at mm10 RefSeq transcripts. Normalized counts were extracted from the DESeq2 object for each sample and each gene and used in subsequent analysis to calculate FPKM values. FPKM were calculated based on an average library size, due to the nature of already normalized counts. Log2FoldChange counts were extracted from the DESeq2 results table. Significantly differentially expressed genes were defined as having and adjusted p-value less than or equal to 0.01 and a fold change in expression of at least 1.5 fold.

Definition of gene categories

For ESC, all TSSs containing one NMI peak (Long et al., 2013) within 250 bp were defined as NMI TSSs. CFP1 targets were defined based on the bimodal distribution of log2 transformed CFP1 fragment counts around the TSS with a cutoff of > 6.1. To control for background, a further log2 fold change cutoff between untreated and 4-OHT-treated cells was introduced as log2FC=0.9. Highly transcribed genes were defined according to the bimodal distribution of FPKM values as genes with a log2(FPKM) higher than -2.5.

Supplementary References

ALLEN, M. D., GRUMMITT, C. G., HILCENKO, C., MIN, S. Y., TONKIN, L. M., JOHNSON, C. M., FREUND, S. M., BYCROFT, M. & WARREN, A. J. 2006. Solution structure of the nonmethyl-CpG-binding CXXC domain of the leukaemia-associated MLL histone methyltransferase. *EMBO J,* 25**,** 4503-12.

BLACKLEDGE, N. P., LONG, H. K., ZHOU, J. C., KRIAUCIONIS, S., PATIENT, R. & KLOSE, R. J. 2012. Bio-CAP: a versatile and highly sensitive technique to purify and characterise regions of non-methylated DNA. *Nucleic Acids Res,* 40**,** e32.

BLACKLEDGE, N. P., ZHOU, J. C., TOLSTORUKOV, M. Y., FARCAS, A. M., PARK, P. J. & KLOSE, R. J. 2010. CpG islands recruit a histone H3 lysine 36 demethylase. *Mol Cell,* 38**,** 179-90.

BROOKES, E., DE SANTIAGO, I., HEBENSTREIT, D., MORRIS, K. J., CARROLL, T., XIE, S. Q., STOCK, J. K., HEIDEMANN, M., EICK, D., NOZAKI, N., et al. 2012. Polycomb associates genome-wide with a specific RNA polymerase II variant, and regulates metabolic genes in ESCs. *Cell Stem Cell,* 10**,** 157-70.

BUENROSTRO, J. D., GIRESI, P. G., ZABA, L. C., CHANG, H. Y. & GREENLEAF, W. J. 2013. Transposition of native chromatin for fast and sensitive epigenomic profiling of open chromatin, DNA-binding proteins and nucleosome position. *Nat Methods,* 10**,** 1213-8.

CHEN, K., XI, Y., PAN, X., LI, Z., KAESTNER, K., TYLER, J., DENT, S., HE, X. & LI, W. 2013. DANPOS: dynamic analysis of nucleosome position and occupancy by sequencing. *Genome Res,* 23**,** 341-51.

CIERPICKI, T., RISNER, L. E., GREMBECKA, J., LUKASIK, S. M., POPOVIC, R., OMONKOWSKA, M., SHULTIS, D. D., ZELEZNIK-LE, N. J. & BUSHWELLER, J. H. 2010. Structure of the MLL CXXC domain-DNA complex and its functional role in MLL-AF9 leukemia. *Nat Struct Mol Biol,* 17**,** 62-8.

DOBIN, A., DAVIS, C. A., SCHLESINGER, F., DRENKOW, J., ZALESKI, C., JHA, S., BATUT, P., CHAISSON, M. & GINGERAS, T. R. 2013. STAR: ultrafast universal RNA-seq aligner. *Bioinformatics,* 29**,** 15-21.

FARCAS, A. M., BLACKLEDGE, N. P., SUDBERY, I., LONG, H. K., MCGOURAN, J. F., ROSE, N. R., LEE, S., SIMS, D., CERASE, A., SHEAHAN, T. W., et al. 2012. KDM2B links the Polycomb Repressive Complex 1 (PRC1) to recognition of CpG islands. *Elife,* 1**,** e00205.

LANGMEAD, B. & SALZBERG, S. L. 2012. Fast gapped-read alignment with Bowtie 2. *Nat Methods,* 9**,** 357-9.

LAWRENCE, M., HUBER, W., PAGES, H., ABOYOUN, P., CARLSON, M., GENTLEMAN, R., MORGAN, M. T. & CAREY, V. J. 2013. Software for computing and annotating genomic ranges. *PLoS Comput Biol,* 9**,** e1003118.

LI, H., HANDSAKER, B., WYSOKER, A., FENNELL, T., RUAN, J., HOMER, N., MARTH, G., ABECASIS, G., DURBIN, R. & GENOME PROJECT DATA PROCESSING, S. 2009. The Sequence Alignment/Map format and SAMtools. *Bioinformatics,* 25**,** 2078-9.

LI, H., ILIN, S., WANG, W., DUNCAN, E. M., WYSOCKA, J., ALLIS, C. D. & PATEL, D. J. 2006. Molecular basis for site-specific read-out of histone H3K4me3 by the BPTF PHD finger of NURF. *Nature,* 442**,** 91-5.

LONG, H. K., SIMS, D., HEGER, A., BLACKLEDGE, N. P., KUTTER, C., WRIGHT, M. L., GRUTZNER, F., ODOM, D. T., PATIENT, R., PONTING, C. P., et al. 2013. Epigenetic conservation at gene regulatory elements revealed by non-methylated DNA profiling in seven vertebrates. *Elife,* 2**,** e00348.

LOVE, M. I., HUBER, W. & ANDERS, S. 2014. Moderated estimation of fold change and dispersion for RNA-seq data with DESeq2. *Genome Biol,* 15**,** 550.

MUELLER, F., KARPOVA, T. S., MAZZA, D. & MCNALLY, J. G. 2012. Monitoring dynamic binding of chromatin proteins in vivo by fluorescence recovery after photobleaching. *Methods Mol Biol,* 833**,** 153-76.

PENA, P. V., DAVRAZOU, F., SHI, X., WALTER, K. L., VERKHUSHA, V. V., GOZANI, O., ZHAO, R. & KUTATELADZE, T. G. 2006. Molecular mechanism of histone H3K4me3 recognition by plant homeodomain of ING2. *Nature,* 442**,** 100-3.
PICELLI, S., BJORKLUND, A. K., REINIUS, B., SAGASSER, S., WINBERG, G. & SANDBERG, R. 2014. Tn5 transposase and tagmentation procedures for massively scaled sequencing projects. *Genome Res,* 24**,** 2033-40.
RADLE, B., RUTKOWSKI, A. J., RUZSICS, Z., FRIEDEL, C. C., KOSZINOWSKI, U. H. & DOLKEN, L. 2013. Metabolic labeling of newly transcribed RNA for high resolution gene expression profiling of RNA synthesis, processing and decay in cell culture. *J Vis Exp*.
RAMON-MAIQUES, S., KUO, A. J., CARNEY, D., MATTHEWS, A. G., OETTINGER, M. A., GOZANI, O. & YANG, W. 2007. The plant homeodomain finger of RAG2 recognizes histone H3 methylated at both lysine-4 and arginine-2. *Proc Natl Acad Sci U S A,* 104**,** 18993-8.
RAN, F. A., HSU, P. D., WRIGHT, J., AGARWALA, V., SCOTT, D. A. & ZHANG, F. 2013. Genome engineering using the CRISPR-Cas9 system. *Nat Protoc,* 8**,** 2281-308.
XU, C., BIAN, C., LAM, R., DONG, A. & MIN, J. 2011. The structural basis for selective binding of non-methylated CpG islands by the CFP1 CXXC domain. *Nat Commun,* 2**,** 227.
ZHANG, Y., LIU, T., MEYER, C. A., EECKHOUTE, J., JOHNSON, D. S., BERNSTEIN, B. E., NUSBAUM, C., MYERS, R. M., BROWN, M., LI, W., et al. 2008. Model-based analysis of ChIP-Seq (MACS). *Genome Biol,* 9**,** R137.
ZHOU, J. C., BLACKLEDGE, N. P., FARCAS, A. M. & KLOSE, R. J. 2012. Recognition of CpG island chromatin by KDM2A requires direct and specific interaction with linker DNA. *Mol Cell Biol,* 32**,** 479-89.
