# Supplementary material for: The SET1 Complex Selects Actively Transcribed Target Genes via Multivalent Interaction with CpG Island Chromatin
Source: Cell Rep. 2017 Sep 5;20(10):2313–27. doi: 10.1016/j.celrep.2017.08.030 (PMC5603731; doi:10.1016/j.celrep.2017.08.030)
Supplement: Document S2. Article plus Supplemental Information [file mmc2.pdf]

Cell Reports

Article

The SET1 Complex Selects Actively Transcribed Target Genes via Multivalent Interaction with CpG Island Chromatin

Graphical Abstract

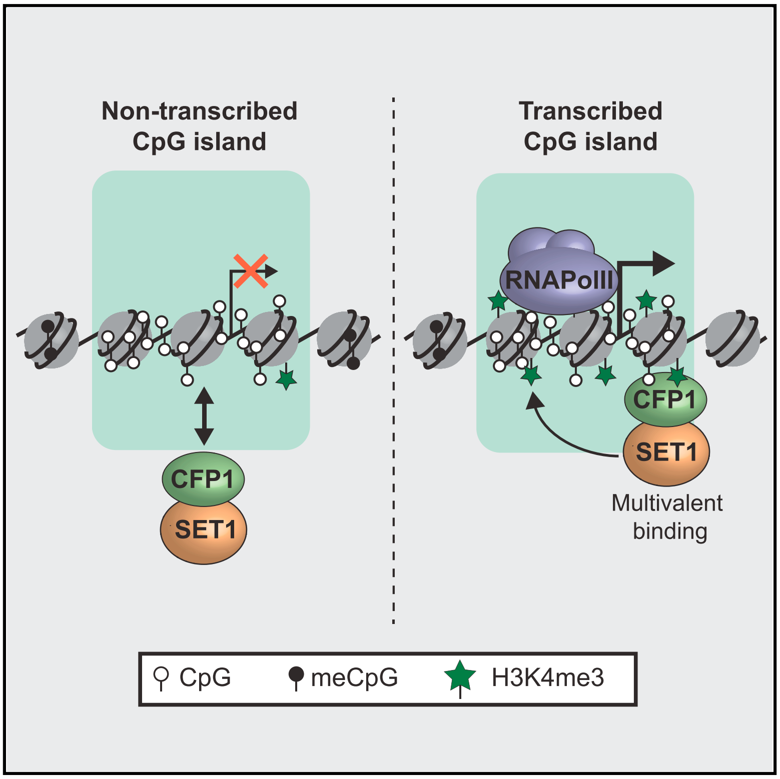

Authors

David A. Brown, Vincenzo Di Cerbo, Angelika Feldmann, ..., Tatiana G. Kutateladze, Haruhiko Koseki, Robert J. Klose

Correspondence

rob.klose@bioch.ox.ac.uk

In Brief

Brown et al. show that the SET1 complex is driven to active CpG island promoters via the CFP1 protein, which engages in multivalent chromatin binding to recognize both non-methylated DNA and H3K4me3. This is necessary for normal H3K4me3 at active gene promoters and appropriate regulation of gene expression.

Highlights

- The CFP1/SET1 complex engages in dynamic and stable chromatin-binding events
- CFP1 uses multivalent chromatin interactions to select active CpG island promoters
- SET1A occupancy at CpG island promoters is predominately defined by CFP1
- CFP1 targets SET1 to shape promoter-associated H3K4me3 and gene expression

Accession Numbers

GSE93538

Brown et al., 2017, Cell Reports *20*, 2313–2327
September 5, 2017 © 2017 The Authors.
http://dx.doi.org/10.1016/j.celrep.2017.08.030

Cell Reports
Article

OPEN ACCESS
CellPress

The SET1 Complex Selects Actively Transcribed Target Genes via Multivalent Interaction with CpG Island Chromatin

David A. Brown,[1,5] Vincenzo Di Cerbo,[1,5] Angelika Feldmann,[1] Jaewoo Ahn,[3] Shinsuke Ito,[2] Neil P. Blackledge,[1] Manabu Nakayama,[2] Michael McClellan,[4] Emilia Dimitrova,[1] Anne H. Turberfield,[1] Hannah K. Long,[1] Hamish W. King,[1] Skirmantas Kriaucionis,[4] Lothar Schermelleh,[1] Tatiana G. Kutateladze,[3] Haruhiko Koseki,[2] and Robert J. Klose[1,6,*]
[1]Department of Biochemistry, University of Oxford, Oxford, OX1 3QU, UK
[2]Laboratory for Developmental Genetics, RIKEN Center for Integrative Medical Sciences (IMS), 1-7-2 Suehiro-cho, Tsurumi-ku, Yokohama, Kanagawa 230-0045, Japan
[3]Department of Pharmacology, University of Colorado School of Medicine, Aurora, CO 80045, USA
[4]Ludwig Cancer Research, Nuffield Department of Medicine, University of Oxford, Oxford OX3 7DQ, UK
[5]These authors contributed equally
[6]Lead Contact
*Correspondence: rob.klose@bioch.ox.ac.uk
http://dx.doi.org/10.1016/j.celrep.2017.08.030

SUMMARY

Chromatin modifications and the promoter-associated epigenome are important for the regulation of gene expression. However, the mechanisms by which chromatin-modifying complexes are targeted to the appropriate gene promoters in vertebrates and how they influence gene expression have remained poorly defined. Here, using a combination of live-cell imaging and functional genomics, we discover that the vertebrate SET1 complex is targeted to actively transcribed gene promoters through CFP1, which engages in a form of multivalent chromatin reading that involves recognition of non-methylated DNA and histone H3 lysine 4 trimethylation (H3K4me3). CFP1 defines SET1 complex occupancy on chromatin, and its multivalent interactions are required for the SET1 complex to place H3K4me3. In the absence of CFP1, gene expression is perturbed, suggesting that normal targeting and function of the SET1 complex are central to creating an appropriately functioning vertebrate promoter-associated epigenome.

INTRODUCTION

Gene expression is controlled by transcription factors that bind to DNA sequences in gene regulatory elements and control how RNA polymerase engages with transcription start sites (Levine et al., 2014). However, in eukaryotes, nucleosomes can limit accessibility to DNA sequences and create a barrier to the gene regulatory apparatus (Lorch et al., 1987). To counteract this, post-translational modifications on histones at gene regulatory elements can alter chromatin structure or recruit reader proteins that regulate access to DNA and help to shape gene expression (Piunti and Shilatifard, 2016; Venkatesh and Workman, 2015). Many of the most prevalent histone modifications have been extensively mapped within vertebrate genomes (ENCODE Project Consortium, 2012). However, how the chromatin-modifying complexes that place these modifications recognize their appropriate target sites and affect gene expression remains poorly understood.

Methylation of histone H3 on lysine 4 (H3K4me) is evolutionarily conserved from yeast to human and widely associated with gene regulatory elements, where it is thought to support gene activity (reviewed by Kusch, 2012 and Shilatifard, 2012). H3K4me can occur in distinct states, with monomethylation (me1) predominating at distal regulatory elements and dimethylation (me2) and trimethylation (me3) predominating at active gene promoters in vertebrates (Bernstein et al., 2005; Heintzman et al., 2007, 2009; Schneider et al., 2004). H3K4me can be placed by six large multi-protein complexes (van Nuland et al., 2013) that are distinguishable based on the identity of their catalytic subunits, which correspond to MLL1, MLL2, MLL3, MLL4, SET1A, or SET1B (reviewed by Shilatifard, 2012). MLL3/4 complexes deposit H3K4me1 at distal regulatory elements (Hu et al., 2013a; Kaikkonen et al., 2013; Lee et al., 2013), whereas MLL1/2 and SET1A/B are thought to place H3K4me at gene promoters (Andreu-Vieyra et al., 2010; Bledau et al., 2014; Denissov et al., 2014; Milne et al., 2002; Wang et al., 2009). In most cell types, the SET1 complexes are the predominant H3K4 methyltransferases (Ardehali et al., 2011; Bledau et al., 2014; Hallson et al., 2012) and H3K4me is thought to act as a nucleation site for binding of reader proteins that elicit effects on chromatin structure and gene regulation (Lauberth et al., 2013; Li et al., 2006; Peña et al., 2006; Shi et al., 2006; Vermeulen et al., 2007; Wysocka et al., 2006). Deletion of SET1A in mice results in early embryonic lethality due to a failure of embryos to gastrulate, illustrating a fundamental role for the SET1A complex in mammalian development (Bledau et al., 2014).

Attempts to dissect the function of vertebrate H3K4 methyltransferases at gene promoters have been limited by our relatively naive understanding of how these enzymes recognize their target sites in the genome. In budding yeast, the recruitment and activity of the sole H3K4 methyltransferase complex is proposed to rely on an association with RNA polymerase II (RNA PolII)

This is an open access article under the CC BY license (http://creativecommons.org/licenses/by/4.0/).

through its phosphorylated C-terminal heptapeptide repeat (CTD) (Ng et al., 2003). This process may be conserved in vertebrates because WDR82, a component of the SET1 complex, has also been proposed to integrate SET1 activity with gene transcription via interaction with the CTD of RNA PolII (Austenaa et al., 2015; Lee and Skalnik, 2008; Wu et al., 2008). However, the relevance of co-transcriptional recruitment to the binding and activity of the vertebrate SET1 complex remains unclear because H3K4 methyltransferase targeting in higher eukaryotes appears to be much more complex. For example, both MLL1/2 and CFP1, a component of the SET1 complex, contain a CXXC DNA-binding domain that can recognize non-methylated CpG dinucleotides found in promoter-associated regulatory elements called CpG islands (CGIs) (Birke et al., 2002; Clouaire et al., 2012; Denissov et al., 2014; Long et al., 2013a; Thomson et al., 2010; Voo et al., 2000). However, generic CGI recognition does not explain why the MLL1/2 and SET1 complexes appear to regulate H3K4me at distinct subsets of target genes in a gene expression-dependent manner (Denissov et al., 2014; Hu et al., 2013b). Furthermore, site-specific DNA binding transcription factors or long non-coding RNAs have also been implicated in recruiting the MLL1/2 and SET1 complexes (Voigt et al., 2013). Therefore, the molecular mechanisms that shape how H3K4 methyltransferases select their target sites remain poorly defined and represent a central conceptual gap in our understanding of the promoter-associated epigenome.

Given the fundamental role that the SET1 complex plays in depositing H3K4me and sustaining normal development, here we have focused on understanding how this complex is targeted to chromatin. By combining live-cell imaging and functional genomics, we discover that the CFP1 component of the SET1 complex preferentially binds to CGIs of actively transcribed genes through multivalent interaction with chromatin, which requires recognition of non-methylated DNA and H3K4me3. We demonstrate that CFP1 is the predominant targeting module for the SET1A complex, whereas co-transcriptional recruitment appears to play only a minor role in SET1A occupancy. Importantly, CFP1 guides H3K4me3 deposition by the SET1A complex and is required for the appropriate expression of a subset of its target genes.

RESULTS

Interaction with the SET1 Complex Is the Central Determinant of CFP1 Dynamics In Vivo

It has been proposed that CFP1 plays a key role in regulating SET1 complex function. This is based on work that described CFP1 occupancy at CGI elements (Denissov et al., 2014; Thomson et al., 2010) and defects in H3K4me3 resulting from its deletion in embryonic stem cells (ESCs) (Carlone et al., 2005; Clouaire et al., 2012, 2014; Tate et al., 2009). However, how CFP1 dynamics and chromatin binding are achieved in vivo, and whether these are central determinants in guiding the SET1 complex to genomic target sites, remains largely unknown. To begin addressing these questions, we stably expressed GFP-CFP1 in a mouse epithelial cell line (Figures S1A–S1D) that is suited to live-cell imaging, and examined the mobility of nuclear CFP1 by fluorescence recovery after photobleaching (FRAP). This revealed that CFP1 is highly mobile, with $t_{1/2}$ recovery times in the nucleoplasm of $\sim$1 s (Figures 1A–1C and S1F–S1I). To understand if CFP1 dynamics are determined by its capacity to interact with chromatin, we engineered single amino acid mutations into CFP1 that disrupt either the function of its non-methylated DNA-binding CXXC domain or its PHD domain, which is proposed to bind to H3K4me (Figures 1A and S2) (Eberl et al., 2013; Mahadevan and Skalnik, 2016). Cell lines were established, in which individual GFP-CFP1 mutants were stably expressed at comparable levels to wild-type GFP-CFP1, and, importantly, we verified that these mutations did not affect association of CFP1 with the SET1A complex (Figures S1A–S1E). Interestingly, mutation of the CXXC or PHD domain caused a small but significant increase in the mobility of CFP1 compared to wild-type protein, but this was not further increased by mutation of both domains (Figures 1B and 1C).

When we examined the dynamics of GFP-SET1A, we observed that it was slightly less mobile than GFP-CFP1 (Figures 1D, S1A, and S1B), consistent with a possible role for the SET1A interaction in limiting CFP1 mobility independent of the CXXC and PHD domains (Figure 1E). Therefore, we generated a GFP-CFP1 SET1 interaction domain (SID) mutant cell line, in which CFP1 association with SET1A was disrupted by a two amino acid substitution mutation in an alpha helix of the previously mapped interaction domain (Figures 1D, S1A–S1E, and S2E) (Tate et al., 2009). Strikingly, when compared to wild-type CFP1, the CFP1 SID mutant was dramatically more mobile (Figure 1E). Importantly, combining the CXXC and PHD mutations with the SID mutation further increased CFP1 mobility to the point where it approached the diffusion of free GFP (Figures 1D–1F). Together, this demonstrates that inclusion in the SET1 complex predominates in defining the nuclear dynamics of CFP1, with the CXXC and PHD domains making additional, more modest contributions.

Targeting of CFP1 to CGIs Relies on the CXXC and PHD Domains but Not Interaction with SET1

We next wanted to understand how individual domains of CFP1 contribute to the more stable binding of CFP1 at target sites on the genome. To achieve this, we used chromatin immunoprecipitation sequencing (ChIP-seq) to map CFP1 binding genome-wide (Figures 2A and 2B). In parallel, Bio-CAP-seq was used to map non-methylated islands (NMIs), which generally correspond to CGIs (Blackledge et al., 2012; Illingworth et al., 2010; Long et al., 2013b). This revealed that 91.5% of CFP1 peaks occurred at NMIs, but only 38.1% of NMIs were occupied by CFP1 (Figure 2C), indicating that NMI occupancy of CFP1 is not uniform, as described previously (Denissov et al., 2014). Further analysis revealed that CFP1 was enriched at NMIs that had features usually associated with active transcription start sites (TSSs), including H3K4me3 and RNA PolII (Figures 2A and 2B). Indeed, CFP1-bound NMIs were more frequently associated with annotated TSSs and had elevated H3K4me3 and RNA PolII compared to NMI TSSs not bound by CFP1 (Figures 2D–2F).

To explore the determinants of CFP1 binding, we carried out ChIP-seq using a GFP-specific antibody in lines stably expressing GFP alone, wild-type GFP-CFP1, or GFP-CFP1 with

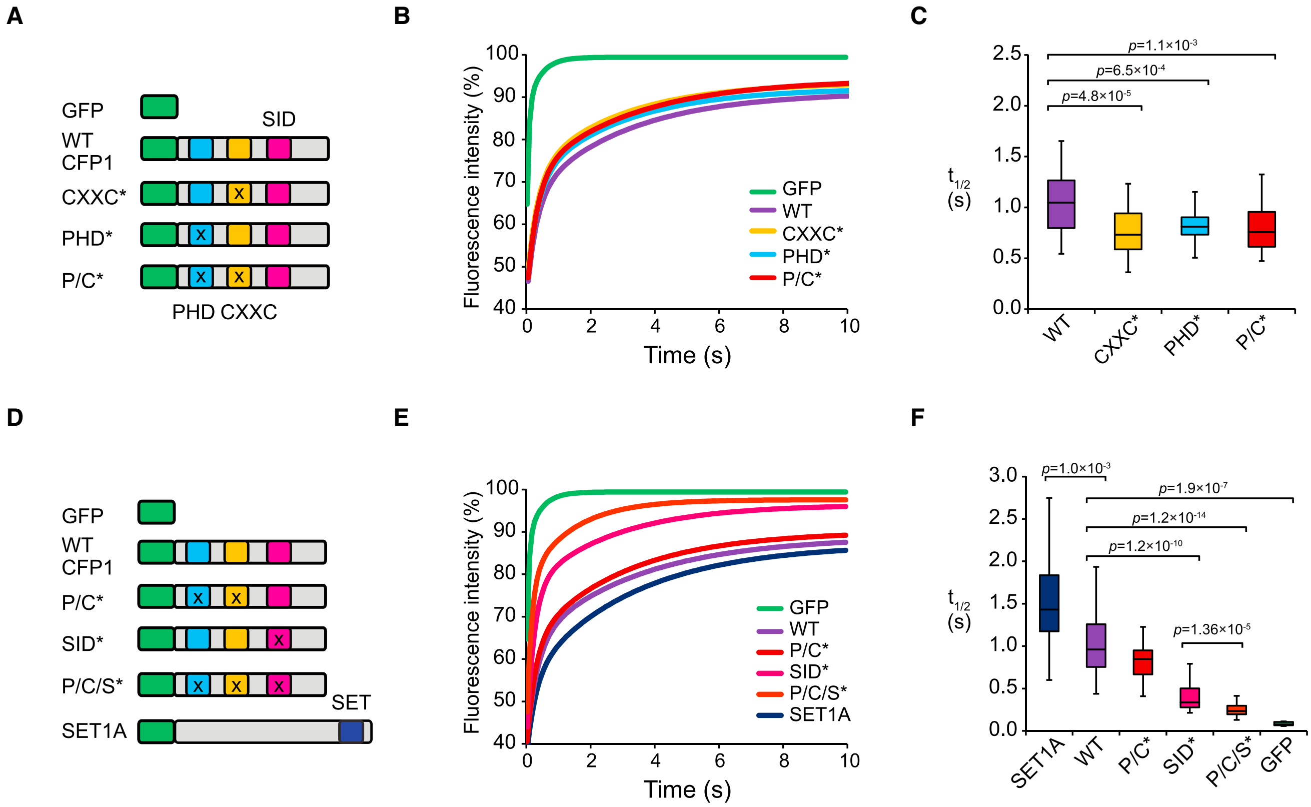

Figure 1. The Cellular Dynamics of CFP1 Are Governed by Its Chromatin-Binding Domains and Association with the SET1A Complex

(A) A schematic illustrating the GFP-tagged versions of CFP1 stably expressed in mouse C127 cells and used in the FRAP studies in (B). These include GFP alone (GFP), wild-type CFP1 (WT), CFP1 with a mutated CXXC domain (CXXC*), CFP1 with a mutated PHD domain (PHD*), and CFP1 with combined CXXC and PHD mutations (P/C*).

(B) Biexponential fits describing the recovery of fluorescence intensity over time for each of the proteins described in (A). Fits were calculated using post-bleach fluorescence intensity recovery data collected at 8 frames per second from >42 cells across biological triplicates.

(C) A boxplot indicating the half time of recovery ($t_{1/2}$) in seconds for the FRAP curves in (B). Boxes show interquartile range (IQR) and whiskers extend by 1.5 × IQR. The p values indicating statistically significant differences are indicated above the boxplot. The p value denotes statistical significance using a Student's t test.

(D) A schematic illustrating the GFP-tagged versions of CFP1 and SET1A used in the FRAP studies in (E). This includes GFP alone (GFP), wild-type CFP1 (WT), CFP1 with combined PHD and CXXC mutations (P/C*), CFP1 with a mutated SET1 interaction domain (SID*), CFP1 with combined PHD/CXXC/SID mutations (P/C/S*), and wild-type SET1A.

(E) Biexponential fits describing the recovery of fluorescence intensity over time for the proteins described in (D). CFP1 and SET1 fits were calculated using post-bleach fluorescence intensity recovery data collected at 13 frames per second from >28 cells across biological triplicates, whereas the GFP fit was limited to 8 cells, in which the nuclear border was clearly defined.

(F) A boxplot indicating the half time of recovery ($t_{1/2}$) in seconds for the FRAP curves in (E). Boxes show IQR and whiskers extend by 1.5 × IQR. The p values indicating statistically significant differences are indicated above the boxplot. The p value denotes statistical significance using a Student's t test.

mutations in the CXXC, PHD, CXXC/PHD, or SID (Figures 2G–2I). Importantly, GFP-CFP1 enrichment correlated well with endogenous CFP1 (Figure S3). Strikingly, mutating the CXXC domain completely abrogated CFP1 association with NMIs, whereas mutation of the PHD domain also resulted in a dramatic but not complete loss of NMI occupancy. Surprisingly, mutation of the SID resulted in the opposite effect, causing CFP1 to bind more efficiently to NMIs (Figures 2G–2I). Together, our observations suggest that DNA and chromatin binding by the CXXC and PHD domains define stable CFP1 accumulation at NMI target sites, whereas SET1 association, which predominates in shaping CFP1 nuclear dynamics, limits accumulation at these regions (see Discussion).

Multivalent Binding to Non-methylated DNA and H3K4me3 Determines the Occupancy of CFP1 on Chromatin In Vivo

The CFP1 CXXC domain is known to bind non-methylated DNA (Voo et al., 2000; Xu et al., 2011), and the PHD domain has been reported to bind H3K4me (Eberl et al., 2013). Therefore, we set out to examine whether CFP1 utilizes a combination of its CXXC and PHD domains to select NMIs that have both non-methylated DNA and H3K4me. If this was the case, we hypothesized that CFP1 binding to NMIs would be related to H3K4me3 levels and differ from stereotypical NMI-binding proteins like KDM2B that only read non-methylated DNA (Blackledge et al., 2010, 2014; Farcas et al., 2012). To test this

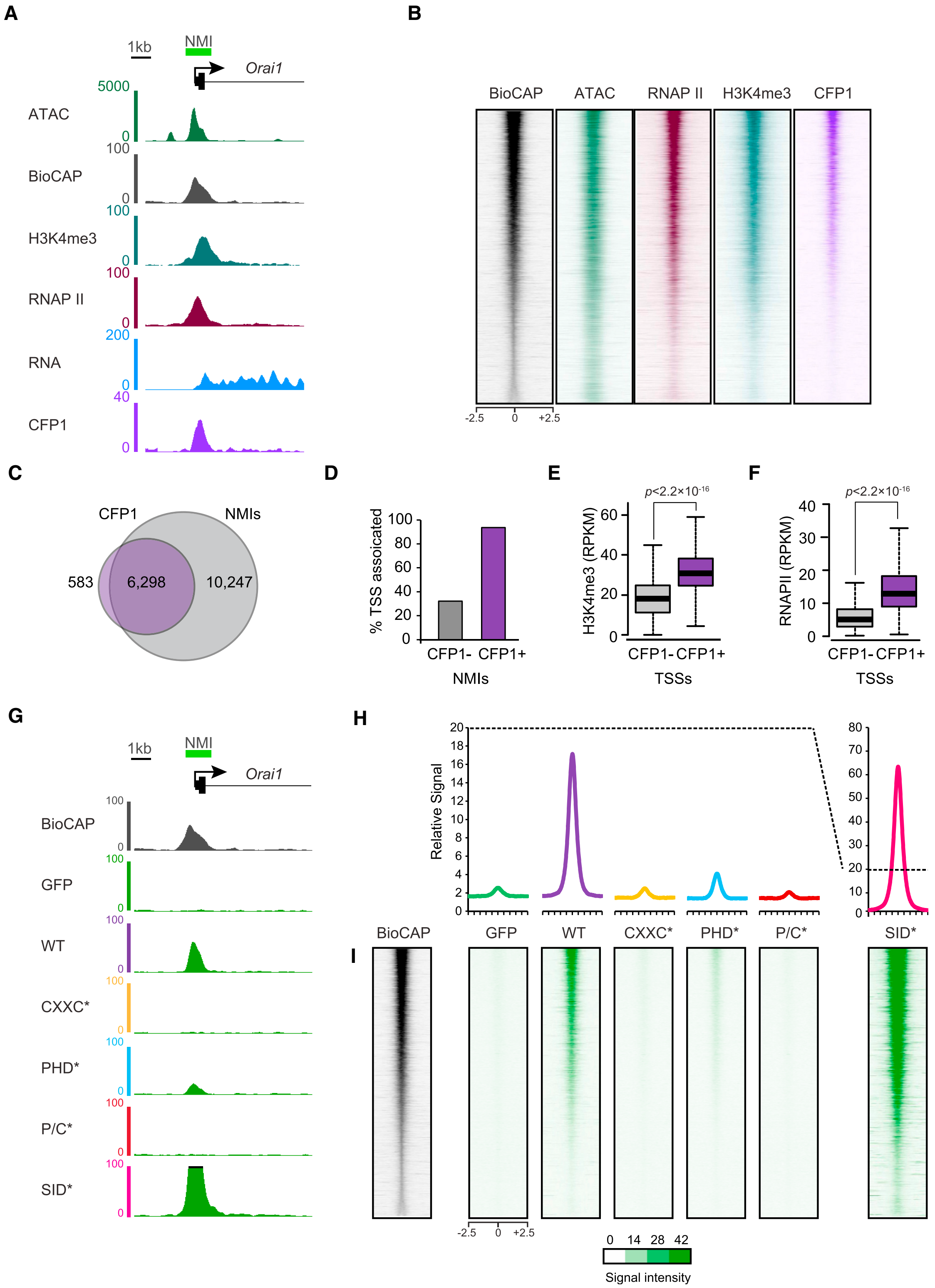

Figure 2. Binding of CFP1 to Chromatin Relies on the CXXC and PHD Domains but Not Interaction with SET1

(A) A genomic snapshot of the NMI-associated *Orai1* gene promoter showing the signal from ATAC, Bio-CAP, H3K4me3, RNA Pol II, RNA, and CFP1 sequencing experiments.

(B) Heatmaps of the sequencing signals in (A) ranked by Bio-CAP signal over all NMIs.

(legend continued on next page)

possibility, we binned all NMIs by their H3K4me3 percentile and plotted the relative enrichment of non-methylated DNA (Bio-CAP), CFP1, GFP-CFP1, and KDM2B signal over these regions (Figures 3A and 3B). As expected, KDM2B occupancy scaled nearly linearly with non-methylated DNA despite increasing H3K4me3 (Figure 3B). In contrast, CFP1 and GFP-CFP1 diverged from this linearity with increased occupancy at NMIs with elevated H3K4me3 (Figure 3B). Importantly, however, residual ChIP-seq signal in the GFP-CFP1 PHD mutant exhibited a near-linear relationship with non-methylated DNA (Figures 3A and 3B).

Our observations indicate that CFP1 preferentially binds to H3K4me3-enriched NMIs via its PHD domain (Figures 3A and 3B). However, given that PHD domains can display varying affinities for individual methylation states (Musselman et al., 2012), we were keen to better characterize the binding preference of the CFP1 PHD domain. To achieve this, we generated the recombinant CFP1-PHD domain and examined its binding to unmodified (me0), me1, me2, and me3 H3K4 peptides in vitro (Figures 3C–3H). First, we examined the binding of the CFP1 PHD finger to a H3K4me3 peptide in ^{1}H,^{15}N heteronuclear single quantum coherence titration experiments. Addition of the H3K4me3 peptide to the ^{15}N-labeled CFP1 PHD domain induced substantial chemical shift perturbations, suggesting a tight interaction of the CFP1 PHD domain with H3K4me3. In contrast, interaction with H3K4me0 was considerably weaker, as evident from small chemical shift perturbations and fast exchange (Figure 3D). Quantitative measurements of binding affinities using intrinsic fluorescence spectroscopy (Figures 3E–3G) further supported the NMR results and revealed that the CFP1 PHD domain binds preferentially to the H3K4me3 (Kd 1.3 μM), with H3K4me2/me1/me0 having considerably lower affinities (9.1 μM, 64 μM, and 373 μM, respectively) (Figure 3H). Interestingly, the in vitro affinity of the PHD domain for H3K4me3 is of similar magnitude to that of the isolated CXXC domain for non-methylated DNA (Kd $\sim$2.5–4.4 μM) (Risner et al., 2013; Xu et al., 2011), further supporting the idea that binding to both H3K4me3 and non-methylated DNA are important affinity features that define normal CFP1 occupancy at NMI target sites. Therefore, our in vivo ChIP-seq analysis demonstrates that the PHD domain is required for the appropriate enrichment of CFP1 at NMIs with elevated H3K4me3 (Figures 3A and 3B) and our in vitro analysis reveals that the PHD domain of CFP1 preferentially binds to H3K4me3.

CFP1 Is the Central Determinant in SET1A Occupancy on Chromatin

Previous CFP1 studies have utilized a mouse ESC line isolated from a *Cfp1*$^{-/-}$ embryo. However, these *Cfp1*$^{-/-}$ ESCs display global reductions in genomic DNA methylation and other epigenetic defects (Carlone et al., 2005; Clouaire et al., 2012), presumably due to prolonged culture without CFP1. To overcome this limitation, we derived an ESC line from a CFP1 conditional knockout mouse (*Cfp1*$^{fl/fl}$), in which addition of tamoxifen induces *Cfp1* deletion (Figures 4A and S4A). Following 96 hr of tamoxifen treatment, CFP1 protein and target site occupancy was undetectable (Figures 4B–4D and S4H), but no global effects on DNA methylation were observed (Figure S4B).

Because CFP1 forms a complex with SET1A (Lee and Skalnik, 2005; van Nuland et al., 2013), we wanted to use the *Cfp1*$^{fl/fl}$ ESCs to understand whether CFP1 contributes to SET1A occupancy on chromatin. The genome-wide occupancy of SET1A has remained poorly defined, and we were unable to ChIP SET1A using commercially available antibodies. We therefore used CRISPR/Cas9 technology to engineer epitope tags onto both copies of the *Set1a* gene in the *Cfp1*$^{fl/fl}$ ESCs (Figures S4C–S4E). ChIP-seq for epitope-tagged SET1A in the untreated *Cfp1*$^{fl/fl}$ ESCs revealed that SET1A and CFP1 occupancy at TSSs was highly correlated (R = 0.95) and SET1A enrichment was greatest at CFP1-bound NMI TSSs (Figures 4E–4G and S4I). Strikingly, following tamoxifen treatment to remove CFP1, we observed a major reduction in SET1A occupancy (Figures 4E, 4F, 4H, and S4G), with little alteration to SET1A protein levels (Figure S4F). Importantly, loss of SET1A binding was highly correlated with the initial level of CFP1 at individual sites (R = $-$0.81) (Figure 4H). Interestingly, following removal of CFP1, highly transcribed genes exhibited some SET1A retention (Figures 4I, 4J, and S4J). This suggests that at some target sites, a secondary targeting modality contributes to SET1A occupancy. This may involve co-transcriptional recruitment of SET1A via direct interaction with RNA PolII or other features of these genes. Nevertheless, our observations indicate that SET1A is primarily recruited to chromatin by CFP1.

CFP1 Exploits Multivalent Interactions with CGI Chromatin to Shape H3K4me3

We next wanted to examine if loss of CFP1-dependent recruitment affected the ability of the SET1A complex to place H3K4me. Western blot analysis of bulk H3K4me revealed that H3K4me3 was reduced in CFP1-deleted cells, whereas H3K4me1 and H3K4me2 were largely unaffected (Figure 5A). To understand more about where H3K4me3 was lost in the genome, we carried out native ChIP-seq for H3K4me3 in untreated and tamoxifen-treated *Cfp1*$^{fl/fl}$ ESCs using a calibrated approach (Figures 5B and 5C) (Hu et al., 2015). This revealed that H3K4me3 loss was most pronounced at the TSSs of NMI-associated genes that had broad peaks of H3K4me3 and higher levels of CFP1 binding (Figures S5A–S5C). Furthermore,

(C) A Venn diagram illustrating the overlap between CFP1 peaks and NMIs.
(D) Bar graph illustrating the percentage of CFP1-bound and -unbound NMIs that overlap with transcription start sites (TSSs).
(E) Boxplots illustrating the enrichment of H3K4me3 at TSS-associated NMIs that are bound (CFP1+) or unbound (CFP1$-$) by CFP1. Boxes show IQR and whiskers extend by 1.5 × IQR. The p value denotes statistical significance calculated by a Wilcoxon signed rank test.
(F) As in (E), illustrating enrichment of RNA PolII.
(G) A genomic snapshot of the NMI-associated *Orai1* gene promoter showing the GFP ChIP-seq signal for GFP, GFP-CFP1, and mutated forms of GFP-CFP1.
(H) Metaplot and heatmap analysis of GFP ChIP-seq signal for GFP, GFP-CFP1, and mutated forms of GFP-CFP1 over all NMIs.
(I) Heatmap analysis of GFP ChIP-seq signal over all NMIs for the same cell lines as in (H).

OPEN ACCESS
CellPress

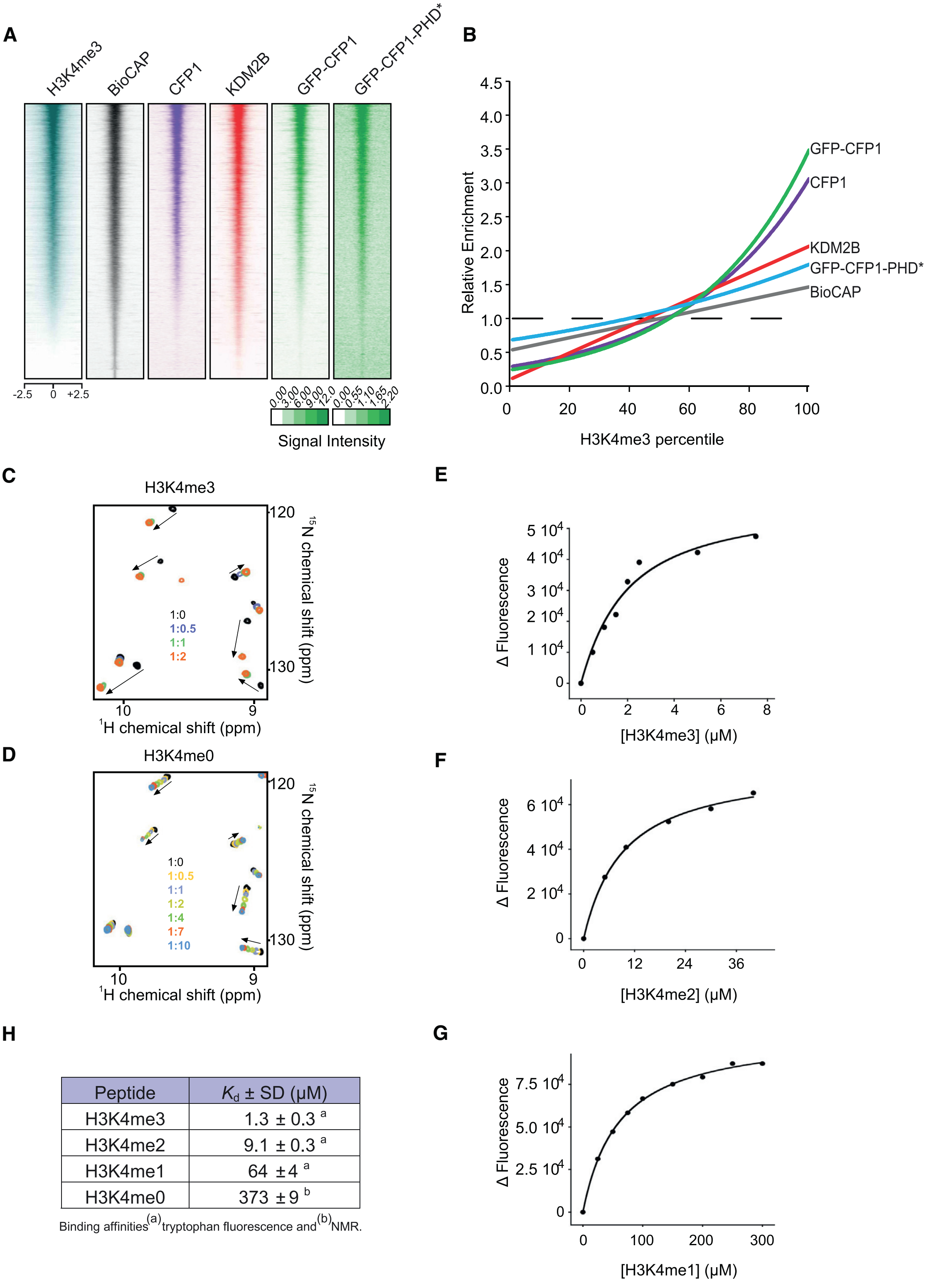

Peptide	K_d ± SD (μM)
H3K4me3	1.3 ± 0.3 [a]
H3K4me2	9.1 ± 0.3 [a]
H3K4me1	64 ± 4 [a]
H3K4me0	373 ± 9 [b]

Binding affinities[(a)]tryptophan fluorescence and[(b)]NMR.

Figure 3. Multivalent Binding to Non-methylated DNA and H3K4me3 Determines the Occupancy of CFP1 on Chromatin In Vivo

(A) Heatmap analysis of Bio-CAP and ChIP-seq signal over all NMIs for the indicated endogenous or GFP fusion proteins. The intensity scale for GFP-CFP1 and GFP-CFP1-PHD* mutant are indicated below.

(B) The relative enrichment of the features heatmapped at NMIs in (A) was plotted across H3K4me3 enrichment percentiles.

(legend continued on next page)

consistent with CFP1 occupying actively transcribed gene promoters, effects on H3K4me3 following CFP1 loss were prevalent at highly transcribed, as opposed to lowly or non-transcribed, genes (Figure 5D) and also at genes with high RNA PolII occupancy (Figure S5D). This is in agreement with previous observations suggesting that CFP1 contributes to H3K4me3 at actively transcribed genes (Clouaire et al., 2012, 2014).

We were next keen to understand if normal H3K4me3 levels relied on the capacity of CFP1 to engage in multivalent chromatin interactions and bind to SET1. We therefore performed a series of H3K4me3 ChIP-seq experiments in tamoxifen-treated *Cfp1^fl/fl^* ESCs that had been rescued with either wild-type or mutant forms of CFP1 (Figures S5E–S5G). Focusing our analysis on NMIs with broad H3K4me3 peaks that are most reliant on CFP1, we observed that GFP expression alone was unable to rescue H3K4me3 defects, whereas GFP-CFP1 fully restored H3K4me3 (Figures 5E–5G). In contrast, mutation of the CXXC/PHD domains or the SID failed to rescue H3K4me3 defects (Figures 5E–5G). This demonstrates that the capacity of CFP1 to engage in multivalent interactions with chromatin and to associate with SET1 is required for normal deposition of H3K4me3.

Loss of CFP1 Leads to Widespread Effects on Gene Expression

Although H3K4me3 enrichment at gene promoters correlates with gene expression (Kusch, 2012), the contribution of H3K4me3 to transcription remains unclear. To examine the influence of CFP1 on gene expression, we carried out quantitative nuclear RNA sequencing (RNA-seq) in $Cfp1^{fl/fl}$ ESCs with or without tamoxifen treatment. We focused our analysis on CFP1-bound NMI-associated genes (9,979) and identified significantly misregulated genes (false discovery rate [FDR] $\leq$ 0.01) that had changes in expression of at least 1.5-fold. Importantly, following CFP1 deletion, CFP1 target genes predominantly exhibited reduced expression (1,108), whereas a smaller subset (584) showed increased expression (Figures 6A, S6B, and S6C). In contrast, genes not bound by CFP1 showed a similar number of increases and decreases in gene expression (Figure S6A). These trends appear to support a role for CFP1 in potentiating gene expression. However, given the appreciable number of CFP1 target genes that are upregulated in response to CFP1 deletion, this may point to uncharacterized roles for CFP1/SET1 in gene repression or to secondary effects on gene expression.

Interestingly, when compared to unaffected genes, CFP1 target genes with reduced expression tended to be lowly to moderately expressed (Figure 6B). Furthermore, when compared to upregulated CFP1 target genes, they had elevated CpG density and CFP1 at their TSSs (Figures 6C and 6D) as well as moderately higher levels of SET1A (Figure 6E). However, we found no obvious correlation between the reductions in gene expression and effects on H3K4me3 following removal of CFP1 ($R = -0.11$) (Figure 6F), indicating that the level of H3K4me3 loss does not define how the associated gene will respond transcriptionally. Together, these observations indicate that the CFP1/SET1 complex plays an important role in shaping gene expression from transcribed CGI-associated genes, with moderately expressed genes appearing more sensitive to CFP1 loss than more highly expressed genes (see Discussion).

DISCUSSION

Here, we discover that CFP1, a component of the SET1 complex, uses a type of multivalent interaction with chromatin, which relies on its CXXC and PHD domains to identify target gene promoters (Figures 1 and 2). This allows CFP1 to recognize actively transcribed CGI-associated genes that have non-methylated DNA and elevated H3K4me3 (Figures 2 and 3). Importantly, CFP1-based targeting, as opposed to co-transcriptional recruitment, predominates in defining occupancy of the SET1A complex at target sites (Figure 4). An inability of CFP1 to engage in multivalent interactions with CGI chromatin leads to reductions in H3K4me3 at gene promoters (Figure 5), and loss of CFP1 leads predominantly to reductions in transcription at CFP1-occupied genes (Figure 6). Together, this reveals a central role for CFP1 in recruiting the SET1A complex to shape the promoter-associated epigenome and regulate gene expression.

The mechanisms by which chromatin-modifying factors bind to chromatin and identify their target sites represent a major conceptual gap in our understanding of how the epigenome is formed and regulated. To study this, FRAP can be used to capture dynamic chromatin interactions that are often too rapid to be effectively observed by crosslinking and ChIP (Schmiedeberg et al., 2009), but has limited spatial resolution with respect to the genome. Conversely, ChIP captures more stable binding events with high spatial resolution, even when these represent a small fraction of the total protein molecules in the nucleus. Therefore, combining these approaches, as we have done here, can reveal dynamics and chromatin-binding characteristics across a wide spectrum of temporal and spatial resolutions. Interestingly, our FRAP analysis revealed that CFP1 is a highly dynamic protein and that these dynamics are predominantly driven by inclusion in the SET1 complex. We propose that these dynamics are dictated by low affinity, widespread, and non-specific interactions that the SET1 complex makes with chromatin, which are not effectively captured by ChIP. A mutant form of CFP1 that does not interact with SET1 binds more effectively to CGIs, presumably because it no longer engages in other SET1-dependent interactions with the genome. Conversely, mutation of the CXXC/PHD domains abrogates more stable binding to CGIs, as is evident from loss of ChIP-seq signal at these regions, yet this contributes modestly to FRAP dynamics. This suggests that a small proportion of the total pool of CFP1 molecules in the cell is stably bound to CGIs at any one time. In other

(C) $^{1}H,^{15}N$ heteronuclear single quantum coherence titration experiments using the CFP1 PHD domain and an H3K4me3 peptide.
(D) As in (C) for an unmodified H3K4me0 peptide.
(E–G) Measurements using intrinsic fluorescence spectroscopy for the PHD domain binding to (E) H3K4me3, (F) H3K4me2, and (G) H3K4me1.
(H) A table illustrating the quantitative measurements of binding affinities for the CFP1 PHD domain bound to H3K4 substrates, as determined by intrinsic fluorescence spectroscopy and NMR spectroscopy.

OPEN ACCESS
CellPress

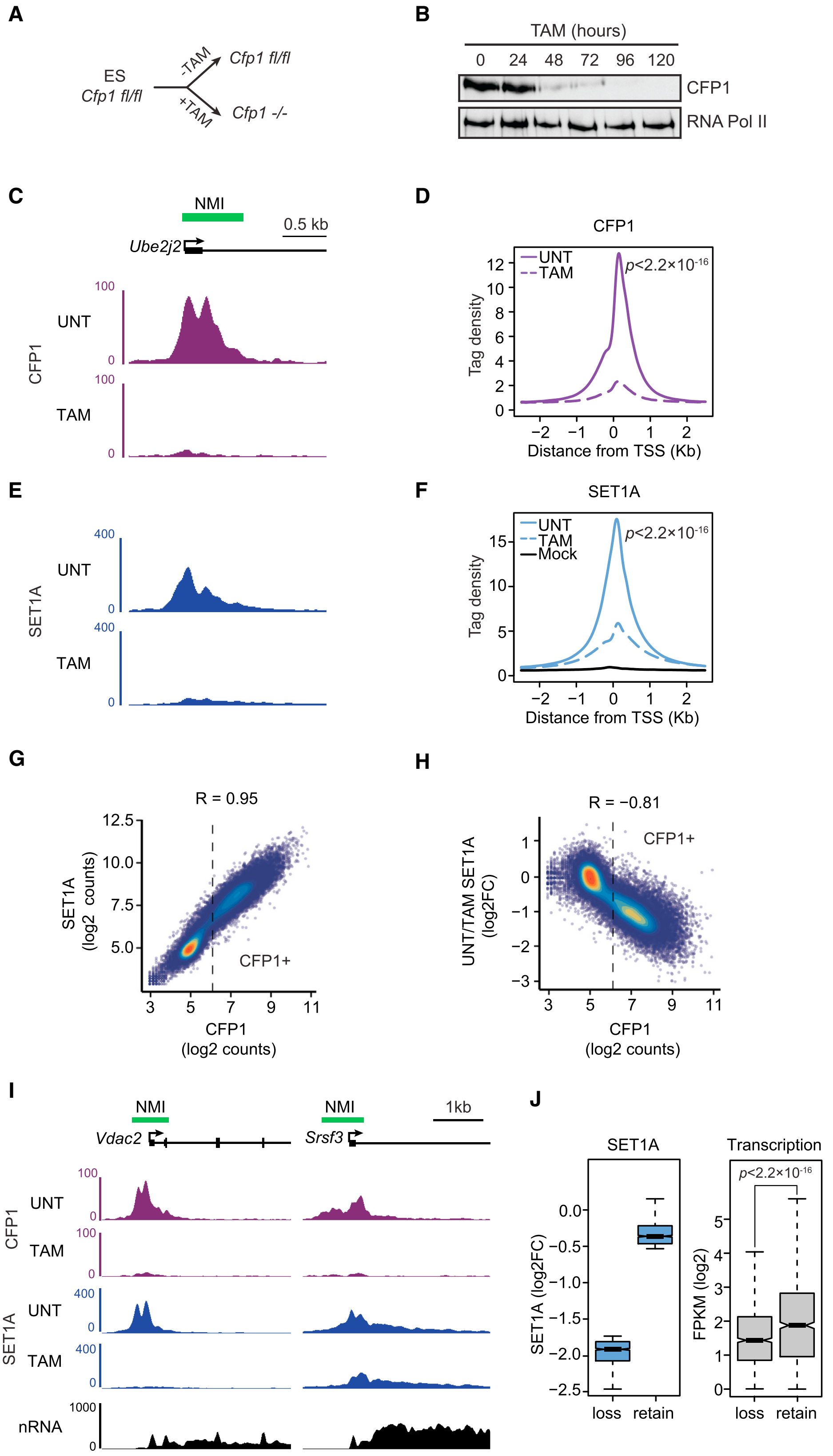

(legend on next page)

words, CFP1 as part of the SET1 complex appears to engage in two general modes of chromatin binding: one that is dynamic, possibly widespread, and SET1 dependent, and a second that is more stable, localized to CGIs, and reliant on multivalent CXXC/PHD domain-dependent interactions with CGI chromatin. It is unclear whether the dynamic pool of CFP1/SET1 observed in FRAP is of functional relevance, but it is possible that this could represent SET1 complex search mechanisms or non-CGI localized processes that the SET1 complex engages in. To further dissect these binding features, new live-cell single molecule approaches (Chen et al., 2014) will be required to track individual CFP1/SET1 molecules and discover how they navigate the nucleus to identify their target sites.

SET1 complexes are thought to play a prominent role in depositing all three H3K4 methylation states (Bledau et al., 2014), yet removal of CFP1 leads primarily to a reduction in H3K4me3 (Figure 5) (Clouaire et al., 2012; Tate et al., 2009). Notably, deletion of the budding yeast ortholog of CFP1, Spp1, also results in a predominant loss of H3K4me3 (Kim et al., 2013; Schlichter and Cairns, 2005). This suggests that regulating the transition to H3K4me3 is an evolutionarily conserved function of CFP1/Spp1, and that deposition of lower H3K4 methylation states by the SET1 complex can occur independently of these factors. It has been proposed that H3K4me3 functions to create transcriptionally permissive chromatin at gene promoters (Voigt et al., 2013). Interestingly, however, we identified only a subset of genes that have reduced expression in the absence of CFP1 (Figure 6), despite global reductions in H3K4me3 at actively transcribed genes (Figure 5). Furthermore, the level of H3K4me3 reduction did not correlate with the effects on transcription, suggesting the level of H3K4me3 and capacity to transcribe are not inextricably linked. Therefore, we favor the possibility that CFP1/SET1 elevates H3K4me3 at actively transcribed genes, with the transcription of some genes being more sensitive to loss of this chromatin modification than others, in agreement with recent observations suggesting that the effects H3K4me3 has on gene expression are context dependent (Cano-Rodriguez et al., 2016). The sensitivity of individual genes to the loss of CFP1/SET1 activity may be related to the nature of their transcriptional inputs. Indeed, we observe that more moderately, as opposed to highly, transcribed genes appear to be misregulated in the absence of CFP1 (Figures 6A and 6B). A possible explanation for this may be that moderately expressed genes are subject to weak activation signals, meaning that chromatin features have more of an influence on transcriptional output. At such genes, loss of CFP1/SET1 activity and the formation of H3K4me3-depleted chromatin may create a greater barrier to transcription. In contrast, at more highly expressed CFP1 target genes, stronger activation signals may render chromatin features less influential.

Genome-wide studies have revealed that individual CGIs generally exist in one of two chromatin states: either having high levels of H3K4me3 and being actively transcribed or being lowly or non-transcribed and having repressive Polycomb group protein (PcG)-associated histone modifications (Blackledge et al., 2015). Given that H3K4 methyltransferase and PcG protein complexes contain CGI-binding domains, we and others have previously proposed that these opposing systems may dynamically sample CGIs in order to respond to the transcriptional state of the associated gene and resolve individual gene regulatory elements into transcriptionally permissive or repressive chromatin states (Blackledge et al., 2015; Deaton and Bird, 2011; Klose et al., 2013; Steffen and Ringrose, 2014; Voigt et al., 2013). Although feedback mechanisms inherent to the PcG protein complexes appear to be sufficient to achieve repressive chromatin states in the absence of gene transcription (Blackledge et al., 2014; Riising et al., 2014), the mechanisms leading to the recruitment and stabilization of H3K4 methyltransferases at actively transcribed genes have remained elusive. Our new observations detailing SET1 targeting in vivo suggest that CFP1 guides SET1A to CGIs that have non-methylated DNA and H3K4me3 to ensure formation of the H3K4me3-predominating state and support normal gene expression.

Although our observations provide a potentially simple explanation for how the CFP1/SET1 complex regulates H3K4me3 at actively transcribed CGI-associated genes, it remains less clear how the H3K4me3-predominating state would be initiated in the first place, given that the CFP1/SET1 complex must recognize pre-existing H3K4me3. One possibility is that the CFP1/SET1 complex binds to CGIs where the MLL1/2 complexes have

Figure 4. CFP1 Is the Central Determinant in SET1A Occupancy on Chromatin

(A) A schematic illustrating the *Cfp1*$^{fl/fl}$ mouse ESC model, in which the addition of tamoxifen (TAM) leads to deletion of CFP1.

(B) Western blot analysis of CFP1 following a time course of TAM treatment.

(C) A genomic snapshot illustrating CFP1 ChIP-seq signal at the *Ube2j2* gene in untreated (upper panel) and tamoxifen-treated cells (lower panel).

(D) Metaplot analysis of CFP1 ChIP-seq signal at CFP1-bound NMI-associated TSSs in untreated (solid line) and tamoxifen-treated cells (dashed line). p values denote statistical significant calculated by a Wilcoxon signed rank test comparing read counts across the represented interval in UNT versus TAM.

(E) A genomic snapshot illustrating SET1A ChIP-seq signal at the at *Ube2j2* gene in untreated (upper panel) and tamoxifen-treated *Cfp1*$^{fl/fl}$ ESCs (lower panel).

(F) Metaplot analysis of T7-SET1A ChIP-seq signal at CFP1-bound NMI-associated TSSs in untreated (solid blue line) and tamoxifen-treated *Cfp1*$^{fl/fl}$ ESCs (dashed blue line). The solid black line illustrates ChIP-seq signal for the T7 antibody in an untagged cell line (Mock). p values denote statistical significance calculated by a Wilcoxon signed rank test comparing read counts across the represented interval in UNT versus TAM.

(G) A scatterplot of the SET1A and CFP1 ChIP-seq signal at TSSs. R value indicates Spearman rank correlation. Genes right of the dashed line correspond to CFP1-bound (CFP1+) sites.

(H) A scatterplot of the log2-fold change in SET1A ChIP-seq signal compared to CFP1 ChIP-seq signal at TSSs. R value indicates Spearman rank correlation. Genes right of the dotted line correspond to CFP1-bound (CFP1+) sites.

(I) Genomic snapshots illustrating a gene where SET1A ChIP-seq signal is lost following removal of CFP1 (left panel) and a gene that is more highly transcribed and retains some SET1A (right panel) following removal of CFP1.

(J) Boxplots illustrating the log2-fold change in SET1A ChIP-seq signal (left panel) and expression level based on 4SU-RNA-seq (right panel) of the top 10% of genes that lose or retain SET1A. Boxes show IQR and whiskers extend by 1.5 × IQR. The p value denotes statistical significance calculated by the Mann-Whitney test.

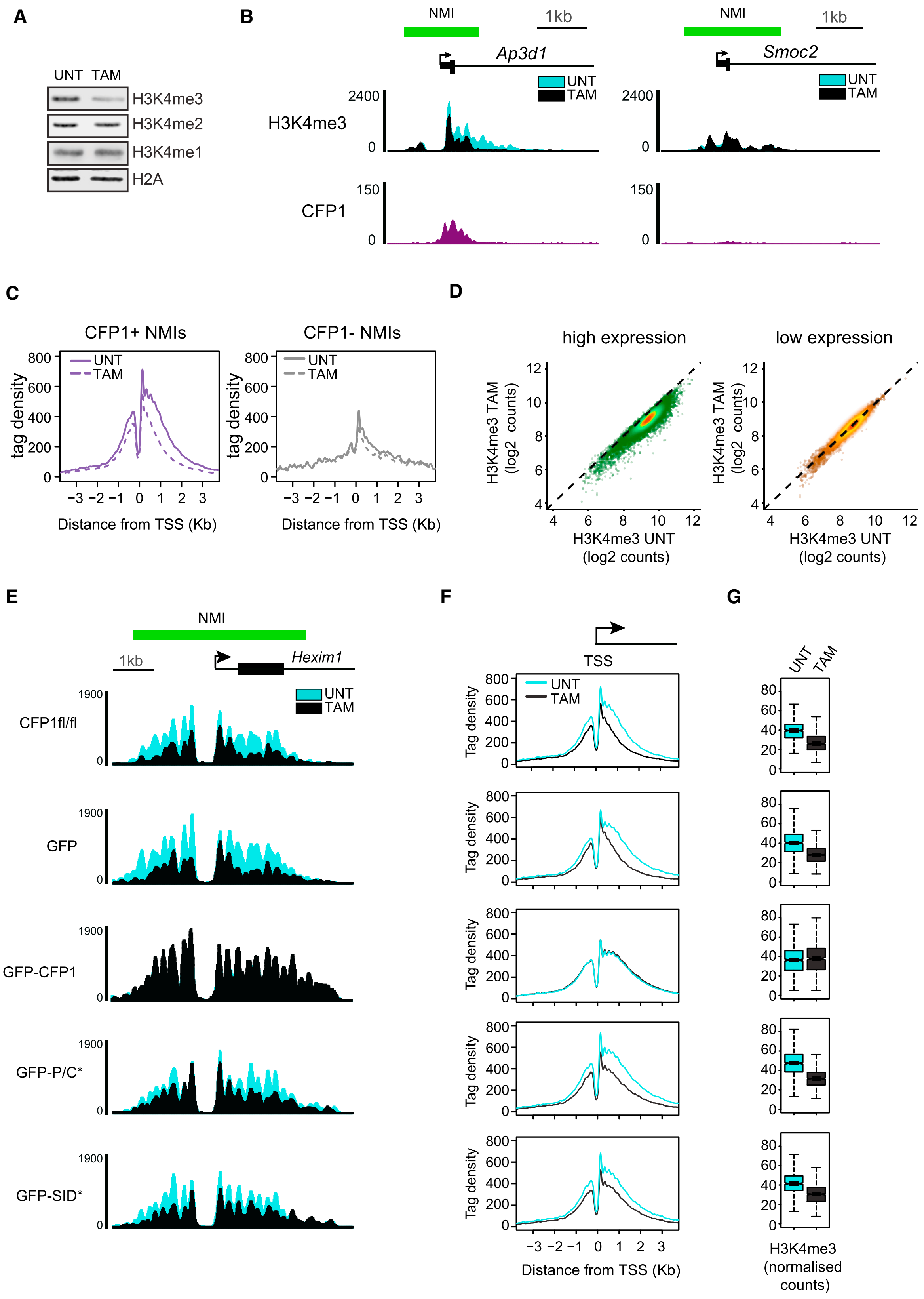

(legend on next page)

already initiated low-level deposition of H3K4me3. However, removal of MLL1/2 has little effect on the levels of H3K4me3 at actively transcribed genes in ESCs, suggesting that the MLL1/2 and SET1 complexes do not act in a simple linear pathway or that MLL1/2 is not required to maintain elevated H3K4me3 once it has been initiated (Denissov et al., 2014; Hu et al., 2013b). Alternatively, the process of initiating gene transcription could result in transient co-transcriptional recruitment of the SET1 complex to seed low levels of H3K4me3 at actively transcribed sites and provide a signal that stabilizes multivalent binding by CFP1/SET1. In support of the latter possibility, we observe residual SET1A occupancy at highly transcribed genes in the absence of CFP1 (Figure 4). Furthermore, initiation and formation of the H3K4me3-predominating state appears to be required for the normal expression of some genes (Figure 6). It is therefore tempting to speculate that the SET1 complex could form part of a simple activity-based epigenetic switch, whereby transcription initiation leads to low-level H3K4me3 deposition at CGIs. This could in turn support multivalent binding of the CFP1/SET1 complex, amplification of the H3K4me3-predominating state, and the formation of chromatin that is more permissive to subsequent rounds of transcription. Although speculative in nature, these proposed feedback mechanisms could, in the context of stochastic models of gene transcription (Lenstra et al., 2016), provide a localized form of chromatin-based epigenetic memory to ensure future rounds of gene transcription once the initial decision to transcribe has been made or to achieve new gene expression programs during developmental transitions. Although it is clear that more detailed mechanistic studies are required to examine the relevance of these proposed feedback mechanisms, they could provide a simple explanation for how genes transition into and maintain the H3K4me3-predominating state to sustain transcription.

EXPERIMENTAL PROCEDURES

FRAP

FRAP experiments were performed on an UltraView spinning disk microscope (Perkin Elmer) equipped with an EM-CCD camera (Hamamatsu) using a 60x/1.4NA oil objective. 50 pre-bleach and 1,000 post-bleach images were captured at a rate of 8 frames per second (fps) (Figures 1B and C) after bleaching a circular diffraction limited spot of ~2.5 μm diameter using a 488-nm laser line at 100% transmission. Alternatively, to capture the rapid recovery of the P/C/S* mutant effectively, we used an acquisition rate of 13 fps (Figures 1E and F). FRAP curves were calculated in MATLAB, normalizing for the initial conditions (brightness of the cell and brightness of the spot) and corrected for acquisition photobleaching over time (Mueller et al., 2012). Half recovery times ($t_{1/2}$) were calculated using a biexponential fit. Briefly, this involved deriving $t_{1/2}$ values from individual cells (Figures S1F and S1G) and then collecting the distribution of $t_{1/2}$ values across biological triplicates for the same transgene (Figures S1H and S1I). To compare the dynamics of individual GFP-CFP1 transgenes, a Student's t test was then used to calculate the probability (*p*) that there was no difference between the wild-type and mutant versions of CFP1.

NMR Titrations of Histone Peptides

The ^{1}H,^{15}N HSQC spectra of 0.1–0.2 mM uniformly ^{15}N-labeled CFP1 PHD finger in 20 mM Tris-HCl buffer, pH 6.8, 100 mM NaCl, 2.5 mM DTT, and 7% D_2O were collected on a Varian INOVA 600 MHz spectrometer. Spectra were recorded at 298K using 1,024 × 128 increments, and a spectral width of 8,820 × 1,974 Hz in the ^{1}H and ^{15}N dimensions, respectively. The binding was characterized by monitoring chemical shift changes as histone tail peptides (synthesized by the University of Colorado Denver Peptide Core Facility) were added stepwise. The dissociation constants (K_ds) were determined as described in the Supplemental Information.

Fluorescence Spectroscopy

Spectra were recorded at 25°C on a Fluoromax-3 spectrofluorometer (HORIBA). The samples containing the CFP1 PHD finger in 20 mM Tris-HCl buffer, pH 6.8, 100 mM NaCl, and 2.5 mM DTT and progressively increasing concentrations of the histone peptide were excited at 280 nm. Emission spectra were recorded over a range of wavelengths between 320 and 380 nm, with a 1-nm step size and a 1-s integration time and averaged over 3 scans. The K_d values were determined as described in the Supplemental Information.

ChIP and ChIP-Seq

ChIP was performed as described previously (Farcas et al., 2012), with minor modifications (see Supplemental Experimental Procedures). Sequencing libraries were prepared with the NEBNext Ultra DNA Library Prep Kit for Illumina and sequenced on either an Illumina HiSeq2500 or NextSeq500.

Calibrated Native ChIP-Seq

ChIP sequencing for H3K4me3 in ESCs was performed using a previously described calibrated native ChIP-seq approach (Rose et al., 2016), in which untreated or tamoxifen-treated *Cfp1*$^{fl/fl}$ cells were spiked with a fixed number of *Drosophila* SG4 cells (see Supplemental Experimental Procedures). Libraries were prepared with NEBNext Ultra DNA Library Prep Kit for Illumina and quantified by qPCR using KAPA Illumina DNA standards as reference. Libraries were sequenced on an Illumina NextSeq500.

4sU RNA-Seq

Cells were treated with 500 μM 4-thiouridine (4sU) for 20 min and then RNA was isolated by TRIZOL (Thermo Fisher Scientific) extraction. RNA was incubated with Biotin-HPDP and biotinylated RNA was captured with μMACS streptavidin beads (Miltenyi). Biotinylated RNA was depleted of ribosomal RNAs using the Low Input RiboMinus Eukaryote System v2 kit (Thermo Fisher Scientific).

Figure 5. CFP1 Exploits Multivalent Interactions with CpG Island Chromatin to Shape H3K4me3

(A) Western blot analysis of bulk H3K4me in untreated (UNT) and TAM-treated *Cfp1*$^{fl/fl}$ mouse ESCs.

(B) Genomic snapshot illustrating H3K4me3 ChIP-seq signal at the *Ap3d1* NMI, which is bound by CFP1 (left panel) and the *Smoc2* NMI that is not bound by CFP1 (right panel). The H3K4me3 ChIP-seq signal without tamoxifen treatment is represented in blue (UNT) and the signal with tamoxifen treatment is represented in black (TAM) in the overlay.

(C) H3K4me3 signal around CFP1+ (left) and CFP1− (right) NMI-associated TSSs in untreated (solid line) and tamoxifen-treated cells (dashed line).

(D) A scatterplot illustrating that highly (left panel) but not lowly (right panel) expressed genes lose H3K4me3 at their TSS following tamoxifen treatment. The scatterplots correspond to non-divergent genes with an H3K4me3 peak overlapping their TSS, and a gene was considered to be lowly expressed if it had less than −2.5 log2 FPKM nuclear RNA seq signal over the gene body.

(E) Genomic snapshot illustrating H3K4me3 ChIP-seq signal at the *Hexim1* NMI in UNT and TAM-treated cells. The UNT and TAM-treated samples are colored blue and black in the overlay, respectively. The upper panel corresponds to the parental *Cfp1*$^{fl/fl}$ line, with the lower panels corresponding to the parental line rescued with at GFP, GFP-CFP1, GFP-P/C*, or GFP-SID* transgenes.

(F) Metaplot and boxplot analysis of the H3K4me3 signal at TSSs as described in (C) for cell lines described in (E) without and with tamoxifen treatment.

(G) Boxplot analysis of H3K4me3 signal at CFP1+ NMI-associated TSSs +/- 1 kb. Boxes show IQR and whiskers extend by 1.5 × IQR.

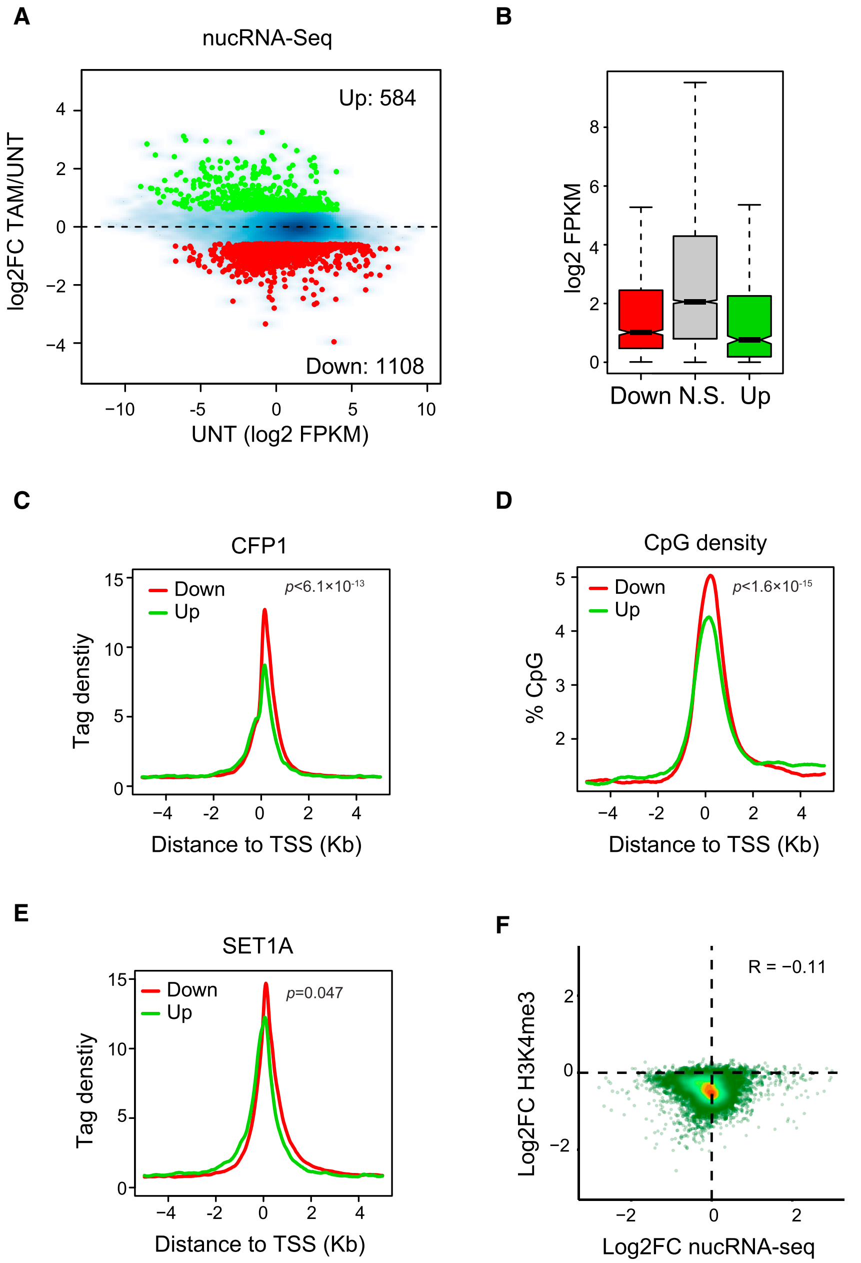

Figure 6. Loss of CFP1 Leads to Widespread Effects on Gene Expression

(A) An MA plot showing log2-fold change in the nuclear RNA-seq signal of CFP1-bound NMI-associated genes in UNT and TAM-treated *Cfp1*$^{fl/fl}$ cells. Red and green points depict significantly downregulated (1,108) and upregulated genes (584) that change in expression by more than 1.5-fold.

(B) Boxplots indicating the expression of genes that are downregulated (red), not significantly (N.S.) changing (gray), or upregulated (green).

(C–E) Mean distribution of CFP1 ChIP-seq signal (C), CpG density (D), and SET1A ChIP-seq signal (E) around TSSs of downregulated (red) and upregulated (green) genes. p values denote statistical significance calculated by Mann-Whitney test comparing ChIP-seq read counts across a 200-bp interval flanking the TSS in downregulated versus upregulated genes.

(F) Correlation density plot of changes in gene expression (nucRNA-seq) and H3K4me3 at TSSs of CFP1-bound NMI genes. Only genes whose TSSs overlap an H3K4me3 peak and do not have a divergent TSS within 2 kb were considered. R value indicates Spearman rank correlation.

cDNA libraries were prepared using the NEBNext Ultra Directional RNA Library Prep Kit and subjected to sequencing on the Illumina NextSeq500 platform.

Quantitative Nuclear RNA-Seq

Cfp1$^{fl/fl}$ ESCs were cultured for 96 hr in the presence or absence of 4-OHT. For each condition, 4 × 10^6 cells were spiked with 1 × 10^6 *Drosphila* SG4 cells. Nuclei were extracted, and an aliquot of nuclei corresponding to 4 × 10^5 cells was collected for genomic DNA extraction, whereas the remaining nuclei were subject to conventional RNA extraction using TRIZOL (Thermo Fisher Scientific). Nuclear RNA was depleted of ribosomal RNAs using the NEBNext rRNA Depletion kit, and cDNA libraries were prepared using the NEBNext Ultra Directional RNA Library Prep Kit. In parallel, genomic DNA (gDNA) was used to prepare "input" DNA libraries using the NEBNext Ultra DNA Library Prep Kit. Nuclear RNA (nucRNA) and gDNA libraries were sequenced on the Illumina NextSeq500 platform.

ACCESSION NUMBERS

The accession number for the datasets reported in this paper is GEO: GSE93538.

SUPPLEMENTAL INFORMATION

Supplemental Information includes Supplemental Experimental Procedures and six figures and can be found with this article online at http://dx.doi.org/10.1016/j.celrep.2017.08.030.

AUTHOR CONTRIBUTIONS

D.A.B. (ORCID ID 0000-0001-7531-8230), V.D.C. (ORCID ID 0000-0002-2920-1274), A.F., J.A., S.I., L.S., T.G.K., H.K., and R.J.K. conceived the project. T.G.K., S.K., T.G.K., H.K., and R.J.K. secured funding. D.A.B., V.C.D., A.F., J.A., S.I., N.P.B., E.D., M.N., M.M., H.K.L. (ORCID ID 0000-0002-5694-0398), A.H.T., and H.W.K. (ORCID ID 0000-0001-5972-8926) generated reagents and performed the experiments. D.A.B., V.D.C., A.F., N.P.B., S.K., L.S., T.G.K., H.K., and R.J.K. wrote and edited the manuscript.

ACKNOWLEDGMENTS

Work in the Klose lab is supported by the Wellcome Trust (098024/Z/11/Z), the Lister Institute of Preventive Medicine, Exeter College University of Oxford, EMBO, and the European Research Council (681440). D.A.B. was supported by a Wellcome Trust PhD studentship, and A.F. is supported by a Sir Henry Wellcome Postdoctoral Fellowship (110286/Z/15/Z). The Kutateladze lab was supported by NIH R01 GM106416 and GM100907, and the Kriaucionis lab was supported by the Ludwig Institute for Cancer Research and BBSRC BB/M001873/1. We would like to thank Ed Hookway and Udo Oppermann for sequencing support on the NextSeq500 at the Botnar sequencing facility in Oxford and the Oxford Genomics Centre at the Wellcome Trust Centre for Human Genetics for sequencing on the HiSeq platform. Microscopy was supported by the Micron Oxford Advanced Bioimaging Unit funded by a Wellcome Trust Strategic Award (no. 091911).

Received: March 14, 2017
Revised: July 19, 2017
Accepted: August 6, 2017
Published: September 5, 2017

REFERENCES

Andreu-Vieyra, C.V., Chen, R., Agno, J.E., Glaser, S., Anastassiadis, K., Stewart, A.F., and Matzuk, M.M. (2010). MLL2 is required in oocytes for bulk histone 3 lysine 4 trimethylation and transcriptional silencing. PLoS Biol. *8*.

Ardehali, M.B., Mei, A., Zobeck, K.L., Caron, M., Lis, J.T., and Kusch, T. (2011). Drosophila Set1 is the major histone H3 lysine 4 trimethyltransferase with role in transcription. EMBO J. *30*, 2817–2828.

Austenaa, L.M., Barozzi, I., Simonatto, M., Masella, S., Della Chiara, G., Ghisletti, S., Curina, A., de Wit, E., Bouwman, B.A., de Pretis, S., et al. (2015). Transcription of mammalian cis-regulatory elements is restrained by actively enforced early termination. Mol. Cell *60*, 460–474.

Bernstein, B.E., Kamal, M., Lindblad-Toh, K., Bekiranov, S., Bailey, D.K., Huebert, D.J., McMahon, S., Karlsson, E.K., Kulbokas, E.J., 3rd, Gingeras, T.R., et al. (2005). Genomic maps and comparative analysis of histone modifications in human and mouse. Cell *120*, 169–181.

Birke, M., Schreiner, S., García-Cuéllar, M.P., Mahr, K., Titgemeyer, F., and Slany, R.K. (2002). The MT domain of the proto-oncoprotein MLL binds to CpG-containing DNA and discriminates against methylation. Nucleic Acids Res. *30*, 958–965.

Blackledge, N.P., Zhou, J.C., Tolstorukov, M.Y., Farcas, A.M., Park, P.J., and Klose, R.J. (2010). CpG islands recruit a histone H3 lysine 36 demethylase. Mol. Cell *38*, 179–190.

Blackledge, N.P., Long, H.K., Zhou, J.C., Kriaucionis, S., Patient, R., and Klose, R.J. (2012). Bio-CAP: a versatile and highly sensitive technique to purify and characterise regions of non-methylated DNA. Nucleic Acids Res. *40*, e32.

Blackledge, N.P., Farcas, A.M., Kondo, T., King, H.W., McGouran, J.F., Hanssen, L.L., Ito, S., Cooper, S., Kondo, K., Koseki, Y., et al. (2014). Variant PRC1 complex-dependent H2A ubiquitylation drives PRC2 recruitment and polycomb domain formation. Cell *157*, 1445–1459.

Blackledge, N.P., Rose, N.R., and Klose, R.J. (2015). Targeting Polycomb systems to regulate gene expression: modifications to a complex story. Nat. Rev. Mol. Cell Biol. *16*, 643–649.

Bledau, A.S., Schmidt, K., Neumann, K., Hill, U., Ciotta, G., Gupta, A., Torres, D.C., Fu, J., Kranz, A., Stewart, A.F., et al. (2014). The H3K4 methyltransferase Setd1a is first required at the epiblast stage, whereas Setd1b becomes essential after gastrulation. Development *141*, 1022–1035.

Cano-Rodriguez, D., Gjaltema, R.A., Jilderda, L.J., Jellema, P., Dokter-Fokkens, J., Ruiters, M.H., and Rots, M.G. (2016). Writing of H3K4Me3 overcomes epigenetic silencing in a sustained but context-dependent manner. Nat. Commun. *7*, 12284.

Carlone, D.L., Lee, J.H., Young, S.R., Dobrota, E., Butler, J.S., Ruiz, J., and Skalnik, D.G. (2005). Reduced genomic cytosine methylation and defective cellular differentiation in embryonic stem cells lacking CpG binding protein. Mol. Cell. Biol. *25*, 4881–4891.

Chen, J., Zhang, Z., Li, L., Chen, B.C., Revyakin, A., Hajj, B., Legant, W., Dahan, M., Lionnet, T., Betzig, E., et al. (2014). Single-molecule dynamics of enhanceosome assembly in embryonic stem cells. Cell *156*, 1274–1285.

Clouaire, T., Webb, S., Skene, P., Illingworth, R., Kerr, A., Andrews, R., Lee, J.H., Skalnik, D., and Bird, A. (2012). Cfp1 integrates both CpG content and gene activity for accurate H3K4me3 deposition in embryonic stem cells. Genes Dev. *26*, 1714–1728.

Clouaire, T., Webb, S., and Bird, A. (2014). Cfp1 is required for gene expression-dependent H3K4 trimethylation and H3K9 acetylation in embryonic stem cells. Genome Biol. *15*, 451.

Deaton, A.M., and Bird, A. (2011). CpG islands and the regulation of transcription. Genes Dev. *25*, 1010–1022.

Denissov, S., Hofemeister, H., Marks, H., Kranz, A., Ciotta, G., Singh, S., Anastassiadis, K., Stunnenberg, H.G., and Stewart, A.F. (2014). Mll2 is required for H3K4 trimethylation on bivalent promoters in embryonic stem cells, whereas Mll1 is redundant. Development *141*, 526–537.

Eberl, H.C., Spruijt, C.G., Kelstrup, C.D., Vermeulen, M., and Mann, M. (2013). A map of general and specialized chromatin readers in mouse tissues generated by label-free interaction proteomics. Mol. Cell *49*, 368–378.

ENCODE Project Consortium (2012). An integrated encyclopedia of DNA elements in the human genome. Nature *489*, 57–74.

Farcas, A.M., Blackledge, N.P., Sudbery, I., Long, H.K., McGouran, J.F., Rose, N.R., Lee, S., Sims, D., Cerase, A., Sheahan, T.W., et al. (2012). KDM2B links the Polycomb Repressive Complex 1 (PRC1) to recognition of CpG islands. eLife *1*, e00205.

Hallson, G., Hollebakken, R.E., Li, T., Syrzycka, M., Kim, I., Cotsworth, S., Fitzpatrick, K.A., Sinclair, D.A., and Honda, B.M. (2012). dSet1 is the main H3K4 di- and tri-methyltransferase throughout Drosophila development. Genetics *190*, 91–100.

Heintzman, N.D., Stuart, R.K., Hon, G., Fu, Y., Ching, C.W., Hawkins, R.D., Barrera, L.O., Van Calcar, S., Qu, C., Ching, K.A., et al. (2007). Distinct and predictive chromatin signatures of transcriptional promoters and enhancers in the human genome. Nat. Genet. *39*, 311–318.

Heintzman, N.D., Hon, G.C., Hawkins, R.D., Kheradpour, P., Stark, A., Harp, L.F., Ye, Z., Lee, L.K., Stuart, R.K., Ching, C.W., et al. (2009). Histone modifications at human enhancers reflect global cell-type-specific gene expression. Nature *459*, 108–112.

Hu, D., Gao, X., Morgan, M.A., Herz, H.M., Smith, E.R., and Shilatifard, A. (2013a). The MLL3/MLL4 branches of the COMPASS family function as major histone H3K4 monomethylases at enhancers. Mol. Cell. Biol. *33*, 4745–4754.

Hu, D., Garruss, A.S., Gao, X., Morgan, M.A., Cook, M., Smith, E.R., and Shilatifard, A. (2013b). The Mll2 branch of the COMPASS family regulates bivalent promoters in mouse embryonic stem cells. Nat. Struct. Mol. Biol. *20*, 1093–1097.

Hu, B., Petela, N., Kurze, A., Chan, K.L., Chapard, C., and Nasmyth, K. (2015). Biological chromodynamics: a general method for measuring protein occupancy across the genome by calibrating ChIP-seq. Nucleic Acids Res. *43*, e132.

Illingworth, R.S., Gruenewald-Schneider, U., Webb, S., Kerr, A.R., James, K.D., Turner, D.J., Smith, C., Harrison, D.J., Andrews, R., and Bird, A.P.

(2010). Orphan CpG islands identify numerous conserved promoters in the mammalian genome. PLoS Genet. *6*, e1001134.

Kaikkonen, M.U., Spann, N.J., Heinz, S., Romanoski, C.E., Allison, K.A., Stender, J.D., Chun, H.B., Tough, D.F., Prinjha, R.K., Benner, C., et al. (2013). Remodeling of the enhancer landscape during macrophage activation is coupled to enhancer transcription. Mol. Cell *51*, 310–325.

Kim, J., Kim, J.A., McGinty, R.K., Nguyen, U.T., Muir, T.W., Allis, C.D., and Roeder, R.G. (2013). The n-SET domain of Set1 regulates H2B ubiquitylation-dependent H3K4 methylation. Mol. Cell *49*, 1121–1133.

Klose, R.J., Cooper, S., Farcas, A.M., Blackledge, N.P., and Brockdorff, N. (2013). Chromatin sampling–an emerging perspective on targeting polycomb repressor proteins. PLoS Genet. *9*, e1003717.

Kusch, T. (2012). Histone H3 lysine 4 methylation revisited. Transcription *3*, 310–314.

Lauberth, S.M., Nakayama, T., Wu, X., Ferris, A.L., Tang, Z., Hughes, S.H., and Roeder, R.G. (2013). H3K4me3 interactions with TAF3 regulate preinitiation complex assembly and selective gene activation. Cell *152*, 1021–1036.

Lee, J.H., and Skalnik, D.G. (2005). CpG-binding protein (CXXC finger protein 1) is a component of the mammalian Set1 histone H3-Lys4 methyltransferase complex, the analogue of the yeast Set1/COMPASS complex. J. Biol. Chem. *280*, 41725–41731.

Lee, J.H., and Skalnik, D.G. (2008). Wdr82 is a C-terminal domain-binding protein that recruits the Setd1A Histone H3-Lys4 methyltransferase complex to transcription start sites of transcribed human genes. Mol. Cell. Biol. *28*, 609–618.

Lee, J.E., Wang, C., Xu, S., Cho, Y.W., Wang, L., Feng, X., Baldridge, A., Sartorelli, V., Zhuang, L., Peng, W., et al. (2013). H3K4 mono- and di-methyltransferase MLL4 is required for enhancer activation during cell differentiation. eLife *2*, e01503.

Lenstra, T.L., Rodriguez, J., Chen, H., and Larson, D.R. (2016). Transcription dynamics in living cells. Annu. Rev. Biophys. *45*, 25–47.

Levine, M., Cattoglio, C., and Tjian, R. (2014). Looping back to leap forward: transcription enters a new era. Cell *157*, 13–25.

Li, H., Ilin, S., Wang, W., Duncan, E.M., Wysocka, J., Allis, C.D., and Patel, D.J. (2006). Molecular basis for site-specific read-out of histone H3K4me3 by the BPTF PHD finger of NURF. Nature *442*, 91–95.

Long, H.K., Blackledge, N.P., and Klose, R.J. (2013a). ZF-CxxC domain-containing proteins, CpG islands and the chromatin connection. Biochem. Soc. Trans. *41*, 727–740.

Long, H.K., Sims, D., Heger, A., Blackledge, N.P., Kutter, C., Wright, M.L., Grützner, F., Odom, D.T., Patient, R., Ponting, C.P., et al. (2013b). Epigenetic conservation at gene regulatory elements revealed by non-methylated DNA profiling in seven vertebrates. eLife *2*, e00348.

Lorch, Y., LaPointe, J.W., and Kornberg, R.D. (1987). Nucleosomes inhibit the initiation of transcription but allow chain elongation with the displacement of histones. Cell *49*, 203–210.

Mahadevan, J., and Skalnik, D.G. (2016). Efficient differentiation of murine embryonic stem cells requires the binding of CXXC finger protein 1 to DNA or methylated histone H3-Lys4. Gene *594*, 1–9.

Milne, T.A., Briggs, S.D., Brock, H.W., Martin, M.E., Gibbs, D., Allis, C.D., and Hess, J.L. (2002). MLL targets SET domain methyltransferase activity to Hox gene promoters. Mol. Cell *10*, 1107–1117.

Mueller, F., Karpova, T.S., Mazza, D., and McNally, J.G. (2012). Monitoring dynamic binding of chromatin proteins in vivo by fluorescence recovery after photobleaching. Methods Mol. Biol. *833*, 153–176.

Musselman, C.A., Lalonde, M.E., Côté, J., and Kutateladze, T.G. (2012). Perceiving the epigenetic landscape through histone readers. Nat. Struct. Mol. Biol. *19*, 1218–1227.

Ng, H.H., Robert, F., Young, R.A., and Struhl, K. (2003). Targeted recruitment of Set1 histone methylase by elongating Pol II provides a localized mark and memory of recent transcriptional activity. Mol. Cell *11*, 709–719.

Peña, P.V., Davrazou, F., Shi, X., Walter, K.L., Verkhusha, V.V., Gozani, O., Zhao, R., and Kutateladze, T.G. (2006). Molecular mechanism of histone H3K4me3 recognition by plant homeodomain of ING2. Nature *442*, 100–103.

Piunti, A., and Shilatifard, A. (2016). Epigenetic balance of gene expression by Polycomb and COMPASS families. Science *352*, aad9780.

Riising, E.M., Comet, I., Leblanc, B., Wu, X., Johansen, J.V., and Helin, K. (2014). Gene silencing triggers polycomb repressive complex 2 recruitment to CpG islands genome wide. Mol. Cell *55*, 347–360.

Risner, L.E., Kuntimaddi, A., Lokken, A.A., Achille, N.J., Birch, N.W., Schoenfelt, K., Bushweller, J.H., and Zeleznik-Le, N.J. (2013). Functional specificity of CpG DNA-binding CXXC domains in mixed lineage leukemia. J. Biol. Chem. *288*, 29901–29910.

Rose, N.R., King, H.W., Blackledge, N.P., Fursova, N.A., Ember, K.J., Fischer, R., Kessler, B.M., and Klose, R.J. (2016). RYBP stimulates PRC1 to shape chromatin-based communication between Polycomb repressive complexes. eLife *5*.

Schlichter, A., and Cairns, B.R. (2005). Histone trimethylation by Set1 is coordinated by the RRM, autoinhibitory, and catalytic domains. EMBO J. *24*, 1222–1231.

Schmiedeberg, L., Skene, P., Deaton, A., and Bird, A. (2009). A temporal threshold for formaldehyde crosslinking and fixation. PLoS ONE *4*, e4636.

Schneider, R., Bannister, A.J., Myers, F.A., Thorne, A.W., Crane-Robinson, C., and Kouzarides, T. (2004). Histone H3 lysine 4 methylation patterns in higher eukaryotic genes. Nat. Cell Biol. *6*, 73–77.

Shi, X., Hong, T., Walter, K.L., Ewalt, M., Michishita, E., Hung, T., Carney, D., Peña, P., Lan, F., Kaadige, M.R., et al. (2006). ING2 PHD domain links histone H3 lysine 4 methylation to active gene repression. Nature *442*, 96–99.

Shilatifard, A. (2012). The COMPASS family of histone H3K4 methylases: mechanisms of regulation in development and disease pathogenesis. Annu. Rev. Biochem. *81*, 65–95.

Steffen, P.A., and Ringrose, L. (2014). What are memories made of? How Polycomb and Trithorax proteins mediate epigenetic memory. Nat. Rev. Mol. Cell Biol. *15*, 340–356.

Tate, C.M., Lee, J.H., and Skalnik, D.G. (2009). CXXC finger protein 1 contains redundant functional domains that support embryonic stem cell cytosine methylation, histone methylation, and differentiation. Mol. Cell. Biol. *29*, 3817–3831.

Thomson, J.P., Skene, P.J., Selfridge, J., Clouaire, T., Guy, J., Webb, S., Kerr, A.R., Deaton, A., Andrews, R., James, K.D., et al. (2010). CpG islands influence chromatin structure via the CpG-binding protein Cfp1. Nature *464*, 1082–1086.

van Nuland, R., Smits, A.H., Pallaki, P., Jansen, P.W., Vermeulen, M., and Timmers, H.T. (2013). Quantitative dissection and stoichiometry determination of the human SET1/MLL histone methyltransferase complexes. Mol. Cell. Biol. *33*, 2067–2077.

Venkatesh, S., and Workman, J.L. (2015). Histone exchange, chromatin structure and the regulation of transcription. Nat. Rev. Mol. Cell Biol. *16*, 178–189.

Vermeulen, M., Mulder, K.W., Denissov, S., Pijnappel, W.W., van Schaik, F.M., Varier, R.A., Baltissen, M.P., Stunnenberg, H.G., Mann, M., and Timmers, H.T. (2007). Selective anchoring of TFIID to nucleosomes by trimethylation of histone H3 lysine 4. Cell *131*, 58–69.

Voigt, P., Tee, W.W., and Reinberg, D. (2013). A double take on bivalent promoters. Genes Dev. *27*, 1318–1338.

Voo, K.S., Carlone, D.L., Jacobsen, B.M., Flodin, A., and Skalnik, D.G. (2000). Cloning of a mammalian transcriptional activator that binds unmethylated CpG motifs and shares a CXXC domain with DNA methyltransferase, human trithorax, and methyl-CpG binding domain protein 1. Mol. Cell. Biol. *20*, 2108–2121.

Wang, P., Lin, C., Smith, E.R., Guo, H., Sanderson, B.W., Wu, M., Gogol, M., Alexander, T., Seidel, C., Wiedemann, L.M., et al. (2009). Global analysis of H3K4 methylation defines MLL family member targets and points to a role

for MLL1-mediated H3K4 methylation in the regulation of transcriptional initiation by RNA polymerase II. Mol. Cell. Biol. *29*, 6074–6085.

Wu, M., Wang, P.F., Lee, J.S., Martin-Brown, S., Florens, L., Washburn, M., and Shilatifard, A. (2008). Molecular regulation of H3K4 trimethylation by Wdr82, a component of human Set1/COMPASS. Mol. Cell. Biol. *28*, 7337–7344.

Wysocka, J., Swigut, T., Xiao, H., Milne, T.A., Kwon, S.Y., Landry, J., Kauer, M., Tackett, A.J., Chait, B.T., Badenhorst, P., et al. (2006). A PHD finger of NURF couples histone H3 lysine 4 trimethylation with chromatin remodelling. Nature *442*, 86–90.

Xu, C., Bian, C., Lam, R., Dong, A., and Min, J. (2011). The structural basis for selective binding of non-methylated CpG islands by the CFP1 CXXC domain. Nat. Commun. *2*, 227.

Cell Reports, Volume *20*

Supplemental Information

The SET1 Complex Selects Actively Transcribed Target Genes via Multivalent Interaction with CpG Island Chromatin

David A. Brown, Vincenzo Di Cerbo, Angelika Feldmann, Jaewoo Ahn, Shinsuke Ito, Neil P. Blackledge, Manabu Nakayama, Michael McClellan, Emilia Dimitrova, Anne H. Turberfield, Hannah K. Long, Hamish W. King, Skirmantas Kriaucionis, Lothar Schermelleh, Tatiana G. Kutateladze, Haruhiko Koseki, and Robert J. Klose

Supplementary Material

Supplementary Figures:

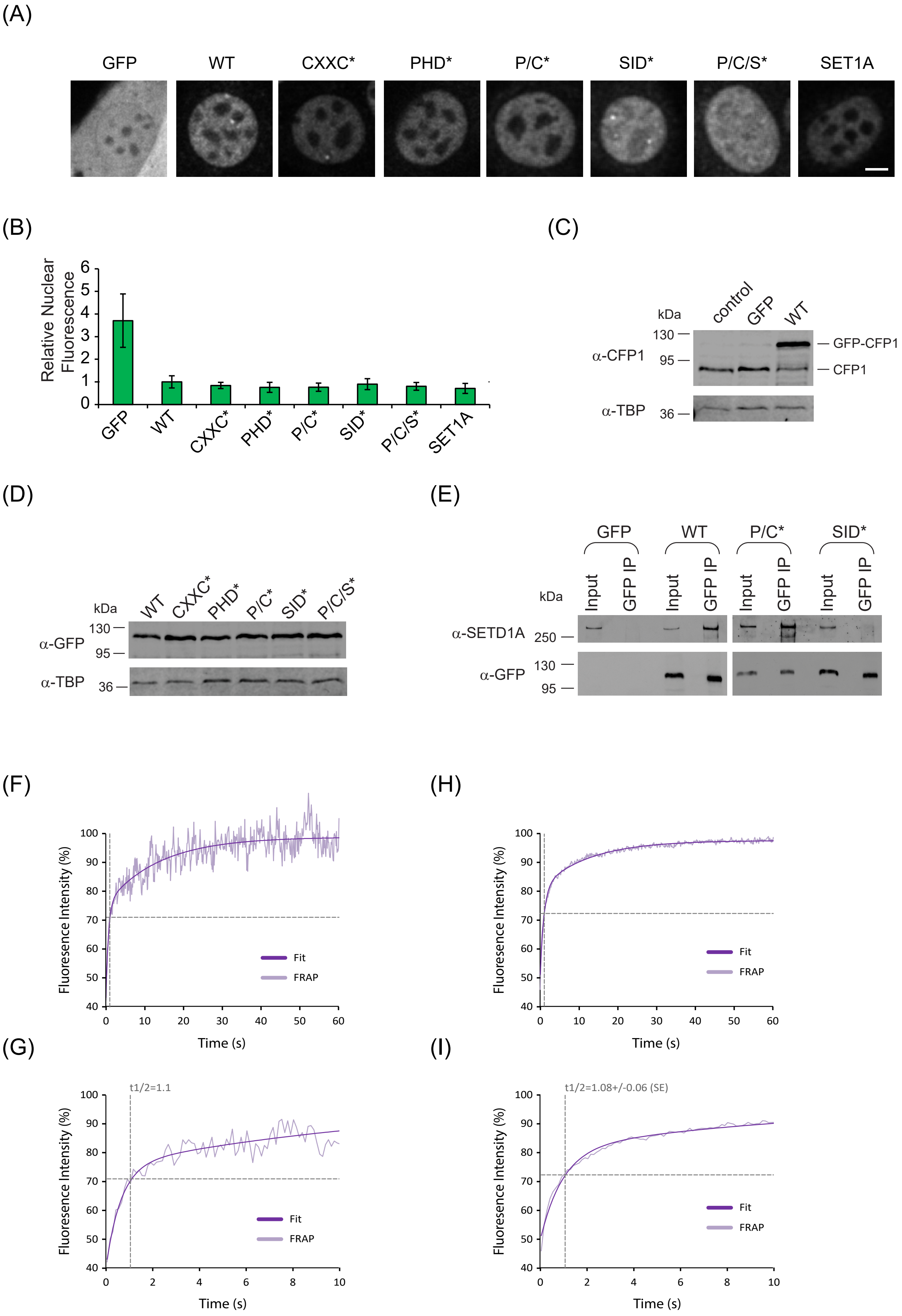

(A)
GFP
WT
CXXC*
PHD*
P/C*
SID*
P/C/S*
SET1A
(B)
Relative Nuclear Fluorescence
6
5
4
3
2
1
0
GFP
WT
CXXC*
PHD*
P/C*
SID*
P/C/S*
SET1A
(C)
kDa
control
GFP
WT
130
95
36
α-CFP1
α-TBP
GFP-CFP1
CFP1
(D)
kDa
WT
CXXC*
PHD*
P/C*
SID*
P/C/S*
130
95
36
α-GFP
α-TBP
(E)
GFP
WT
P/C*
SID*
Input
GFP IP
kDa
α-SETD1A
250
α-GFP
130
95
(F)
(H)
Fluorescence Intensity (%)
Time (s)
Fit
FRAP
(G)
(I)
t1/2=1.1
t1/2=1.08+/-0.06 (SE)

Figure S1

Figure S1- Validation of transgene protein levels and complex formation in C127 cell lines and explanation of FRAP analysis approach used in this study. Related to Figure 1.

(A) Live cell images of stable GFP and GFP fusion protein expression in mouse C127 cells demonstrating nuclear localisation. The scale bar corresponds to 5 µm.
(B) Quantitation of GFP and GFP fusion protein fluorescence in stably expressing C127 cells indicating equivalent protein expression. Error bars correspond to the SEM from 3 biological replicates.
(C) Western blot analysis using a CFP1-specific antibody, indicating that GFP-CFP1 protein levels in WT cell line are comparable to endogenous CFP1 in both C127 control and GFP-only cells. Western blot analysis with a TATA box binding protein (TBP)-specific antibody was used to demonstrate equal loading of nuclear extracts.
(D) Western blot analysis using a GFP-specific antibody, indicating that GFP-CFP1 protein levels in WT cell line are highly similar to all mutant forms of GFP-CFP1 used in C127 FRAP and ChIP-seq experiments. Western blot analysis with a TATA box binding protein (TBP)-specific antibody was used to demonstrate equal loading of nuclear extracts.
(E) Immunoprecipitation with a GFP-specific antibody, using nuclear extract from GFP-only, GFP-CFP1 WT, GFP-CFP1 P/C* and GFP1-CFP1 SID* cell lines. Immunoprecipitated material was subjected to western blot analysis, with GFP- and SETD1A-specific antibodies. As expected, CFP1 WT co-immunoprecipitates with SETD1A. Importantly, mutating the PHD and CXXC domains of CFP1 does not affect the interaction with SETD1A, whereas mutating the SET1 interaction domain abolishes the SETD1A interaction.
(F) An example FRAP recording, and biexponential fit from an individual C127 cell stably expressing WT GFP-CFP1.
(G) The first 10 s of the recovery shown in (F), the estimate of $t_{1/2}$ is given above.
(H) Mean FRAP recovery and biexponential fit for WT GFP-CFP1 measured in 45 cells across three biological replicates.
(I) The first 10 s of the recovery shown in (H), the estimate of $t_{1/2}$ is given above.

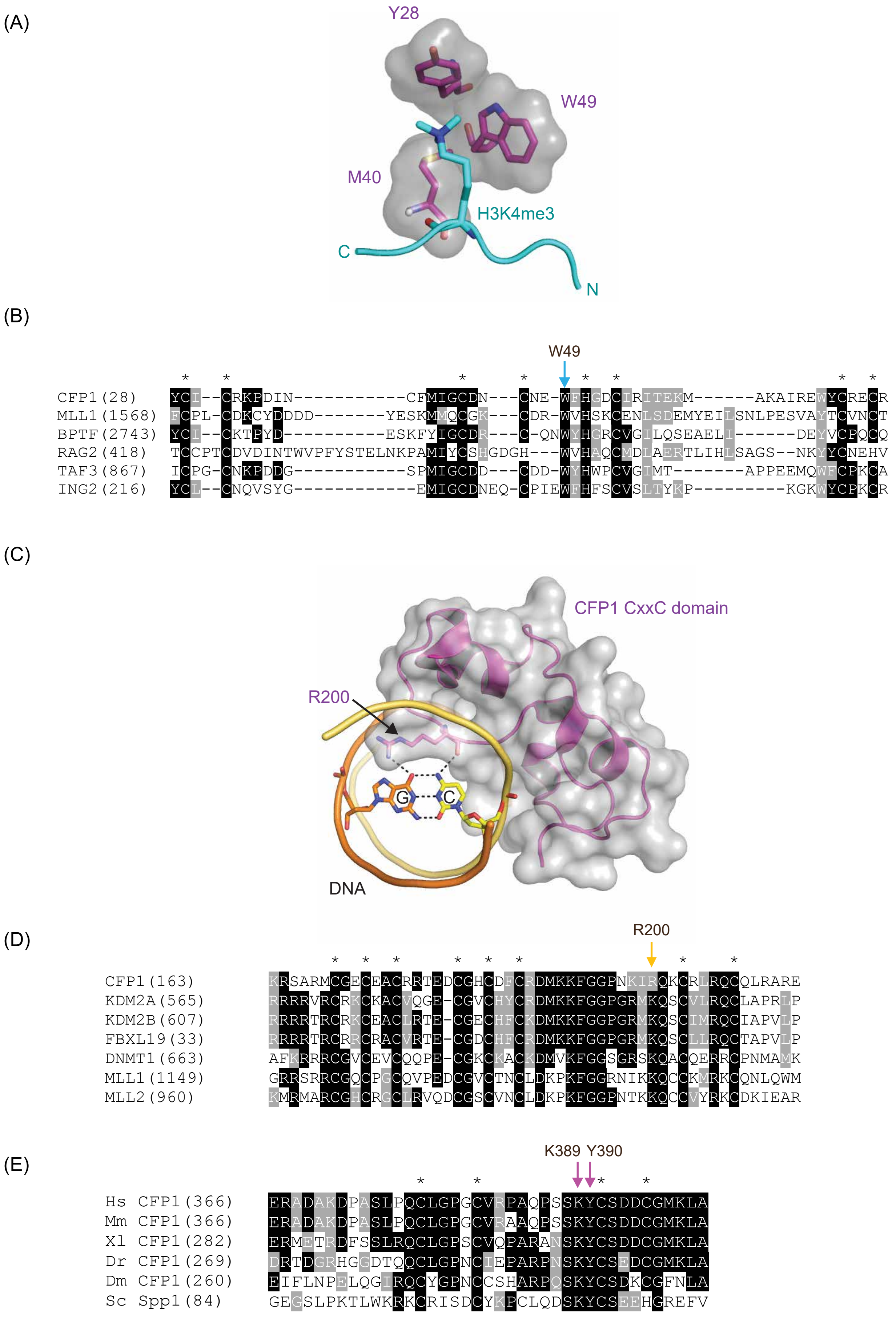

(A)
Y28
W49
M40
H3K4me3
C
N
(B)
W49
CFP1(28) YCI--CRKPDIN-----------CFMIGCDN---CNE-WFHGDCIRITEKM------AKAIREWYCRECR
MLL1(1568) FCPL-CDKCYDDDD--------YESKMMQCGK---CDR-WVHSKCENLSDEMYEILSNLPESVAYTCVNCT
BPTF(2743) YCI--CKTPYD-----------ESKFYIGCDR---C-QNWYHGRCVGILQSEAELI------DEYVCPQCQ
RAG2(418) TCCPTCDVDINTWVPFYSTELNKPAMIYCSHGDGH---WVHAQCMDLAERTLIHLSAGS--NKYYCNEHV
TAF3(867) ICPG-CNKPDDG-----------SPMIGCDD---CDD-WYHWPCVGIMT-------APPEEMQWFCPKCA
ING2(216) YCL--CNQVSYG------------EMIGCDNEQ-CPIEWFHFSCVSLTYKP---------KGKWYCPKCR
(C)
CFP1 CxxC domain
R200
G
C
DNA
(D)
R200
CFP1(163) KRSARMCGECEACRRTEDCGHCDFCRDMKKFGGPNKIRQKCRLRQCQLRARE
KDM2A(565) RRRRVRCRKCKACVQGE-CGVCHYCRDMKKFGGPGRMKQSCVLRQCLAPRLP
KDM2B(607) RRRRTRCRKCEACLRTE-CGECHFCKDMKKFGGPGRMKQSCIMRQCIAPVLP
FBXL19(33) RRRRTRCRRCRACVRTE-CGDCHFCRDMKKFGGPGRMKQSCLLRQCTAPVLP
DNMT1(663) AFKRRRCGVCEVCQQPE-CGKCKACKDMVKFGGSGRSKQACQERRCPNMAMK
MLL1(1149) GRRSRRCGQCPGCQVPEDCGVCTNCLDKPKFGGRNIKKQCCKMRKCQNLQWM
MLL2(960) KMRMARCGHCRGCLRVQDCGSCVNCLDKPKFGGPNTKKQCCVYRKCDKIEAR
(E)
K389 Y390
Hs CFP1(366) ERADAKDPASLPQCLGPGCVRPAQPSSKYCSDDCGMKLA
Mm CFP1(366) ERADAKDPASLPQCLGPGCVRAAQPSSKYCSDDCGMKLA
Xl CFP1(282) ERMETRDFSSLRQCLGPSCVQPARANSKYCSDDCGMKLA
Dr CFP1(269) DRTDGRHGGDTQQCLGPNCIEPARPNSKYCSEDCGMKLA
Dm CFP1(260) EIFLNPELQGIRQCYGPNCCSHARPQSKYCSDKCGFNLA
Sc Spp1(84) GEGSLPKTLWKRKCRISDCYKPCLQDSKYCSEEHGREFV

Figure S2

Figure S2- Rational design of CFP1 mutations. Related to Figure 1.

(A) To identify residues that would abrogate the association of the CFP1 PHD domain with methylated lysine ligands we built a structural model of the human CFP1 PHD aromatic cage based on the crystal structure of the BPTF PHD domain in complex with an H3K4me3 peptide (PDB ID - 2FUU). This model predicts that the peptide (cyan) would project its methyl-lysine residue into an aromatic cage comprised of residues Y28, M40 and W49 of CFP1. We chose to substitute W49 with alanine to create the PHD* mutant as this is predicted to inhibit methyl-lysine binding but not result in unfolding of the PHD domain (Li et al., 2006, Pena et al., 2006, Ramon-Maiques et al., 2007)
(B) Multiple sequence alignment of the PHD domains of human CFP1 (NP_001095124.1) and the known H3K4me3 binding PHD domains of MLL1 (NP_001184033.1), BPTF (NP_872579.2), RAG2 (NP_000527.2), TAF3 (NP_114129.1), and ING2 (NP_075992.2). The numbers in brackets next to each sequence indicate the starting residue number within the intact protein that corresponds to the first residue of the alignment. Asterisks mark zinc coordinating residues that are required for domain structure. The position of W49 in CFP1 is indicated by a blue arrow.
(C) The published crystal structure (PDB 3QMG) of the human CFP1 CXXC domain bound to DNA containing a non-methylated CpG dinucleotide (Xu et al., 2011) illustrates recognition of the C-G base pair by arginine 200 in CFP1 (R200). We chose to substitute R200 with alanine to create the CXXC* mutant as this substitution is predicted to inhibit binding to non-methylated CpG but not to result in an unfolded CXXC domain (Allen et al., 2006, Blackledge et al., 2010, Cierpicki et al., 2010, Zhou et al., 2012).
(D) Multiple sequence alignment of human nonmethyl-CpG-binding CXXC domains from CFP1 (NP_001095124.1), KDM2A (NP_036440.1), KDM2B (NP_115979.3), FBXL19 (NP_001093254.2), DNMT1 (NP_001124295.1), and MLL1 (NP_001184033.1), MLL2 (NP_055542.1). The numbers in brackets next to each sequence indicate the starting residue number within the intact protein that corresponds to the first residue of the alignment. Asterisks mark zinc coordinating residues that are required for domain structure, and the position of CFP1 R200 is indicated with a yellow arrow.
(E) Multiple sequence alignment of CFP1 SET1 interactions domains (SID) from *Homo sapiens* (NP_001095124.1), *Mus musculus* (NP_083144.1), *Xenopus laevis* (NP_001085408.1), *Danio rerio* (NP_956627.1), *Drosophila melanogaster* (NP_572556.1) and the *Saccharomyces cerevisiae* homologue Spp1 (ONH80964.1). The numbers in brackets next to each sequence indicate the starting residue number within the intact protein that corresponds to the first residue of the alignment. Cysteines in this domain (marked by asterisk) are predicted to create a structural zinc finger like fold to support interaction with SET1A. To create a SID mutant that would not affect the structure of CFP1 but should inhibit binding to SET1A we chose to substitute the highly conserved residues K389 and Y390 in CFP1 (Pink arrows) with alanine.

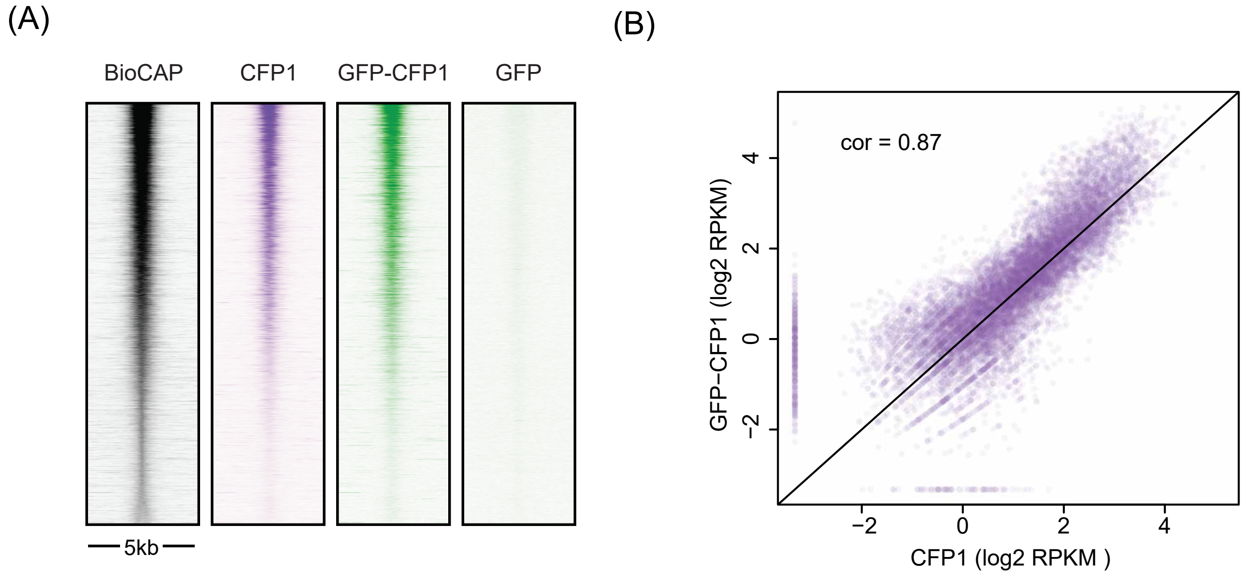
(A)
BioCAP
CFP1
GFP-CFP1
GFP
5kb
(B)
cor = 0.87
GFP−CFP1 (log2 RPKM)
CFP1 (log2 RPKM)
−2
0
2
4

Figure S3

Figure S3- GFP-CFP1 recapitulates endogenous CFP1 occupancy on chromatin. Related to Figure 2.

(A) A heatmap of CFP1, GFP-CFP1, and GFP ChIP-seq signal over all NMIs ranked by Bio-CAP intensity.
(B) A scatterplot of GFP-CFP1 and CFP1 ChIP-seq signal at NMIs. The ChIP-seq signal for GFP-CFP1 and endogenous CFP1 are highly correlated (R=0.87, Spearman Rank correlation) indicating that GFP-CFP1 recapitulates endogenous CFP1 occupancy on chromatin.

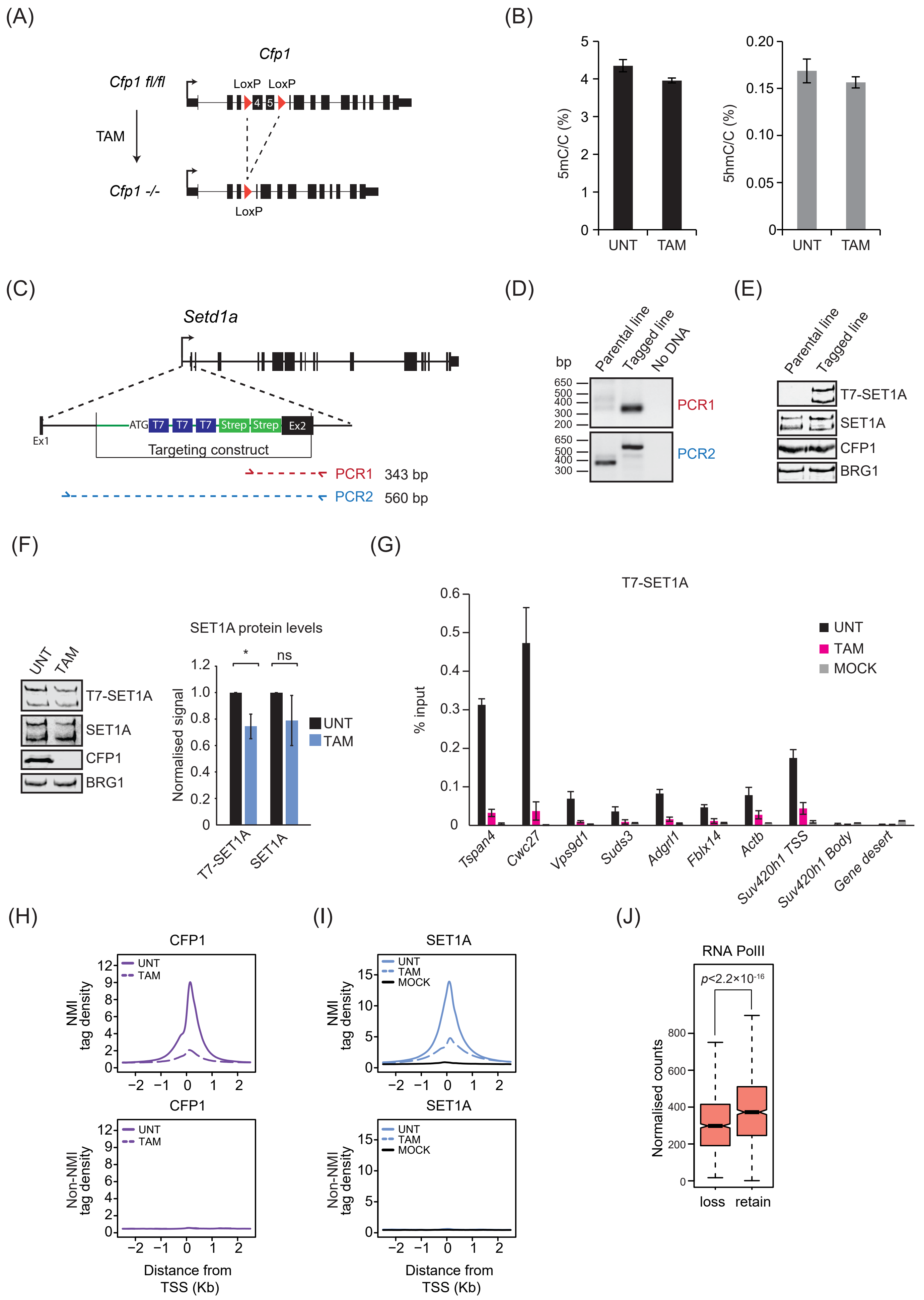
(A)
Cfp1
Cfp1 fl/fl
LoxP LoxP
TAM
Cfp1 -/-
LoxP
(B)
5mC/C (%)
5hmC/C (%)
UNT TAM
UNT TAM
(C)
Setd1a
Ex1
ATG T7 T7 T7 Strep Strep Ex2
Targeting construct
PCR1 343 bp
PCR2 560 bp
(D)
bp
Parental line
Tagged line
No DNA
PCR1
PCR2
(E)
Parental line
Tagged line
T7-SET1A
SET1A
CFP1
BRG1
(F)
UNT TAM
T7-SET1A
SET1A
CFP1
BRG1
SET1A protein levels
Normalised signal
UNT
TAM
T7-SET1A
SET1A
(G)
T7-SET1A
% input
UNT
TAM
MOCK
Tspan4
Cwc27
Vps9d1
Suds3
Adgrl1
Fblx14
Actb
Suv420h1 TSS
Suv420h1 Body
Gene desert
(H)
CFP1
NMI tag density
Non-NMI tag density
UNT
TAM
Distance from TSS (Kb)
(I)
SET1A
NMI tag density
Non-NMI tag density
UNT
TAM
MOCK
Distance from TSS (Kb)
(J)
RNA PolII
p<2.2×10-16
Normalised counts
loss
retain

Figure S4

Figure S4- CFP1 is the central determinant in SET1A occupancy on chromatin. Related to Figure 4.

(A) A schematic illustrating the location of the engineered loxP sites in the *Cfp1* gene.
(B) Graphs indicating the percentage of cystosine that is methylated (5mC) and hydroxymethylated (5hmC) in untreated (UNT) and tamoxifen treated (TAM) *Cfp1*$^{fl/fl}$ ESCs, as determined by HPLC. Error bars correspond to the SEM for 3 biological replicates. There are no significant alterations in 5mC or 5hmC following removal of CFP1.
(C) A schematic illustrating how the triple T7 double StrepII tag was knocked into the endogenous *Setd1a* gene in the *Cfp1*$^{fl/fl}$ ESCs. The PCR primers used to screen for homologous recombination are indicated.
(D) A genomic PCR verifying homozygous epitope tagging of the endogenous *Setd1a* gene.
(E) Western blot analysis of SET1A in the parental and epitope tagged cell line with epitope tag specific T7 antibody (upper panel) and SET1A specific antibodies (second panel from the top).
(F) Western blot analysis of SET1A in untreated (UNT) or tamoxifen treated (TAM) *Cfp1*$^{fl/fl}$ ESCs using epitope tag and SET1A-specific antibodies (left panel). The levels of SET1A under these conditions were analysed in at least biological triplicate and quantified (right panel) with error bars corresponding to the SD. This illustrates that loss of CFP1 leads to only a minor reduction in SET1A protein levels (* represents a student's t-test $p \leq 0.05$).
(G) ChIP-qPCR analysis of epitope tagged SET1A protein occupancy at a series of target gene promoters in untreated (UNT) and tamoxifen treated (TAM) *Cfp1*$^{fl/fl}$ ESCs with T7-tagged SET1A, and in the untagged *Cfp1*$^{fl/fl}$ cell line (MOCK). Error bars represent the SD from at least 3 biological replicates. This demonstrates the loss of SET1A binding following removal of CFP1 in agreement with ChIP-seq analysis.
(H) Metaplot analysis of CFP1 ChIP-seq at NMI (upper panel) and non-NMI sites (lower panel) in untreated (UNT, solid line) and tamoxifen treated (TAM, dotted line) *Cfp1*$^{fl/fl}$ ESCs.
(I) Metaplot analysis of SET1A ChIP-seq at NMI (upper panel) and non-NMI sites (lower panel) in untreated (UNT, solid line) and tamoxifen treated (TAM, dotted line) *Cfp1*$^{fl/fl}$ ESCs.
(J) Box plot illustrating RNA PolII ChIP-seq signal (8WG16, (Brookes et al., 2012)) at the top 10% of genes that lose or retain SET1A. This illustrates that genes that retain the most SET1A have higher levels of RNA PolII than those that lose the most SET1A. The *p* value denotes statistical significance calculated by a Wilcoxon signed rank test.

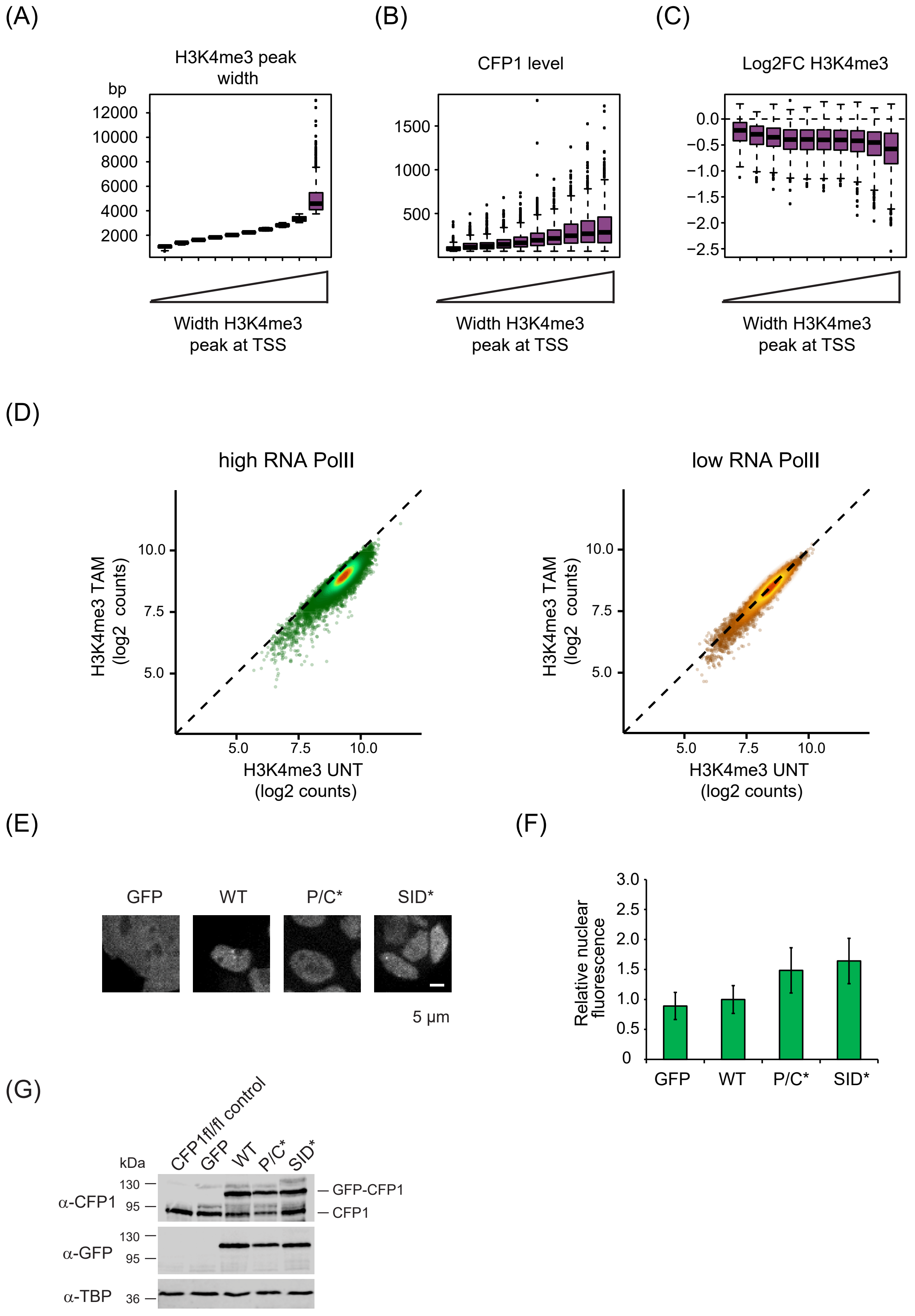
(A)
H3K4me3 peak width
bp
12000
10000
8000
6000
4000
2000
Width H3K4me3 peak at TSS
(B)
CFP1 level
1500
1000
500
Width H3K4me3 peak at TSS
(C)
Log2FC H3K4me3
0.0
−0.5
−1.0
−1.5
−2.0
−2.5
Width H3K4me3 peak at TSS
(D)
high RNA PolII
low RNA PolII
H3K4me3 TAM (log2 counts)
H3K4me3 UNT (log2 counts)
5.0
7.5
10.0
(E)
GFP
WT
P/C*
SID*
5 µm
(F)
Relative nuclear fluorescence
3.0
2.5
2.0
1.5
1.0
0.5
0
GFP
WT
P/C*
SID*
(G)
kDa
CFP1fl/fl control
GFP
WT
P/C*
SID*
α-CFP1
130
95
GFP-CFP1
CFP1
α-GFP
130
95
α-TBP
36

Figure S5

Figure S5- Broad peaks of H3K4me3 are most affected by loss of CFP1. Related to Figure 5.

(A-C) Non-divergent CFP1+ NMI associated genes were binned equally based on the width of their H3K4me3 peak as illustrated in (A). CFP1 levels and log2 fold change in H3K4me3 were then box plotted within the same bins (B and C). This revealed that the most prominent reduction in H3K4me3 following CFP1 loss occurred at genes with higher levels of CFP1 and that had wider peaks of H3K4me3.

(D) A scatterplot illustrating that genes with high RNA PolII occupancy (left panel) but not those with low RNA PolII occupancy (right panel) lose H3K4me3 at their TSS following tamoxifen treatment. The scatterplots correspond to non-divergent genes with an H3K4me3 peak overlapping their TSS and a gene was considered to have high RNA PolII if it had more than 2^6.2 PolII 8WG16 (Brookes et al., 2012) counts at the TSS.

(E) Live cell images of stable GFP and GFP fusion protein expression in mouse ESCs demonstrating nuclear localisation. The scale bar corresponds to 5 µm.

(F) Quantitation of GFP and GFP fusion protein fluorescence in stably expressing mouse ESCs, indicating equivalent protein expression. Error bars correspond to the SEM from 3 biological replicates.

(G) Western blot analysis using CFP1- and GFP-specific antibodies, indicating that GFP-CFP1 protein levels in *Cfp1*$^{fl/fl}$ ESC rescue lines (GFP-CFP1 WT, P/C* and SID*) are very similar endogenous CFP1 levels in both *Cfp1*$^{fl/fl}$ and GFP-only control cells. Western blot analysis with a TATA box binding protein (TBP)-specific antibody was used to demonstrate equal loading of nuclear extracts.

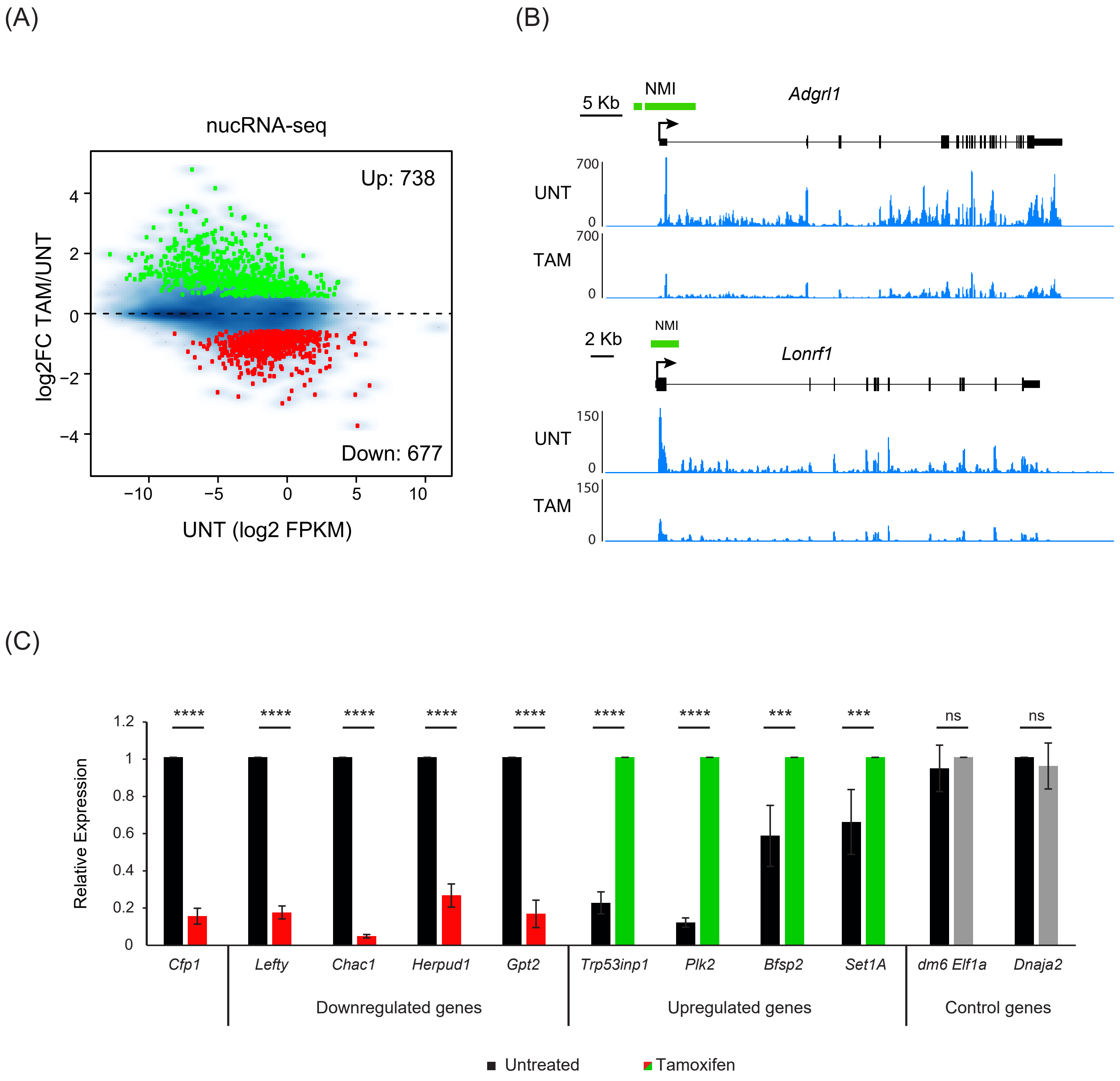

(A)
nucRNA-seq
Up: 738
Down: 677
log2FC TAM/UNT
UNT (log2 FPKM)
(B)
5 Kb
NMI
Adgrl1
UNT
TAM
700
0
2 Kb
Lonrf1
150
(C)
Relative Expression
****
***
ns
Cfp1
Lefty
Chac1
Herpud1
Gpt2
Trp53inp1
Plk2
Bfsp2
Set1A
dm6 Elf1a
Dnaja2
Downregulated genes
Upregulated genes
Control genes
Untreated
Tamoxifen

Figure S6

Figure S6- Loss of CFP1 leads to widespread effects on gene expression. Related to Figure 6.

(A) An MA plot showing log2 fold change in nuclear RNA-seq signal of non-CFP1 bound genes in untreated (UNT) and tamoxifen treated (TAM) *Cfp1*$^{fl/fl}$ ESCs. Red and green points depict significantly down- (677) and upregulated genes (738) that change in expression by more than 1.5-fold.
(B) Genomic snapshots showing examples of two CFP1-bound NMI genes that show reductions in gene expression following loss of CFP1.
(C) Quantitative RT-qPCR validating gene expression changes observed by nuclear-RNA-seq at series of misregulated genes (**** represents a student's t-test $p \leq 0.0001$ and *** $p \leq 0.001$). Grey bars correspond to control genes which do not change expression in mouse (*Dnaja*) or the *Drosophila* (*Elf1a*) calibration sample following tamoxifen treatment.

Supplementary Experimental Procedures:

Cell culture

Mouse C127 cells were grown at 37°C and 5% CO_2 in Dulbecco's Modified Eagle Medium (DMEM) supplemented with 10% fetal bovine serum (Biosera) and 1x penicillin-streptomycin solution (Gibco). Embryonic stem cells were grown on gelatin-coated dishes in DMEM supplemented with 15% fetal bovine serum (Biosera), 1x MEM non-essential amino acids (Gibco), 2 mM L-glutamine (Gibco), 1x penicillin-streptomycin solution (Gibco), 0.5 mM b-mercaptoethanol (Gibco), and leukemia inhibitory factor. *Drosophila* S2 (SG4) cells were grown adhesively at 25°C in Schneider Drosophila Medium (Gibco), supplemented with 1x penicillin-streptomycin solution and 10% fetal bovine serum (Biosera) that was previously heat-inactivated at 60°C for 30 min.

Stable transgene expression

cDNAs were inserted into a modified pCAG-IRES-puro expression vector containing an N-terminal GFP tag by ligation independent cloning. To generate C127 cell lines expressing GFP-fusions, expression vectors were transfected using Fugene HD (Promega). Following transfection, cells were selected with 2.5 µg/ml puromycin until individual colonies formed and clonal isolates for individual GFP-fusions were selected that displayed equal transgene expression. To generate cell lines expressing GFP-CFP1 in CFP1 conditional mouse embryonic stem cells, *Cfp1$^{fl/fl}$:Rosa26-ERT2CRE* cells were transfected with the appropriate expression vector using Lipofectamine 2000 (Invitrogen) and selected with 1 µg/ml puromycin until individual colonies formed. Clonal isolates for individual GFP-fusions were selected that displayed equal transgene expression.

CRISPR/Cas9-mediated knock-in

To insert the 3xT7-2xStrepII tag into the *Setd1a* gene we employed CRISPR/Cas9 aided gene targeting as reported in (Ran et al., 2013). A guide RNA (gRNA) was identified (http://crispr.mit.edu/) which overlapped with the region proximal to the ATG of the *Setd1a* gene. A gene targeting construct was generated by PCR amplification that had the 3xT7-2xStrepII tag flanked by roughly 160 bp homology arms to act as repair template for SET1A tagging (Figure S4). *Cfp1$^{fl/fl}$* ESCs were transiently co-transfected with the Cas9 plasmid containing the sgRNA sequence and with the targeting construct (Lipofectamine 3000, Invitrogen). After 24h, 0.5 µg/mL puromycin was added for 48 h to enrich for transfected cells. Puromycin selected cells were and then plated at limiting dilution without puromycin and individual clones were allowed to form. Individual clones were then screened by western blot and homozygote tagging validated by PCR on genomic DNA.

Fluorescence recovery after photobleaching (FRAP)

35 mm µdishes (Ibidi) were seeded with 60,000 C127 cells and imaged after 24 h. Imaging was performed in phenol red-free DMEM (Lonza) containing 10% FCS, 12.5 mM HEPES (Gibco) and 40 µM sodium pyruvate (Gibco), and maintained at 37°C and 5% CO_2 in a humidified incubation chamber (Tokai Hit). FRAP experiments were performed on an UltraView spinning disk microscope (Perkin Elmer) equipped with and EM-CCD camera (Hamamatsu) using a 60x/1.4NA oil objective. 50 pre-

bleach and 1000 post-bleach images were captured at a rate of 8 fps (Figure 1B and C) after bleaching a circular diffraction limited spot of ~2.5 µm diameter using 488nm laser line at 100% transmission. Alternatively, to capture the rapid recover of the P/C/S* mutant effectively we used an acquisition rate of 13 fps in Figure 1E and F. FRAP curves were calculated in MATLAB, normalizing for the initial conditions (brightness of the cell and brightness of the spot) and corrected for acquisition photobleaching over time (Mueller et al., 2012). Half recovery times ($t_{1/2}$) were calculated using a biexponential fit. Briefly, this involved deriving $t_{1/2}$ values from individual cells (Figure S1F and G) and then collecting the distribution of $t_{1/2}$ values across biological triplicates for the same transgene (Figure S1H and I). To compare the dynamics of individual GFP-CFP1 transgenes, a student's t-test was then used to calculate the probability (p) that there was no difference between the wild-type and mutant versions of CFP1.

Antibodies

A CFP1 antibody was generated by immunizing a rabbit (PTU/BS Scottish National Blood Transfusion Service) with a 6xHis tagged protein antigen encoding amino acids 206 to 360 of human CFP1. α-CFP1 was then affinity purified against the same antigen immobilised on an Affigel10 resin as described previously (Farcas et al., 2012). The source and use of other antibodies is indicated in the table below.

Antibody	Type	Source	USE
α-CFP1f	Rabbit polyclonal	This Study	ChIP and Western
α-KDM2B	Rabbit polyclonal	(Farcas et al., 2012)	ChIP and Western
α-GFP	Mouse monoclonal	Invitrogen (3E6)	ChIP
α-Pol II CTD	Mouse monoclonal	Covance (8WG16)	ChIP and Western
α-T7-Tag XP®	Rabbit monoclonal	Cell Signalling (D9E1X)	ChIP and Western
α-SET1A	Rabbit polyclonal	Bethyl (A300-289A)	Western
α-BRG1	Rabbit monoclonal	Abcam (ab110641)	Western
α-H3K4me3	Rabbit polyclonal	(Farcas et al., 2012)	ChIP and Western
α-H3K4me2	Rabbit monoclonal	Abcam (Y47 - ab32365)	Western
α-H3K4me1	Rabbit polyclonal	Abcam (ab8895)	Western
α-H2A	Mouse monclonal	Cell Signalling (L88A6)	Western

Recombinant protein expression

The CFP1 PHD finger construct (residues 23 – 105) was cloned into the pGEX-6P-1 (GE Healthcare) expression vector with ampicillin resistance. Protein was expressed in *E. coli* BL21 (DE3) RIL cells grown in either Luria Broth or $^{15}NH_4Cl$ minimal media, supplemented with 60 µM $ZnCl_2$. After induction with IPTG (0.5 mM) for 16 h at 18°C, cells were harvested and lysed by sonication. GST-fusion proteins were purified on glutathione Sepharose 4B beads (GE Healthcare). The GST tag was cleaved with PreScission protease (Amersham). When necessary, the proteins were further purified by size exclusion chromatography over a HiPrep 16/60 Sephacryl S-100 column (GE Healthcare) and concentrated in Millipore concentrators (Millipore).

Protein extraction and western blot

Cfp1$^{fl/fl}$ ESCs were harvested and washed in PBS. For histone extraction, pellets were resuspended in TEB buffer (0.5% Triton X-100 in PBS, supplemented with 1x Complete EDTA-free inhibitor cocktail, 1 mM 4-(2-Aminoethyl)benzenesulfonyl fluoride hydrochloride [AEBSF]) and rotated for 10 min at 4°C. Nuclei were then collected by centrifugation for 10 min at 6500 g and histones were extracted with 0.2 N HCl during overnight rotation at 4°C. Protein concentration was measured by Bradford assay and equal amounts of histone were separated on an 18.7% SDS-polyacrylamide gel and subjected to western blot analysis.

For nuclear extract, cells were resuspended in 10 volumes of Buffer A (10 mM HEPES pH 7.9, 1.5 mM $MgCl_2$, 10 mM KCl, 0.5 mM-DTT, 1x Complete EDTA-free inhibitor cocktail, 1 mM AEBSF) and incubated 10 min on ice. Recovered pellets were then resuspended in 3 volumes of Buffer A supplemented with 0.1% NP40 and inverted 10 times. Resulting nuclei were recovered by centrifugation and were resuspended in Buffer C (5 mM HEPES pH 7.9, 26% glycerol, 1.5 mM $MgCl_2$, 0.2 mM EDTA, 0.5 mM DTT, 400 mM NaCl, supplemented with 1x Complete EDTA-free inhibitor cocktail, 1 mM AEBSF) and incubated for 1 h on ice, followed by centrifugation and recovery of the soluble fraction. Protein concentration was measured by Bradford assay and equal amounts of protein were used for SDS-PAGE and western blotting. To quantify western blot signals secondary antibodies conjugated with infrared dyes (IRDye 800CW goat anti-rabbit or IRDye 680RD goat anti-mouse, LI-COR) and a LI-COR FC instrument were used.

NMR titrations of histone peptides

The ^{1}H,^{15}N HSQC spectra of 0.1 – 0.2 mM uniformly ^{15}N-labeled CFP1 PHD finger in 20 mM Tris-HCl buffer pH 6.8, 100 mM NaCl, 2.5 mM DTT, and 7% D_2O were collected on a Varian INOVA 600 MHz spectrometer. The spectra were recorded at 298K using 1024 × 128 increments, and a spectral width of 8820 × 1974 Hz in the ^{1}H and ^{15}N dimensions, respectively. The binding was characterized by monitoring chemical shift changes as histone tail peptides (synthesized by the University of Colorado Denver Peptide Core Facility) were added stepwise. The dissociation constants (K_ds) were determined using a nonlinear least-squares analysis in KaleidaGraph and the equation:

$$\Delta\delta = \Delta\delta_{\max}\left(\left([L]+[P]+K_d\right)-\sqrt{\left([L]+[P]+K_d\right)^2-4[P][L]}\right)\Big/2[P]$$

where [L] is concentration of the peptide, [P] is concentration of the protein, Δδ is the observed chemical shift change, and $\Delta\delta_{max}$ is the normalized chemical shift change at saturation. Normalized chemical shift changes were calculated using the equation

$$\Delta\delta=\sqrt{\left(\Delta\delta H\right)^2+\left(\Delta\delta N/5\right)^2}$$

where Δδ is the change in chemical shift in parts per million (ppm).

Fluorescence spectroscopy

Spectra were recorded at 25˚C on a Fluoromax-3 spectrofluorometer (HORIBA). The samples containing the CFP1 PHD finger in 20 mM Tris-HCl buffer pH 6.8, 100 mM NaCl, 2.5 mM DTT and progressively increasing concentrations of the histone peptide were excited at 280 nm. Emission spectra were recorded over a range of wavelengths between 320 and 380 nm with a 1 nm step size

and a 1 s integration time and averaged over 3 scans. The K_d values were determined using a nonlinear least-squares analysis and the equation:

$$\Delta I = \Delta I_{\max}\left(\left([L]+[P]+K_d\right)-\sqrt{\left([L]+[P]+K_d\right)^2-4[P][L]}\right)\Big/2[P]$$

where [L] is the concentration of the histone peptide, [P] is the concentration of CFP1 PHD finger, ΔI is the observed change of signal intensity, and ΔI_{max} is the difference in signal intensity of the free and bound states of the PHD finger. The K_d value was averaged over three separate experiments, with error calculated as the standard deviation between the runs.

HPLC

Genomic DNA was treated with 1U RNase A (Thermo Scientific) per 10 µg, purified by phenol chloroform ethanol precipitation and incubated overnight in hydrolysis solution (45 mM NaCl, 9 mM $MgCl_2$, 9 mM Tris-HCl pH 7.9, ≥ 250 U/ml Benzonase (Sigma), 50 mU/ml Phosphodiesterase I, ≥ 20 U/ml Alkaline phosphatase, 46.8 ng/ml EHNA hydrochloride, 8.64 µM deferoxamine). Protein components were removed by centrifugation through Amicon centrifugal filter unit (3 kDa cut-off, Millipore) before samples were lyophilised and resuspended in buffer A. Nucleosides were resolved with an Agilent UHPLC 1290 instrument fitted with Eclipse Plus C18 RRHD 1.8 µm (2.1 × 150 mm column) and detected and quantified with Agilent 1290 DAD fitted with a Max-Light 60 mm cell. Buffer A was 100 mM ammonium acetate pH 6.5, buffer B was 40% acetonitrile and the flow rate 0.4 ml min^{-1}. The gradient was between 1.8–100% of 40% acetonitrile with the following steps: 1–2 min, 100% A; 2–16 min 98.2% A, 1.8% B; 16–18 min 70% A, 30% B; 18–20 min 50% A, 50% B; 20–21.5 min 25% A, 75% B; 21.5–22.5 min 100% B; 22.5-24.5 min 100% A. Relative abundance of 5mC and 5hmC were established by detection of adenosine at 280nm allowing determination of total cytosine by extinction coefficient calculation using standards.

Bio-CAP sequencing

Bio-CAP sequencing was performed on genomic DNA isolated from C127 cells as described in (Blackledge et al., 2012, Long et al., 2013).

ATAC-seq

Chromatin accessibility was assayed using an adaptation of the assay for transposase accessible-chromatin (ATAC)-seq (Buenrostro et al., 2013). Briefly, 5×10^6 cells were harvested, washed with PBS and nuclei were isolated using 1 mL HS Lysis buffer (50 mM KCl, 10 mM $MgSO_4 \cdot 7H_20$, 5 mM HEPES, 0.05 % NP40, 1 mM PMSF, 3 mM DTT) for 1 min at room temperature. Nuclei were centrifuged at 1000 g for 5 min at 4°C, followed by a total of three washes with ice-cold RSB buffer (10 mM NaCl, 10 mM Tris-HCl pH 7.4, 3 mM $MgCl_2$), to remove as much of contaminating cytoplasmic and mitochondrial material as possible. Nuclei were then counted, and 5×10^4 nuclei were resuspended in Tn5 reaction buffer (10 mM TAPS, 5 mM $MgCl_2$, 10% dimethylformamide) and 2 µl of Tn5 transposase (25 µM) made in house as previously described (Picelli et al., 2014). Nuclei were then incubated for 30 min at 37°C, before isolation and purification of tagmented DNA using QiaQuick MinElute columns (Qiagen). To control for sequence bias of the Tn5 transposase, an ATAC "input" sample was generated, by tagmenting genomic DNA from ESCs with Tn5 for 30 min at 55°C. ATAC-seq libraries were prepared by PCR amplification using custom made Illumina barcodes previously described (Buenrostro et al., 2013)

and the NEBNext® High-Fidelity 2X PCR Master Mix with 8-10 cycles. Libraries were purified with two rounds of AMPure XP bead cleanup (1.5X beads:sample), followed by quantification by qPCR using SensiMix SYBR (Bioline) and KAPA Library Quantification DNA standards (KAPA Biosystems). ATAC-seq libraries were sequenced on Illumina NextSeq500 using 80 bp paired-end reads in biological triplicate.

ChIP and ChIP-sequencing

For C127 cells, H3K4me3 ChIP was performed by crosslinking cells in PBS with 1% formaldehyde for 15 min at 25˚C and was quenched with 150 mM glycine. For CFP1, KDM2B and RNAPII ChIP-seq, cells were fixed with 2 µM EGS for 1 h prior to the formaldehyde crosslinking. Crosslinked cells were incubated in lysis buffer (50 mM HEPES pH 7.9, 140 mM NaCl, 1 mM EDTA, 10% glycerol, 0.5% NP40, 0.25% TritonX-100) for 10 min at 4˚C. The released nuclei were then washed (10 mM Tris-HCl, pH 8.0, 200 mM NaCl, 1 mM EDTA, 0.5 mM EGTA) for 10 min at 4˚C. Chromatin was resuspended (10 mM Tris-HCl pH 8.0, 100 mM NaCl, 1 mM EDTA, 0.5 mM EGTA, 0.1% Na deoxycholate, 0.5% N-lauroylsarcosine) and sonicated for 50 min using a BioRuptor sonicator (Diagenode), shearing genomic DNA into 0.5–1 kb fragments. After sonication, TritonX-100 was added to a final concentration of 1.5%. Following centrifugation at 19000 g for 10 min at 4˚C, the supernatant containing soluble chromatin was isolated from the insoluble fraction. The relevant antibodies were incubated with 750 µg of Protein A Dynabeads (Novex) in PBS/BSA 0.5% rotating at 4˚C for 4 h. The beads were then washed three times in PBS/BSA 0.5%, to remove unbound antibody. Prior to immunoprecipitation, chromatin was diluted (20 mM Tris-HCl pH 8.0, 150 mM NaCl, 1 mM EDTA, 1% TritonX-100) 10-fold (or 50-fold for H3K4me3). Immunoprecipitations were performed using 1 ml of diluted chromatin per 25 µl of antibody coated beads, and rotated overnight at 4˚C. This is equivalent to $5x10^6$ cells (or $1x10^6$ for H3K4me3 ChIP). The IP was then washed with low salt buffer (0.1% SDS, 1% Triton X-100, 2 mM EDTA, 20 mM Tris-HCl pH8.0, 150 mM NaCl), then with high salt buffer (0.1% SDS, 1% Triton X-100, 2 mM EDTA, 20 mM Tris-HCl pH8.0, 500 mM NaCl), followed by LiCl buffer (0.25 M LiCl, 1% NP-40, 1% SDS, 1 mM EDTA, 10 mM Tris-HCl pH 8.0) and twice with TE (20 mM Tris-HCl pH8.0, 1 mM EDTA pH 8.0). Chromatin was eluted in 100 µl of elution buffer (100 mM $NaCO_3$, 0.1% SDS) by vigorous shaking for 30 min at 30°C. Crosslinks were reversed by the addition of 4 µl of 5 M NaCl and 2 µl of 500 µg/ml RNaseA (Roche) at 65˚C followed by incubation at 42˚C for 1.5 h with 1 µl of 20 mg/ml Proteinase K to remove protein. ChIP DNA was purified ChIP DNA Clean & Concentrator kit (Zymo Research).

Cfp1$^{fl/fl}$ ESCs were cultured for 96 h in presence or absence of 4-Hydroxytamoxifen (4-OHT). ChIP-seq was carried out in at least biological duplicate for each condition. To carry out ChIP an equal number of untreated and treated cells were resuspended in 10 mL of PBS and subjected to crosslink with 1% methanol-free formaldehyde (ThermoFisher) for 10 min at 25°C and quenched with 150 mM glycine for 10 min at RT. For T7-SET1A ChIP, cells were crosslinked in 2 µM disuccinimidyl glutarate (DSG, ThermoFisher) at 25° C for 50 mins and then 1% formaldehyde for 10 min at 25°C. Cells were lysed on ice for 10 min in 1 mL of lysis buffer (50 mM HEPES pH7.9, 300 mM NaCl, 1 mM EDTA, 0.5 mM EGTA, 0.5% NP-40, 0.1% Na deoxycholate, 0.1% SDS, 1x Complete EDTA-free inhibitor cocktail, 1 mM AEBSF) and then sonicated with Bioruptor Pico (Diagenode) for 20 min with 30s ON/OFF cycles (for double crosslinked chromatin, cells were sonicated for 23 min). Lysates were centrifuged for 10 min at 16000 g and the cleared chromatin was recovered and DNA quantified. For each IP, 300 µg of chromatin was diluted to 1 mL of lysis buffer and pre-cleared for 1 h at 4°C with IPA 300 resin beads (Repligen) blocked with yeast tRNA and BSA. Antibody was then added to each IP and incubated overnight, followed by

isolation of antibody chromatin complexes with 20 µL of blocked IPA300 beads per IP. Immunoprecipitates were washed 1x with lysis buffer, 1x with lysis buffer with 500 mM NaCl, 1x DOC buffer (10 mM Tris-HCl pH 8.0, 250 mM LiCl, 1 mM EDTA, 0.5% NP40, 0.5% Na- deoxycholate), and 2x with TE pH 8. Chromatin was then eluted in 200 µL of elution buffer (1% SDS, 0.1 M $NaHCO_3$) for 30 min at 30°C with vigorous shaking. Eluates and inputs were treated with DNase-free RNase (ThermoFisher) and crosslinks were reversed at 65˚C overnight with 200 mM NaCl and ProteinaseK solution (Sigma). DNA was purified with the ChIP DNA Clean & Concentrator kit (Zymo Research).
For massively parallel sequencing, DNAs were post-sonicated with Bioruptor Pico (Diagenode) to a DNA fragment size of 200-300 bp as determined by Bioanalyser analysis. Libraries were prepared with NEBNext Ultra DNA Library Prep Kit for Illumina and quantified by qPCR using KAPA Illumina DNA standards as reference. Libraries were sequenced either on an Illumina HiSeq2500 or NextSeq500.

Calibrated native ChIP-sequencing

ChIP sequencing for H3K4me3 in mouse ES cells was performed by calibrated native ChIP sequencing. Calibrated ChIP-seq was carried out in biological triplicate for each cell line and condition. This was achieved by adding $2.5x10^6$ *Drosophila* S2 (SG4) cells to $10x10^6$ untreated or tamoxifen treated *Cfp1*$^{fl/fl}$ ESCs. Nuclei were released by resuspending the mixed cell mixture in ice cold lysis buffer (10mM Tris-HCl pH 8.0, 10 mM NaCl, 3 mM $MgCl_2$, 0.1% NP40). Nuclei were then washed, and resuspended in 1 ml (10 mM Tris-HCl pH 8.0, 10 mM NaCl, 3 mM $MgCl_2$, 0.1% NP40, 0.25M sucrose, 3mM $CaCl_2$, 1x protease inhibitors (Sigma)), and incubated with 100U of MNase (Fermentas) at 37°C for 5 min followed by the addition of EDTA to halt the digestion. The supernatant was collected following centrifugation at 1500 g for 5 min at 4°C. The remaining pellet was incubated with 300 µl of nucleosome release buffer (10 mM Tris-HCl pH 7.5, 10 mM NaCl, 0.2 mM EDTA, 1x PIC) at 4°C for 1 h, then passed through a 27G needle using a 1 ml syringe, and spun at 1500 g for 5 min at 4°C. The two supernatants were combined and diluted 10-fold in native ChIP incubation buffer (70 mM NaCl, 10 mM Tris-HCl pH 7.5, 2 mM $MgCl_2$, 2 mM EDTA, 0.1% TritonX-100, 1x PIC). The relevant antibody was added to the diluted chromatin and rotated overnight at 4˚C. For each immunoprecipitation 20 µl of IPA300 agarose beads (RepliGen) were blocked with 1 mg/ml BSA and 1 mg/ml yeast tRNA in native ChIP incubation buffer (70 mM NaCl, 10 mM Tris-HCl pH 7.5, 2 mM $MgCl_2$, 2mM EDTA, 0.1% Triton X-100). Antibody/protein complexes were recovered by incubation with 20 µl of blocked beads per ChIP reaction, for 1 hour at 4°C, followed by centrifugation at 1000 g for 1 min. Beads were washed four times with native ChIP wash buffer (20 mM Tris-HCl pH 7.5, 2 mM EDTA, 125 mM NaCl, 0.1% Triton-X100), and once with 1 ml ice cold TE buffer. DNA was purified with the ChIP DNA Clean & Concentrator kit (Zymo Research). Libraries were prepared with NEBNext Ultra DNA Library Prep Kit for Illumina and quantified by qPCR using KAPA Illumina DNA standards as reference. Libraries were sequenced on an Illumina NextSeq500.

4sU RNA Sequencing

Nascent RNA sequencing was performed by pulse labelling with 4-thiouridine (4sU) as previously described (Radle et al., 2013). Confluent 15 cm plates were treated with 500 µM 4sU in 10 ml culture medium for 20 min. The 4sU-containing media was then aspirated and immediately replaced with 5 ml TRIZOL (Thermo Fisher). RNA was isolated by phenol-chloroform extraction using phase lock tubes (Eppendorf) and resuspend in nuclease-free water. Total RNA aliquots were treated with 6U Turbo

DNase (Ambion) and incubated at 37°C for 30 min, followed by inactivation of the DNase according to the manufacturer's instructions. 300 µg of the resulting total RNA were incubated with 2 µg Biotin-HPDP/µg RNA in biotinylation buffer (10 mM Tris-HCl pH 7.4 and 1 mM EDTA) and rotated for 1.5 h at room temperature. Unincorporated biotin-HPDP was removed by chloroform/isoamylalcohol extraction. To capture biotinylated RNA, µMACS streptavidin beads (Miltenyi) were added to the RNA suspension (1 µl of beads/ µg RNA) and rotated for 15 min at room temperature prior to loading onto µMACS minicolumns. Unlabelled RNA was removed by washing three times with 900 µl of wash buffer (100 mM Tris-HCl pH 7.5, 10 mM EDTA, 1 M NaCl, 0.1% Tween20) pre-heated at 65°C, then three more times with wash buffer at room temperature. Biotinylated RNA was then eluted in two rounds with 100 mM DTT and purified using an RNeasy miniElute kit (Qiagen). The RNA concentration was measured and at most 900 µg of biotinylated RNA was depleted of ribosomal RNAs using the Low Input RiboMinus Eukaryote System v2 kit (Thermo Fisher). The recovered rRNA-depleted biotinylated RNA was used to prepare the cDNA libraries with NEBNext Ultra Directional RNA Library Prep Kit for Illumina and subject to sequencing on Illumina NextSeq500 platform.

Quantitative nuclear RNA-seq

Cfp1$^{fl/fl}$ ESCs were cultured for 96 h in presence or absence of 4-OHT and the nuclear RNA-seq was carried out in biological quadruplicate for each condition. Cells were collected counted and $1x10^6$ *Drosphila* S2 (SG4) cells were added to $4x10^6$ cells from each condition. The cell mixture was recovered by centrifugation and resuspended at room temperature in lysis buffer (50 mM KCl, 10 mM $MgSO_4.7H_20$, 5 mM HEPES, 0.05% NP40) for 30 s and then incubated at room temperature for other 30 s and then chilled on ice. Nuclei were pelleted and gently washed in cold RBS buffer (10 mM NaCl, 10 mM Tris-HCl pH 7.4, 3 mM $MgCl_2$) three times. At the last wash step, an aliquot of nuclei corresponding to $4x10^5$ cells was collected for genomic DNA extraction, whilst the remaining nuclei were pelleted and resuspended in TRIZOL reagent (Life Technologies) and subject to conventional RNA extraction. Nuclear RNA was then treated with DNase (Ambion) for 1 h at 37°C to remove any genomic DNA contamination. The RNA concentration was measured and 900 µg of nuclear RNA was depleted of ribosomal RNAs using the NEBNext rRNA Depletion kit. The recovered rRNA-depleted RNA was used to prepare the cDNA libraries with NEBNext Ultra Directional RNA Library Prep Kit for Illumina. 10 ng of sonicated gDNA was used to prepare "input" DNA libraries using the NEBNext Ultra DNA Library Prep Kit for Illumina. nucRNA and gDNA libraries were quantified by qPCR using KAPA Illumina DNA standards as reference and pooled at equimolar ratios (gDNA libraries pool was used at $1/10^{th}$ of nucRNA libraries pool) and sequenced on the Illumina NextSeq500 platform.

ChIP-seq datasets processing

Reads were aligned to the mouse mm10 genome using bowtie2 (Langmead and Salzberg, 2012) with the '-no-mixed' and '-no-discordant' options, and non-uniquely mapping reads were discarded. Calibrated ChIP-Seq datasets were aligned to a concatenated genome (mm10+dm6), and reads which mapped more than once were discarded. PCR duplicates were removed using SAMtools (Li et al., 2009).

Calibrated ChIP-Seq normalization

To calibrate H3K4me3 ChIP-seq sequencing, the number of mouse reads was randomly down sampled using the number of drosophila reads in each sample as a normalization point. Genomic DNA sequencing of the input mixture of mouse and drosophila was also used to account for any variance in cell mixture ratios. Calibrated native H3K4me3 ChIP-seq tracks were generated using DANPOS2 (Chen et al., 2013).

Peak calling

C127 NMIs and peaks of CFP1 or H3K4me3 enrichment were identified using the MACS algorithm (Zhang et al., 2008), with an effective genome size of $1.87x10^9$, and a false discovery rate "-q" of 0.01. For duplicate and triplicate data, peaks were called for each replicate against a matched control, and were required to overlap in all replicates. H3K4me3 peaks within 250 bp of one another were merged and considered as one peak.

ChIP-Seq quantification

We used RefSeq transcripts (genome.ucsc.edu, downloaded on 21/08/2015) considering only genes mapping to a unique location in the genome. In cases where multiple TSSs located within 750 bp of each other, one transcript was randomly selected to be included in our gene set. Generally, intervals of TSS+/-1kb were used for all quantifications. For the H3K4me3 datasets, reads were quantified within peaks overlapping a given TSS, due to the high variability of peak widths. Furthermore, for these datasets the TSSs with a RefSeq divergent transcript within 2 kb were excluded, to preserve the information on the asymmetry of the H3K4me3 peak. ChIP-Seq replicates were randomly downsampled using SAMtools according to their library size or for calibrated ChIP-Seq based on the spike-in read ratio (see Calibrated ChIP-Seq normalization). For paired-end sequencing, normalized fragment coverage was quantified using the summarizeOverlaps() function from GenomicFeatures (Lawrence et al., 2013) in the mode Union and considering only primary alignments (ScanBamFlag() option isNotPrimaryRead=FALSE). Replicate counts were then pooled and 8 pseudocounts were added prior to log transformation. FPKM were calculated based on an average library size, due to the nature of already normalized counts.

Nuclear RNA-seq processing

Nuclear RNA-seq (nucRNA-Seq) and input genomic DNA reads were initially aligned against concatenated (mm10+dm6) rRNA genomic sequence (GenBank: BK000964.3 and M21017.1) using bowtie2 to filter out rRNA fragments, prior to alignment against the mm10 and dm6 genomes using the STAR RNA-seq aligner (Dobin et al., 2013). To improve mapping reads which failed to map using STAR were aligned against the genome using bowtie2 and reads which mapped more than once were discarded. PCR duplicates were removed using SAMtools (Li et al., 2009) and reads from mouse or drosophila genome were segregated into different bam files.

Nuclear RNA-Seq quantification and differential expression analysis

We used RefSeq transcripts (genome.ucsc.edu, downloaded on 21/08/2015) considering only genes mapping to a unique location in the genome. In cases where multiple TSSs located within 750 bp of each other, one transcript was randomly selected to be included in our gene set. In order to normalize gene counts to the calibration control, dm6-mapped nucRNA bam files were first downsampled using SAMtools based on the mm10/dm6 ratio in the input genomic DNA to control for any cell count variability between individual experiments. Fragment coverage at non-overlapping gene bodies of dm6 RefSeq transcripts (genome.ucsc.edu, downloaded on 14/09/2016) was quantified using the summarizeOverlaps() function in GenomicFeatures (Lawrence et al., 2013) in the mode Union and considering only primary alignments (ScanBamFlag() option is NotPrimaryRead=FALSE). Further normalization and differential expression analysis were performed in R (v 3.3.0) using the DESeq2 package (Love et al., 2014). Briefly, dm6 counts were used to quantify sizeFactors for subsequent differential expression analysis of raw fragment counts at mm10 RefSeq transcripts. Normalized counts were extracted from the DESeq2 object for each sample and each gene and used in subsequent analysis to calculate FPKM values. FPKM were calculated based on an average library size, due to the nature of already normalized counts. Log2FoldChange counts were extracted from the DESeq2 results table. Significantly differentially expressed genes were defined as having and adjusted p-value less than or equal to 0.01 and a fold change in expression of at least 1.5 fold.

Definition of gene categories

For ESC, all TSSs containing one NMI peak (Long et al., 2013) within 250 bp were defined as NMI TSSs. CFP1 targets were defined based on the bimodal distribution of log2 transformed CFP1 fragment counts around the TSS with a cutoff of > 6.1. To control for background, a further log2 fold change cutoff between untreated and 4-OHT-treated cells was introduced as log2FC=0.9. Highly transcribed genes were defined according to the bimodal distribution of FPKM values as genes with a log2(FPKM) higher than -2.5.

Supplementary References

ALLEN, M. D., GRUMMITT, C. G., HILCENKO, C., MIN, S. Y., TONKIN, L. M., JOHNSON, C. M., FREUND, S. M., BYCROFT, M. & WARREN, A. J. 2006. Solution structure of the nonmethyl-CpG-binding CXXC domain of the leukaemia-associated MLL histone methyltransferase. *EMBO J,* 25**,** 4503-12.

BLACKLEDGE, N. P., LONG, H. K., ZHOU, J. C., KRIAUCIONIS, S., PATIENT, R. & KLOSE, R. J. 2012. Bio-CAP: a versatile and highly sensitive technique to purify and characterise regions of non-methylated DNA. *Nucleic Acids Res,* 40**,** e32.

BLACKLEDGE, N. P., ZHOU, J. C., TOLSTORUKOV, M. Y., FARCAS, A. M., PARK, P. J. & KLOSE, R. J. 2010. CpG islands recruit a histone H3 lysine 36 demethylase. *Mol Cell,* 38**,** 179-90.

BROOKES, E., DE SANTIAGO, I., HEBENSTREIT, D., MORRIS, K. J., CARROLL, T., XIE, S. Q., STOCK, J. K., HEIDEMANN, M., EICK, D., NOZAKI, N., et al. 2012. Polycomb associates genome-wide with a specific RNA polymerase II variant, and regulates metabolic genes in ESCs. *Cell Stem Cell,* 10**,** 157-70.

BUENROSTRO, J. D., GIRESI, P. G., ZABA, L. C., CHANG, H. Y. & GREENLEAF, W. J. 2013. Transposition of native chromatin for fast and sensitive epigenomic profiling of open chromatin, DNA-binding proteins and nucleosome position. *Nat Methods,* 10**,** 1213-8.

CHEN, K., XI, Y., PAN, X., LI, Z., KAESTNER, K., TYLER, J., DENT, S., HE, X. & LI, W. 2013. DANPOS: dynamic analysis of nucleosome position and occupancy by sequencing. *Genome Res,* 23**,** 341-51.

CIERPICKI, T., RISNER, L. E., GREMBECKA, J., LUKASIK, S. M., POPOVIC, R., OMONKOWSKA, M., SHULTIS, D. D., ZELEZNIK-LE, N. J. & BUSHWELLER, J. H. 2010. Structure of the MLL CXXC domain-DNA complex and its functional role in MLL-AF9 leukemia. *Nat Struct Mol Biol,* 17**,** 62-8.

DOBIN, A., DAVIS, C. A., SCHLESINGER, F., DRENKOW, J., ZALESKI, C., JHA, S., BATUT, P., CHAISSON, M. & GINGERAS, T. R. 2013. STAR: ultrafast universal RNA-seq aligner. *Bioinformatics,* 29**,** 15-21.

FARCAS, A. M., BLACKLEDGE, N. P., SUDBERY, I., LONG, H. K., MCGOURAN, J. F., ROSE, N. R., LEE, S., SIMS, D., CERASE, A., SHEAHAN, T. W., et al. 2012. KDM2B links the Polycomb Repressive Complex 1 (PRC1) to recognition of CpG islands. *Elife,* 1**,** e00205.

LANGMEAD, B. & SALZBERG, S. L. 2012. Fast gapped-read alignment with Bowtie 2. *Nat Methods,* 9**,** 357-9.

LAWRENCE, M., HUBER, W., PAGES, H., ABOYOUN, P., CARLSON, M., GENTLEMAN, R., MORGAN, M. T. & CAREY, V. J. 2013. Software for computing and annotating genomic ranges. *PLoS Comput Biol,* 9**,** e1003118.

LI, H., HANDSAKER, B., WYSOKER, A., FENNELL, T., RUAN, J., HOMER, N., MARTH, G., ABECASIS, G., DURBIN, R. & GENOME PROJECT DATA PROCESSING, S. 2009. The Sequence Alignment/Map format and SAMtools. *Bioinformatics,* 25**,** 2078-9.

LI, H., ILIN, S., WANG, W., DUNCAN, E. M., WYSOCKA, J., ALLIS, C. D. & PATEL, D. J. 2006. Molecular basis for site-specific read-out of histone H3K4me3 by the BPTF PHD finger of NURF. *Nature,* 442**,** 91-5.

LONG, H. K., SIMS, D., HEGER, A., BLACKLEDGE, N. P., KUTTER, C., WRIGHT, M. L., GRUTZNER, F., ODOM, D. T., PATIENT, R., PONTING, C. P., et al. 2013. Epigenetic conservation at gene regulatory elements revealed by non-methylated DNA profiling in seven vertebrates. *Elife,* 2**,** e00348.

LOVE, M. I., HUBER, W. & ANDERS, S. 2014. Moderated estimation of fold change and dispersion for RNA-seq data with DESeq2. *Genome Biol,* 15**,** 550.

MUELLER, F., KARPOVA, T. S., MAZZA, D. & MCNALLY, J. G. 2012. Monitoring dynamic binding of chromatin proteins in vivo by fluorescence recovery after photobleaching. *Methods Mol Biol,* 833**,** 153-76.

PENA, P. V., DAVRAZOU, F., SHI, X., WALTER, K. L., VERKHUSHA, V. V., GOZANI, O., ZHAO, R. & KUTATELADZE, T. G. 2006. Molecular mechanism of histone H3K4me3 recognition by plant homeodomain of ING2. *Nature,* 442**,** 100-3.

PICELLI, S., BJORKLUND, A. K., REINIUS, B., SAGASSER, S., WINBERG, G. & SANDBERG, R. 2014. Tn5 transposase and tagmentation procedures for massively scaled sequencing projects. *Genome Res,* 24**,** 2033-40.

RADLE, B., RUTKOWSKI, A. J., RUZSICS, Z., FRIEDEL, C. C., KOSZINOWSKI, U. H. & DOLKEN, L. 2013. Metabolic labeling of newly transcribed RNA for high resolution gene expression profiling of RNA synthesis, processing and decay in cell culture. *J Vis Exp*.

RAMON-MAIQUES, S., KUO, A. J., CARNEY, D., MATTHEWS, A. G., OETTINGER, M. A., GOZANI, O. & YANG, W. 2007. The plant homeodomain finger of RAG2 recognizes histone H3 methylated at both lysine-4 and arginine-2. *Proc Natl Acad Sci U S A,* 104**,** 18993-8.

RAN, F. A., HSU, P. D., WRIGHT, J., AGARWALA, V., SCOTT, D. A. & ZHANG, F. 2013. Genome engineering using the CRISPR-Cas9 system. *Nat Protoc,* 8**,** 2281-308.

XU, C., BIAN, C., LAM, R., DONG, A. & MIN, J. 2011. The structural basis for selective binding of non-methylated CpG islands by the CFP1 CXXC domain. *Nat Commun,* 2**,** 227.

ZHANG, Y., LIU, T., MEYER, C. A., EECKHOUTE, J., JOHNSON, D. S., BERNSTEIN, B. E., NUSBAUM, C., MYERS, R. M., BROWN, M., LI, W., et al. 2008. Model-based analysis of ChIP-Seq (MACS). *Genome Biol,* 9**,** R137.

ZHOU, J. C., BLACKLEDGE, N. P., FARCAS, A. M. & KLOSE, R. J. 2012. Recognition of CpG island chromatin by KDM2A requires direct and specific interaction with linker DNA. *Mol Cell Biol,* 32**,** 479-89.
